# *In Crystallo* Synthesis of a Triplet Silver Nitrene

**DOI:** 10.1021/jacs.6c05174

**Published:** 2026-05-04

**Authors:** Pritam Roychowdhury, Aishanee Sur, Matthew T. Figgins, Debasmita Dutta, Lauren M. Brown, Serhiy Demeshko, David C. Powers

**Affiliations:** † Department of Chemistry, 14736 Texas A&M University, College Station, Texas 77843, United States; ‡ Institut für Anorganische Chemie, Georg-August-Universität, Tammannstrasse 4, 37077 Göttingen, Germany

## Abstract

Reactive silver nitrenes are proposed intermediates in Ag-catalyzed C–H amination and olefin aziridination reactions. Synthesis and characterization of reactive Ag nitrenes has proven elusive due to fleeting lifetimes and argentophilic aggregation of Ag­(I) precursors in solution. Here, we report the templated *in crystallo* synthesis and characterization of a mononuclear Ag nitrene supported by a tris­(pyrazolyl)­borate ligand. Photolytic N_2_ elimination from the corresponding aryl azide complex yields a structurally defined Ag nitrene with a triplet ground state, as confirmed by crystallography, magnetic measurements, and quantum chemical calculations. This species displays both stoichiometric and catalytic nitrene transfer reactivity, enabling carbazole and indole formation from organic azides. These results leverage *in crystallo* templating to isolate a mononuclear triplet nitrene of Ag and provide a platform to experimentally elucidate ligand-controlled selectivity in Ag-catalyzed nitrene transfer catalysis.

## Graphical abstract

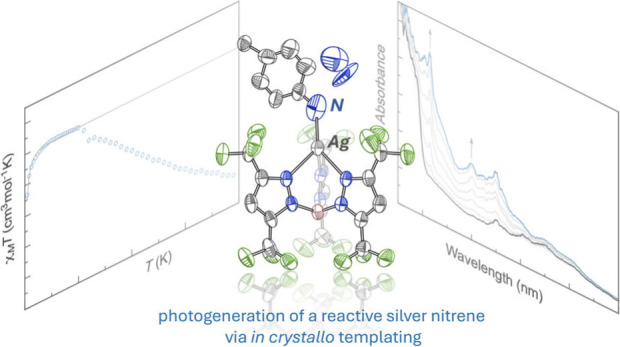

Nitrene transfer catalysis affords nitrogen-containing small molecules from minimally functionalized precursors, such as hydrocarbons and olefins.^1–6^ Among the myriad catalysts that have been developed for nitrene transfer reactions, Ag-catalyzed methods are distinguished by broad substrate tolerance^7–10^ and an exceptional degree of ligand-control on catalyst activity and selectivity (Figure 1a).^11–14^ While computational studies have suggested that catalytically relevant Ag nitrenes display triplet ground states (i.e., ^3^[Ag–NR]),^15^ experimental validation has thus far been unavailable due to (1) the fleeting lifetimes of reactive Ag nitrenes and (2) facile aggregation of Ag nitrene precursors via argentophilic interactions.^16–20^ Isolation of Ag nitrenes has been limited to Bertrand’s report that treatment of an isolable, phosphorus-stabilized nitrene with AgOTf affords mono- and binuclear nitrene adducts (Figure 1b);^21^ neither of these complexes displays nitrene-transfer reactivity.

**Figure 1:**
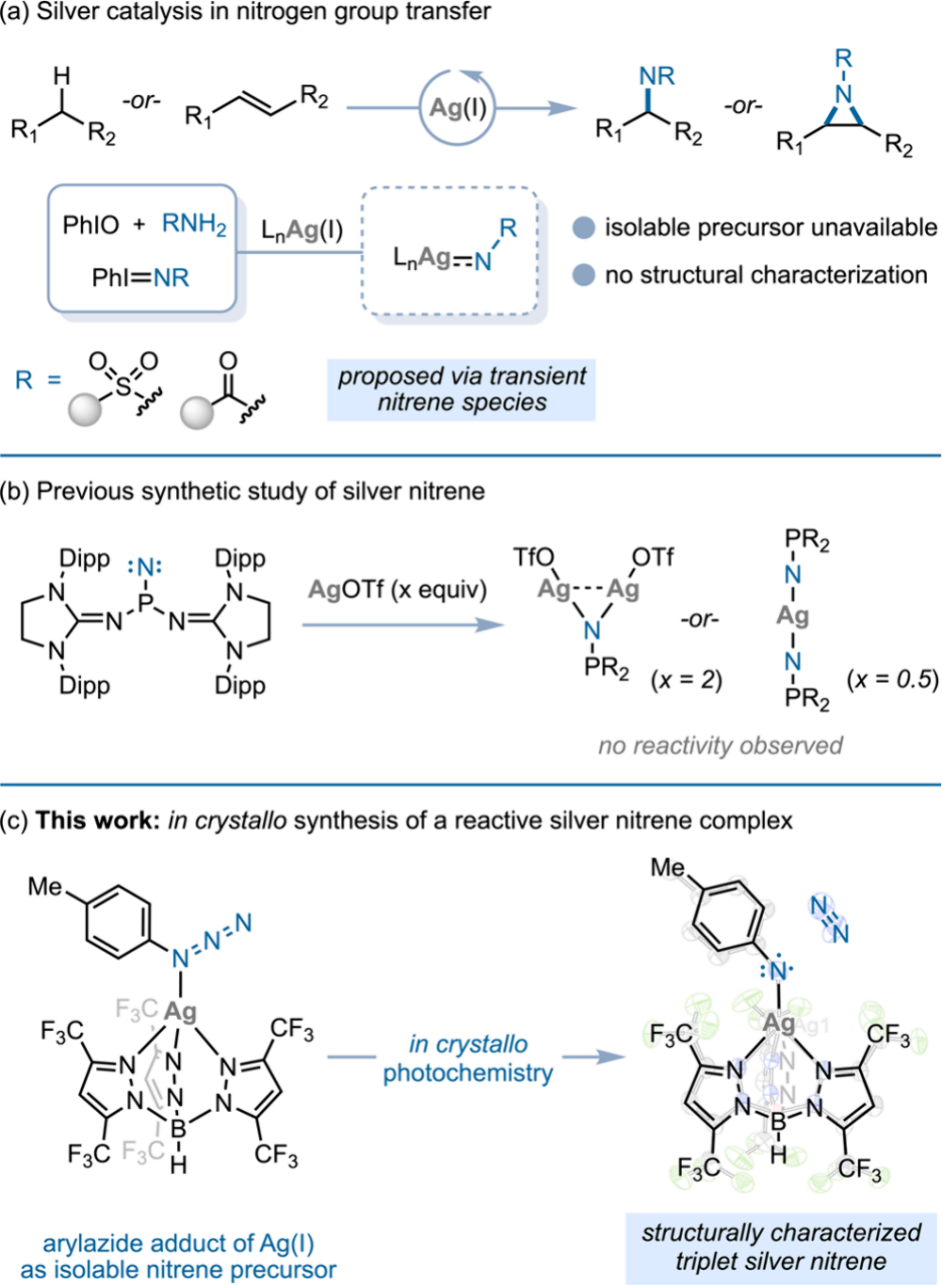
(a) Ag-catalyzed nitrene transfer proceeds via putative Ag nitrene intermediates. (b) Two Ag nitrene complexes have been isolated by treatment of AgOTf with a phosphinonitrene. Neither display nitrene transfer reactivity. (c) We describe the synthesis of a triplet Ag nitrene that is catalytically competent in nitrene transfer.

Here, we describe the synthesis and characterization of a *tris-*pyrazolylborate (Tp)-supported Ag nitrene (i.e., [Tp^CF3^Ag–NTol]) that engages in both stoichiometric and catalytic nitrene transfer (Figure 1c). The nitrene is generated by *in crystallo* photoelimination of N_2_ from a monomeric aryl azide complex of Ag. *In crystallo* synthesis^22–23^ avoids the aggregation equilibria that plague solution-phase experiments by templating the nuclearity of the nitrene by the nuclearity of the photoprecursor. Complementary solid-state optical and magnetic measurements, in combination with quantum chemical calculations, support formulation as a triplet nitrene adduct of Ag­(I). Further, the nitrene is a competent intermediate in catalytic intramolecular C–H amination. Together, these results provide the first example of a reactive Ag nitrene, define the electronic structure of these species as triplets, and demonstrate *in crystallo* templating of nuclearity to access reactive mononuclear species.

We initiated our studies with hydridotris­(3,5-bis­(trifluoromethyl)­pyrazolyl)­borate)-supported Ag­(I) complex **1** AgTp^CF3^(THF) because (1) Tp ligands are often employed in Ag-catalyzed carbene- and nitrene-transfer reactions,^24^ (2) we envisioned facile ligand exchange of the labile THF ligand,^25^ and (3) Tp^CF3^ enforces mononuclear coordination at Ag.^26–28^ Treatment of a pentane solution of **1** with *p*-tolyl azide yielded AgTp^CF3^(4-MePhN_3_) (**2**), a monomeric Ag­(I) complex, as a colorless crystalline solid (72% yield, eq 1). The ^1^H NMR spectrum of **2** (CDCl_3_) displays sharp resonances for the Ag-bound *p*-tolylazide that are well differentiated from those of free *p*-tolylazide (Figure S1). Single-crystal X-ray diffraction (SC-XRD), performed with a sample obtained by slow evaporation of a pentane solution of **2** at −25 °C, indicated *p*-tolylazide coordination to Ag via N_α_. Key metrical parameters include Ag–N1: 2.252(4) Å; N1–C_Ar_: 1.446(6) Å and ∠Ag–N1–C_Ar_ = 128.0(3)°. The ^15^N NMR spectrum of [^15^N]-**2**, prepared from addition of [^15^N]-*p*-tolylazide to **1**, displays two resonances (−138.3 and −142.0 ppm; referenced against nitromethane), which is consistent with enrichment of N1 and N3 (Figure S2). The infrared (IR) spectrum of **2** shows a strong band at 2137 cm^–1^, which shifts to 2114 cm^–1^ in [^15^N]-**2** (Figure S3) (*c.f., p*-tolyl azide shows an absorbance at 2096 cm^–1^).
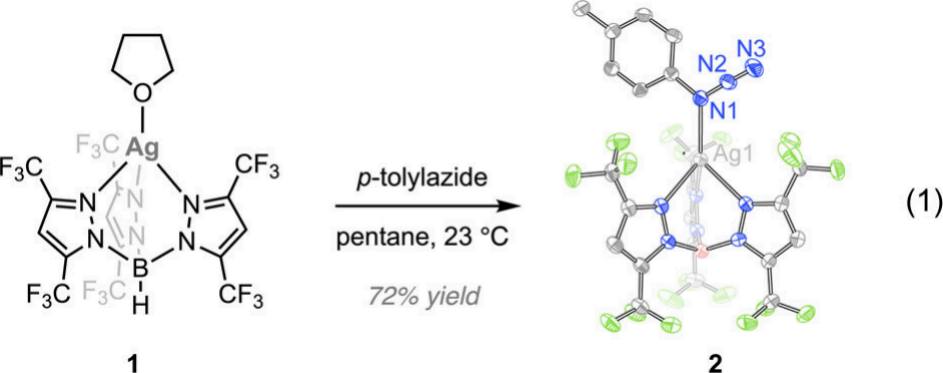


While *p*-tolyl azide is stable at 100 °C, thermal gravimetric analysis (TGA) indicates that N_2_ loss from **2** begins at 100 °C (Figure S4). N_2_ loss from **2** — promoted by either thermolysis (100 °C) or photolysis (370 nm) — is accompanied by the disappearance of the azide stretching mode at 2137 cm^–1^ and by nitrene reactivity (Figure 2a). Thermolysis of a deoxygenated pentane solution of **2** produced AgTp^CF3^(*p*-toluidine) (44% yield, presumably via H atom abstraction from solvent) as well as trace formation of pentane amination products (detected by ESI-MS). Similar results were obtained upon photolysis of a pentane solution of **2** (Figures S5–S7). These data are consistent with the formation of an electrophilic nitrene after N_2_ loss from **2**. In contrast to the results obtained under air-free conditions, thermolysis of **2** in the presence of atmospheric O_2_ afforded orange crystals of [AgTp^CF3^]_2_phenazine (**3**) (24% yield) and AgTp^CF3^(*p*-toluidine) (39%) (Figures 2b and S8). Compound **3** can be envisioned as arising from intermolecular C–H amination followed by aerobic oxidation.

**Figure 2:**
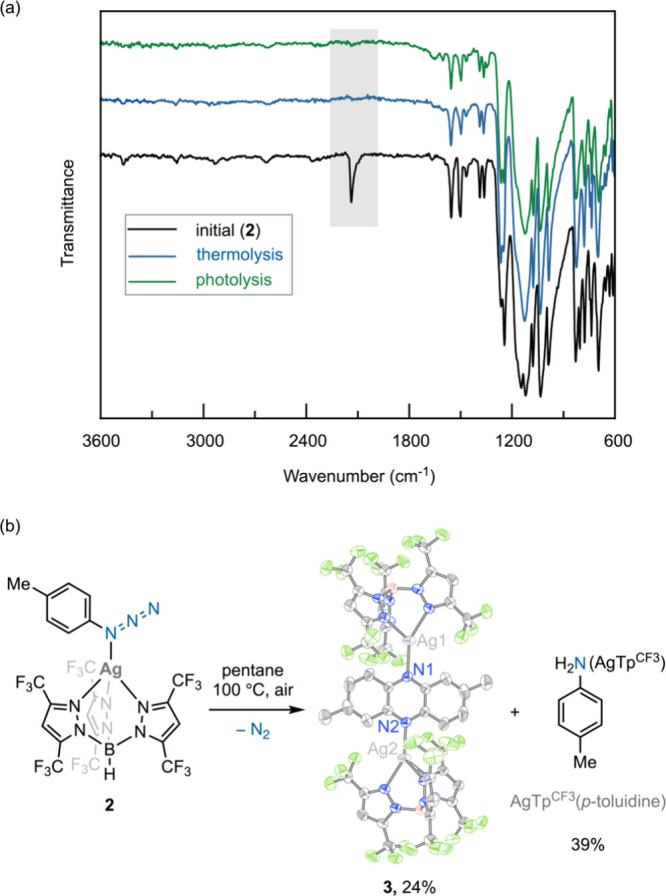
(a) ATR-IR spectrum of **2** after thermolysis and photolysis shows complete disappearance of the azide stretching frequency at 2137 cm^–1^. (b) Thermolysis of **2** under air affords products characteristic of nitrene reactivity. Formation of **3** consumes two equivalents of **2**.

After observing nitrene reactivity following N_2_ elimination from **2**, we turned our attention to the *in crystallo* synthesis of the putative reactive nitrene intermediate. To this end, photolysis (λ = 365 nm) of a single crystal of **2** for 2 min at 100 K promoted *in crystallo* conversion of **2** to Ag nitrene **4** (Figure 3a). Refinement of the data indicated that N_2_ extrusion generates Ag nitrene **4** with 40% chemical conversion.^29^ Further conversion could not be achieved as the crystals degraded at longer photolysis times. N_2_ elimination from **2** is accompanied by a substantial contraction of the Ag–N1 bond length from 2.252(4) Å to 2.13(3) Å. Simultaneously, the N1–C_Ar_ bond contracts from 1.446(6) Å to 1.36(3) Å, and the ∠Ag–N1–C_Ar_ expands from 128.0(3)° to 133(3)°. The evolved N_2_ was refined at 20% occupancy; the lower occupancy of N_2_ as compared to **4** could be due to N_2_ diffusion or N_2_ disorder (Figure 3a).^30^

**Figure 3:**
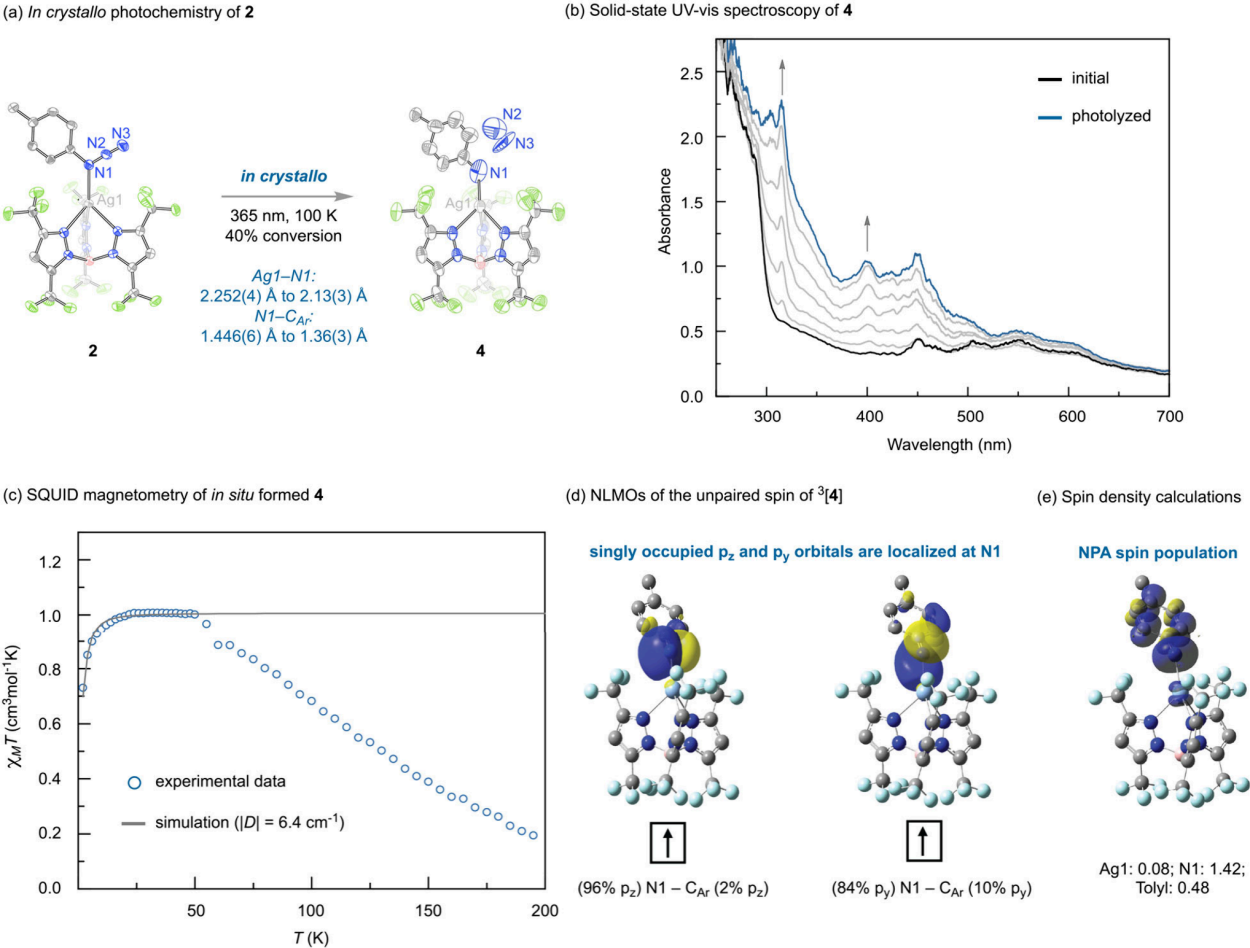
(a) *In crystallo* synthesis of **4**. Selected metrical parameters: Ag–N1: 2.13(3) Å; N1–C_Ar_: 1.36(3) Å. (b) Solid-state UV–vis spectrum of **4** (77 K). (c) Plot of χ_*M*_ *T* vs *T* for ^3^[**4**]. (d) NLMOs from NBO analysis of the two unpaired electrons on nitrogen in ^3^[**4**]. (e) Spin density plot and NPA spin populations from NBO analysis of ^3^[**4**].

*In crystallo* conversion of **2** to **4** is accompanied by a color change from colorless to light yellow. Photolysis (370 nm; 77 K) of a thin film of **2**, obtained by evaporation of a dilute CH_2_Cl_2_ solution, similarly resulted in sample yellowing. Complex **2** exhibits strong ligand-based absorbance below 300 nm with weak features ranging from 300 to 600 nm. New spectral features centered at 315 and 400 nm evolve during photolysis (Figure 3b). These peaks disappear during thermal annealing to 298 K and back to 77 K (Figure S9), demonstrating these features arise from a reactive intermediate (i.e., **4**).

To evaluate the spin ground state of nitrene **4**, photolysis of **2** (350 nm) was performed at 10 K within a SQUID magnetometer, resulting in a time-dependent increase in magnetic moment (Figure S10). Analysis of χ_*M*_ *T* (χ_*M*_ *T* = 0.125*g* ^2^ *S*(*S* + 1)) indicates an *S* = 1 system. This assignment is primarily based on the temperature dependence of χ_*M*_ *T*, which displays a decrease at low temperature that is consistent with zero-field splitting in a triplet state. Fitting the data yields *S* = 1 with |*D*| = 6.4 cm^–1^ (Figure 3c). For |*D*| = 6.4 cm^–1^, the *m*_s_ = ±1 transitions fall outside the X-band field window (0–9000 G).^31^ Consistent with this analysis, ^3^[**4**] does not display an X-band EPR spectrum. In contrast, photolysis of *p*-tolyl azide affords a nitrene species with significantly smaller zero-field splitting, for which an EPR signal is observed centered at 6776 G (Figure S11).^32,33^

The experimentally determined metrical parameters and spectroscopic data are consistent with the results of DFT calculations of ^3^[**4**].^34^ Domain-based local pair natural orbital coupled-cluster calculations with single, double, and perturbative triple excitations (DLPNO–CCSD­(T)) revealed that ^3^[**4**] is lower in energy than ^1^[**4**] (Δ*E*_ST_ = *E*_Singlet_ – *E*_Triplet_ = 20.6 kcal mol^–1^). Single-point gas phase energy corrections at the PBE0-D3/BS2 level of theory to the Gibbs Free Energy show ^3^[**4**] evolves via barrierless loss of N_2_ from ^3^[**2**] (Figure S12). The optimized geometry of ^3^[**4**] is consistent with the *in crystallo* structure: The Ag–N1 and N1–C_Ar_ distances are 2.13(3) Å (expt) vs 2.12 Å (calc) and 1.36(3) Å (expt) vs 1.32 Å (calc), respectively (Table S1). The Ag–N1–C_Ar_ bond angle is measured to be 133(3)° and computed to be 147°. We hypothesize that this difference arises from crystal packing restrictions on *in crystallo* structural reorganization, which prevent full relaxation of **4** to the minimum geometry in the solid state. The cryogenic UV–vis spectrum matched well with time-dependent density functional theory (TD-DFT) calculations^35^ of (^3^[Tp^CF3^Ag–NTol]). Computed transitions at 325 and 406 nm are consistent with new peaks observed experimentally (Figures S13). For computed spectral features of ^1^[**4**], see Supporting Information Figure S14.

Natural Bond Orbital (NBO) analysis of ^3^[**4**] was performed at the PBE0-D3/BS2//PBE0-D3/BS1 level of theory. The valence electrons of nitrogen are engaged in two doubly filled N1_sp_ natural localized molecular orbitals (NLMOs) that comprise the σ-interactions between N1 and Ag and C_Ar_, respectively. In addition, there are two singly occupied orbitals on N1, one is localized (96%) on N1_pz_, and the other delocalized between N1_py_ and the tolyl π-system. Lower energy orbitals are also found that contribute to N1–C_Ar_ π-bonding. Natural population analysis reveals predominately *N*-centered spin populations with 1.42 spin population on N1, 0.08 spin population on Ag, and 0.48 spin population delocalized across the tolyl ring (Figures 3d and S15).

NBO analysis using second-order perturbation theory reveals stabilizing interactions of the nitrene involving both the tolyl ring and the Ag center.^36^ α-Pull interactions of the aryl ring (N1_py_ SOMO → *p*-tolyl π* and N1_pz_ SOMO → *p*-tolyl σ*_C–C_) significantly stabilizes the triplet nitrene, consistent with prior descriptions of a triplet Cu nitrene.^37^ The Ag center also contributes to the nitrene stabilization through two β-push interactions (Ag_dxy_, Ag_dxz_ → N1_pz_ SUMO). Holthausen and Schneider described a similar β-push/α-pull phenomenon in the context of a triplet metallocarbene.^38^ Additional Ag-based α-pull stabilizing interactions are also revealed where the unpaired electrons on the N1_py_ and N1_pz_ orbital donate into the vacant Ag_s_ orbital. The bent geometry at nitrogen facilitates these interactions, which would be attenuated at more linear Ag–N1–C_Ar_ angles (Figure S16).

Demonstration of thermal and photochemical nitrene synthesis from an azide precursor suggested that **1** may be a competent catalyst for nitrene transfer with azides. While organic azides have been extensively employed in C–H amination and olefin aziridination reactions catalyzed by Co,^39–42^ Cu,^37,43,44^ Fe,^45,46^ Rh,^47,48^ Ir,^49^ and Pd^50^ complexes, they have not been demonstrated in conjunction with Ag catalysis.

Heating a 1:1 mixture of the AgTp^CF3^(THF) adduct (**1**) and **5a** at 100 °C affords **6a** in 95% yield; using 10 mol % of **1** gives **6a** in 74% yield, whereas only trace amounts are observed in the absence of the **1**.^51^ The lack of background reaction implies that free biphenyl nitrene is not on-path for catalysis. To the best of our knowledge, this is the first example of C–N bond formation using a silver complex with an organic azide. We also conducted catalytic reactions with **5b** and **5c**, both of which gave the corresponding products in 93% (**6b**) and 96% (**6c**) yields, respectively. Additionally, to assess the competency of **1** in aminating vinylic C­(sp^2^)–H bonds, pentane solutions of substrates **7a**–**7c** were thermolyzed with 10 mol % catalyst **1**, all of which gave the corresponding indoles (**8a**–**8c**) in high yields (Figure 4).

**Figure 4:**
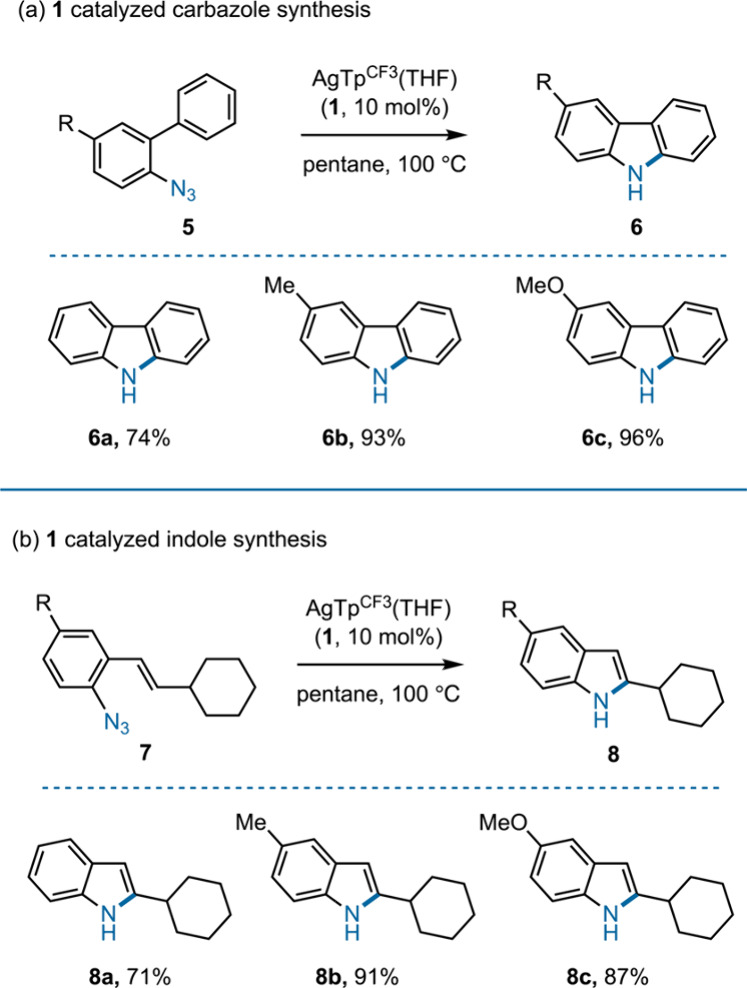
Catalytic intramolecular C–N bond formation using **1** for the synthesis of (a) carbazoles and (b) indoles.

C­(sp^2^)–H amination is typically associated with singlet reactivity.^52,53^ To understand the reaction of ^3^[**4**] with C­(sp^2^)–H bonds, we evaluated a hypothetical reaction between ^3^[**4**] and benzene and found that two-state reactivity—similar to that often encountered in C–H oxygenation at metal-oxo complexes—is likely.^54^ While ^3^[**4**] is 18.4 kcal/mol more stable than ^1^[**4**], the triplet C–H amination transition state is 27.2 kcal/mol higher than the singlet (Figure S17).

In summary, we describe the synthesis and characterization of a mononuclear Tp^CF3^-supported Ag nitrene (^3^[**4**]). The chemical structure of ^3^[**4**] was established by SCXRD by leveraging the molecular templating inherent to *in crystallo* synthesis: Photolysis of a mononuclear photoprecursor provided access to a monomeric nitrene without the possibility for aggregation that has stymied solution-phase syntheses. The electronic structure of ^3^[**4**] was established by cryogenic magnetic and spectroscopic experiments. The experimental and computational data support a triplet ground state. Together, these findings further establish *in crystallo* synthesis as a platform to isolate transient catalytic intermediates and presage systematic experimental evaluation of the ligand-dependent selectivity in Ag-catalyzed group-transfer reactions.

## Supplementary Material



## Notes

Experimental procedures; NMR, IR, UV-vis, and magnetometry data; and refinement details (PDF)
