# Supplementary material for: In Crystallo Synthesis of a Triplet Silver Nitrene
Source: J Am Chem Soc. 2026 May 4;148(18):18627–32. doi: 10.1021/jacs.6c05174 (PMC13185107; doi:10.1021/jacs.6c05174)
Supplement: Supplementary file 1 [file ja6c05174_si_001.pdf]

Supporting Information

In Crystallo Synthesis of a Triplet Silver Nitrene

Pritam Roychowdhury,[‡,a] Aishanee Sur,[‡,a] Matthew T. Figgins,[a] Debasmita Dutta,[a]

Lauren M. Brown,[a] Serhiy Demeshko,[b] and David C. Powers[a,*]

[a]Department of Chemistry, Texas A&M University, College Station, TX 77843, United States

[b]Institut für Anorganische Chemie, Georg-August-Universität, Tammannstrasse 4, 37077 Göttingen, Germany.

Email: powers@chem.tamu.edu

Table of Contents

A. General Considerations S3

B. Synthesis and Characterization S7

C. Supporting Data S21

C.1. Thermogravimetric analysis (TGA) of **2** S21

C.2. Thermolysis and Photolysis of **2** Under N_2 .... S22

C.3. Thermolysis of **2** Under Ambient Atmosphere S26

C.4. Solid-State Photolysis of **2** Monitored by UV-vis Spectroscopy S28

C.5. SQUID Magnetometry S30

C.6. EPR Spectroscopy S31

C.7. Computational Data S32

C.8. IR Spectroscopy S73

C.9. Stoichiometric Reaction with **1** S74

D. General Procedure for Catalytic C–H Amination of Biaryl Azides S76

E. General Procedure for Catalytic C–H Amination of 2-Alkenyl Arylazides S78

F. NMR Spectra S80

G. X-Ray Crystallographic Data S94

H. References S104

A. General Considerations

Materials All the commercial reagents and solvents (ACS reagent grade) were used as received. 3,5-Bis(trifluoromethyl)-1H-pyrazole, 2-bromo-4-methylaniline, 2-bromo-4-methoxyaniline, (*E*)-(2-cyclohexylvinyl)boronic acid, 2-bromoaniline, and phenyl boronic acid were obtained from Ambeed, Inc. Sodium nitrite, sodium azide, Et_2O, sodium sulfate, sodium borohydride, potassium carbonate, ammonium chloride, sodium bicarbonate, toluene, ethanol, dichloromethane, hexanes, and ethyl acetate were purchased from Fisher Scientific. Glacial acetic acid, 2-aminobiphenyl, tetrakis(triphenylphosphine)palladium(0), kerosene, sodium azide-1-^{15}N, and silver trifluoromethanesulfonate were obtained from Sigma Aldrich. Dry pentane and THF (purchased from Fisher scientific, HPLC grade) were obtained from a drying column and stored over activated 4 Å molecular sieves.[1] NMR solvents were purchased from Cambridge Isotope Laboratories and stored over 4 Å molecular sieves. *p*-tolylazide and ^{15}N-labeled *p*-tolylazide were prepared according to literature procedure.[2]

Characterization Details ^{1}H spectral acquisitions were recorded on an Ascend™ 400 NMR (Bruker) and were referenced against residual proteo solvent signals: $CDCl_3$ (7.26 ppm, ^{1}H), C_6D_6 (7.16 ppm, ^{1}H), and DMSO-d_6 (2.50 ppm, ^{1}H).[3] ^{1}H NMR data are reported as follows: chemical shift (δ, ppm), (multiplicity: s (singlet), d (doublet), t (triplet), m (multiplet), br (broad), integration). ^{15}N NMR spectra were recorded in $CDCl_3$ on Bruker Avance NEO 500 NMR operating at 50.7 MHz and acquisitions were referenced against nitromethane as external reference (380.5 ppm). EPR spectra were recorded at X-band (9.35 GHz) on a Bruker ELEXSYS Spectrometer (see below for details).

Thermogravimetric Analysis (TGA) The thermal behavior of **2** was examined by TGA using a TA Instruments TGA5500 system.

Photolysis Details The photolysis experiments were carried out using 370 nm Kessil lamp which was purchased from KESSIL. *In crystallo* photochemistry was carried out using a THORLABS M365FP1 365 nm, 9.8 mW (Min) fiber-coupled LED, 1400 mA.

ATR-IR Spectra were recorded on a Bruker VERTEX 70 spectrometer and were determined as the average of 64 scans.

Photomagnetism Experiments Magnetic measurements on **4** were carried out using a Quantum Design MPMS3 SQUID magnetometer. The photoproduct **4** was formed *in situ* via irradiation of **2** with a TLS120Xe xenon light source, using the fiber optical sample holder (FOSH) to allow optical access to the sample. For this, **2** was placed inside the FOSH and inserted into the magnetometer. The sample was centered in the magnetometer coils and cooled to 2 K. To obtain a background measurement as a raw SQUID voltage response function of the set-up before the reaction, the magnetic moment of **2** in the FOSH was measured from 2 K to 295 K at a magnetic field of 5000 Oe. Afterwards, the sample was cooled back down to 10 K and reaction from **2** to **4** was carried out by photolysis at 350 nm over 140 min. The reaction was monitored by measuring the DC moment of the sample over the whole period of irradiation at a magnetic field of 5000 Oe (Figure S10). This reaction was followed by a temperature-dependent measurement of the reaction product **4** from 2 K to 295 K at a magnetic field of 5000 Oe. The signal that can be attributed to **4** was extracted from the data by subtracting the raw response function of the measured background from the raw response function of the reaction product[4] using the mpView.1.4.1 program.[5] From the resulting magnetic moment, the molar susceptibility of **4** was calculated. As the photolysis yield in **4** is not known, the amount of **4** was varied until the measured $\chi_M T$ product reached the theoretical value for an S = 1 system with g = 2.01, namely $\chi_M T$ = 1 $cm^3 mol^{-1} K$. From this result, formation of 0.07 mg of **4** was calculated. The data was fitted with a spin Hamiltonian approach, using the julX program.[6]

Single Crystal X-ray Diffraction Crystallization details are included in the synthetic procedures for relevant compounds. Crystals suitable for X-ray diffraction were mounted on a MiTeGen dual-thickness micro-mount. The X-ray crystal structure of **2**, **3**, and **4** were collected using a Rigaku XtaLAB Synergy Dualflex HyPix 6000He diffractometer equipped with an Oxford Cryosystems low-temperature device operating at 100 K. The structures were solved with the ShelXT 2018/2 (Sheldrick, 2018) solution program using dual methods and by using Olex2 1.5 (Dolomanov et al., 2009) as the graphical interface. The model was refined with ShelXL 2019/1 (Sheldrick, 2015) using full matrix least squares minimization on F^2.[7-8] All non-hydrogen atoms were refined anisotropically. Hydrogen atom positions were calculated geometrically and refined using the riding model. Disorder was modeled using two parts and appropriate restraints and constraints were added to keep the thermal ellipsoids meaningful. The N–N bond distance in the generated N_2 molecule in **4** was fixed to 1.01 Å.

UV-vis Spectroscopy UV-vis spectrophotometry was carried out using an Ocean Optics USB2000+XR1-ES spectrometer and DH-2000-BAL UV-vis-NIR light source.

Computational Details All geometry optimizations were carried out using Revision C.01 of the Gaussian 16 suite of programs[9] in the gas phase using the PBE0[10] functional in conjunction with Grimme's D3 empirical dispersion[11] and Becke-Johnson damping[12] and the basis set combination (BS1) of the Couty and Hall modification (mod-LANL2DZ)[13] to the basis set of LANL2DZ+ECP combination for Ag[14] and for H, B, C, N, F, the basis set of 6–31G(d').[15-17] All minima were confirmed by analytical frequency computations. Single point energy corrections to optimized geometries were ran using the PBE0 functional with D3BJ using the basis set, BS2 (De2-TZVPP[18] for all atoms plus ECP on Ag.[19]) The singlet-triplet gap ($\Delta E_{S\text{-}T}$) of the nitrenes was calculated with Orca (Version 6.1.1)[20] utilizing domain based local pair natural orbital coupled cluster method with single-, double-, and perturbative triple excitations with def2-TZVPP and De2-TZVPP auxiliary basis sets.[21] Natural Bond Orbital Analysis was conducted using Gaussian 16 utilizing the NBO version 7.[22] UV–vis absorption

spectra was simulated using TD-DFT[23] single points employing PBE0-D3BJ-SMD(pentane)/Def2-TZVPP on the PBE0-D3BJ/BS1-optimized geometries. The first 30 vertical excitations were solved iteratively. The simulated spectra were generated using an in-house coded Fortran program.[24] All orbital images were generated using GaussView6[25] with an isovalue of 0.05.

B. Synthesis and Characterization

Synthesis of Tp^{CF3}

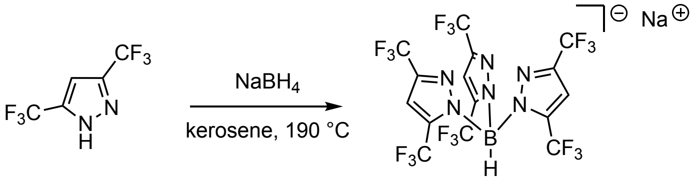

1'

Tp^{CF3} ([HB(3,5-$(CF_3)_2$Pz$)_3$]Na$(H_2O)_n$) was synthesized according to the following modification of literature methods.[26] A 100-mL thick-walled glass tube was charged with a magnetic stir bar, 3,5-bis(trifluoromethyl)-1H-pyrazole (1.0 g, 4.9 mmol, 1.0 equiv), sodium borohydride (83 mg, 2.2 mmol, 0.45 equiv), and kerosene (0.60 mL) under an N_2 atmosphere. The reaction vessel was closed with a Teflon screw top fitted with an appropriate O-ring. The mixture was heated at 190 °C and stirred for 24 h. During this time, the product (**1'**) deposited on the walls of the reaction vessel above the reaction mixture as colorless crystals. The reaction vessel was allowed to cool to 23 °C. The sublimed crystals were collected from the tube and transferred into a 20-mL scintillation vial. Dry pentane (2 mL) was added to the crystalline material, and the resulting suspension was agitated by sonication. The solids were allowed to settle, the supernatant was decanted, and 2 mL of fresh dry pentane was added. The sonication, settling, decantation procedure was repeated twice more. The resulting solid was dried under vacuum at 23 °C to afford **1'** as a colorless solid (601 mg, 57% yield). ^{1}H NMR (δ, 23 °C, 400 MHz, C_6D_6): 6.25 (s, 3H), 0.76 (s, 1H). The obtained spectral data are in good agreement with those reported in literature.[26] Note: The intensity of the trapped H_2O peak at 0.76 ppm varies between batches and does not significantly affect the subsequent steps.

Synthesis of AgTp^{CF3}(THF)

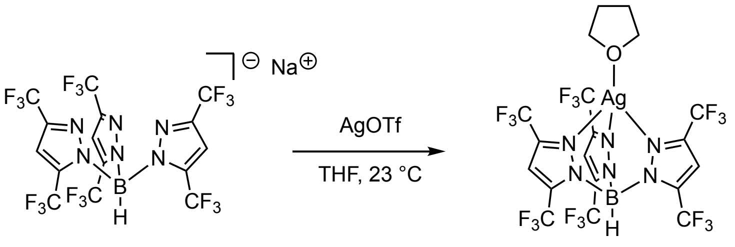

Complex **1** was synthesized according to the following modification of literature methods.[27] A 20-mL scintillation vial was charged with a magnetic stir bar, Tp^{CF3} (0.60 g, 0.93 mmol, 1.0 equiv), silver triflate (0.26 g, 1.0 mmol, 1.1 equiv), and dry THF (2.0 mL). The reaction vial was sealed under an N_2 atmosphere and was wrapped with tape to exclude ambient light. The reaction mixture was stirred at 23 °C for 16 h. The tape was removed; the following work up procedure was carried out under ambient conditions. The reaction mixture was dried under vacuum, then dry pentane (10 mL) was added. The resulting suspension was agitated by sonication, filtered using a syringe filter, and the resulting solution was then dried under vacuum. The sonication, filtration, and drying process was repeated a second time to afford **1** as a colorless solid (0.36 g, 48% yield). Complex **1** was routinely stored at –25 °C under N_2. ^1H NMR (δ, 23 °C, 400 MHz, C_6D_6): 6.27 (s, 3H), 3.59–3.56 (m, 4H), 1.43–1.40 (m, 4H). ^{19}F NMR (δ, 23 °C, 376 MHz, C_6D_6): –58.4, –61.1. The obtained spectral data are in good agreement with those reported in literature.[27]

Synthesis of Ag(Tp^{CF3})(4-MePhN$_3$)

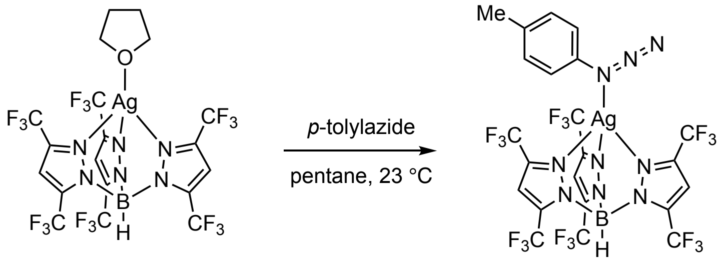

1 2

A 20-mL scintillation vial was charged with a magnetic stir bar, complex **1** (0.24 g, 0.30 mmol, 1.0 equiv), *p*-tolylazide (48 mg, 0.36 mmol, 1.2 equiv), and dry pentane (5.0 mL) in an N_2-filled glovebox. The reaction vial was wrapped with aluminum foil to exclude ambient light. The reaction mixture was stirred at 23 °C for 16 h. Solids were removed by filtration through a syringe filter. The filtrate was concentrated to ~0.5 mL total volume under vacuum. The concentrated solution was kept inside a glovebox freezer at –25 °C. Prism shaped colorless crystals formed after 24 h. The mother liquor was decanted, and the crystals were quickly washed with dry pentane (2 × 0.5 mL). The crystals were then dried under vacuum for 16 h at 23 °C to yield **2** as a colorless solid (0.19 g, 72% yield). Complex **2** was routinely stored at –25 °C under N_2 in foil-wrapped vials to exclude ambient light. ^1H NMR (δ, 23 °C, 400 MHz, $CDCl_3$): 7.18 (d, *J* = 8.0 Hz, 2H), 6.97 (d, *J* = 8.4 Hz, 2H), 6.92 (s, 3H), 2.35 (s, 3H). ^{19}F NMR (δ, 23 °C, 376 MHz, $CDCl_3$): –58.9, –61.9.

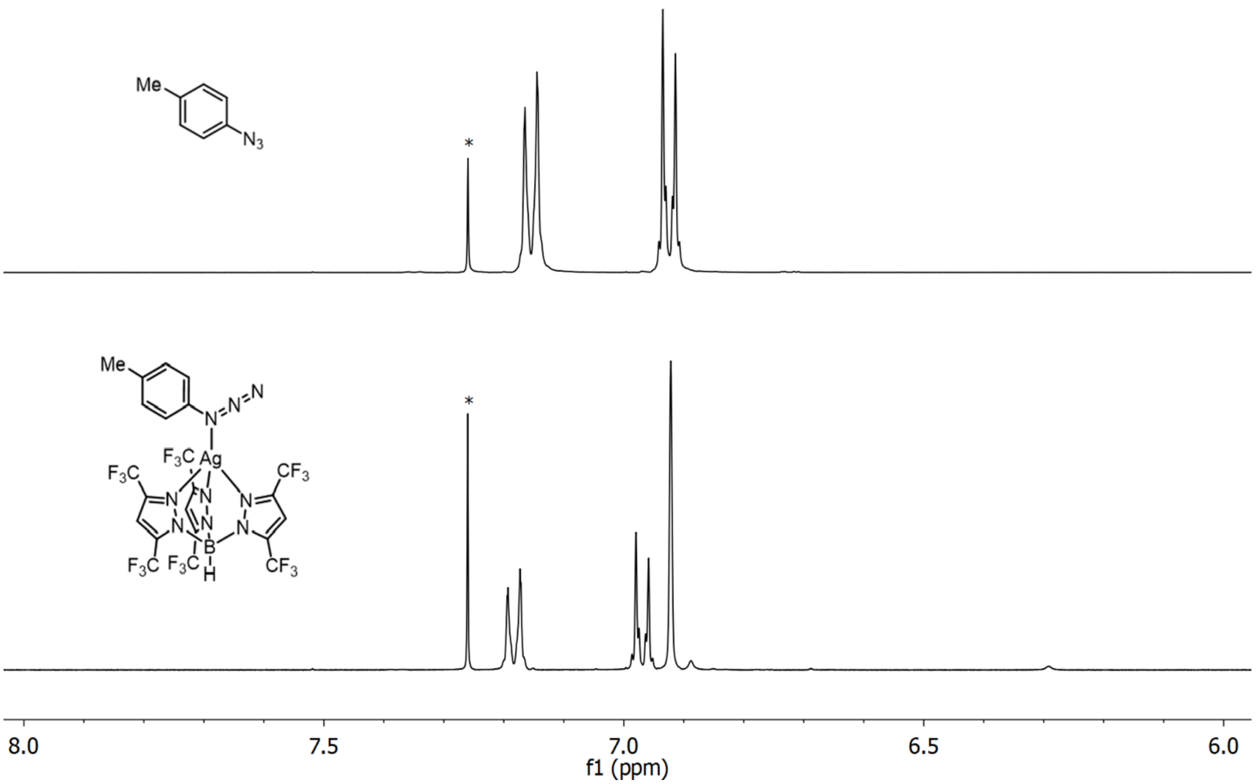

Figure S1. ^{1}H NMR spectrum of **2** (bottom) in $CDCl_3$ (400 MHz, 23 °C), overlaid with that of *p*-tolylazide (top) recorded under identical conditions. The overlay shows a downfield shift of the aryl C–H resonances upon coordination with Ag. Asterisks (*) indicate the residual $CDCl_3$ solvent peak at 7.26 ppm.

Synthesis of $(Tp^{CF3})Ag(4\text{-}MePh^{15}NN_2)$

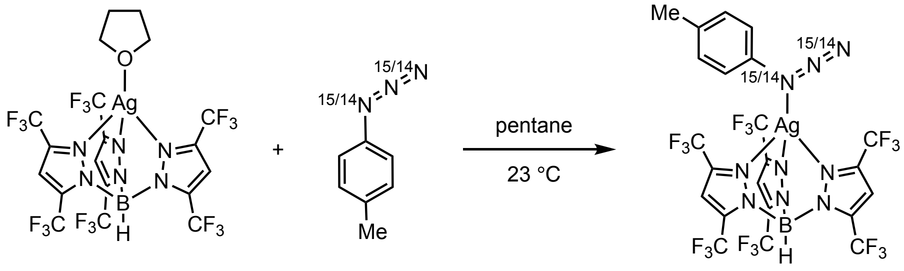

1

^{15}N-**2**

A 20-mL scintillation vial was charged with a magnetic stir bar, complex **1** (0.24 g, 0.30 mmol, 1.0 equiv), ^{15}N-labeled *p*-tolylazide (48 mg, 0.36 mmol, 1.2 equiv), and dry pentane (5.0 mL) in an N_2-filled glovebox. The reaction vial was wrapped with aluminum foil to exclude ambient light. The reaction mixture was stirred at 23 °C for 16 h. Solids were removed by filtration through a syringe filter. The filtrate was concentrated to ~0.5 mL total volume under vacuum. The concentrated solution was kept inside a glovebox freezer at –25 °C. Colorless prismatic were obtained after 24 h. The mother liquor was decanted, and the crystals were quickly washed with dry pentane (2 × 0.5 mL). The crystals were then dried under vacuum for 16 h at 23 °C to yield ^{15}N-**2** as a colorless solid (0.19 g, 76% yield). ^{1}H NMR (δ, 23 °C, 400 MHz, $CDCl_3$): 7.18 (d, *J* = 8.0 Hz, 2H), 6.97 (d, *J* = 8.4 Hz, 2H), 6.92 (s, 3H), 2.35 (s, 3H). ^{19}F NMR (δ, 23 °C, 376 MHz, $CDCl_3$): –58.9, –61.9. ^{15}N{^{1}H} NMR (δ, 23 °C, 50.7 MHz, $CDCl_3$): –138.3, –142.0.

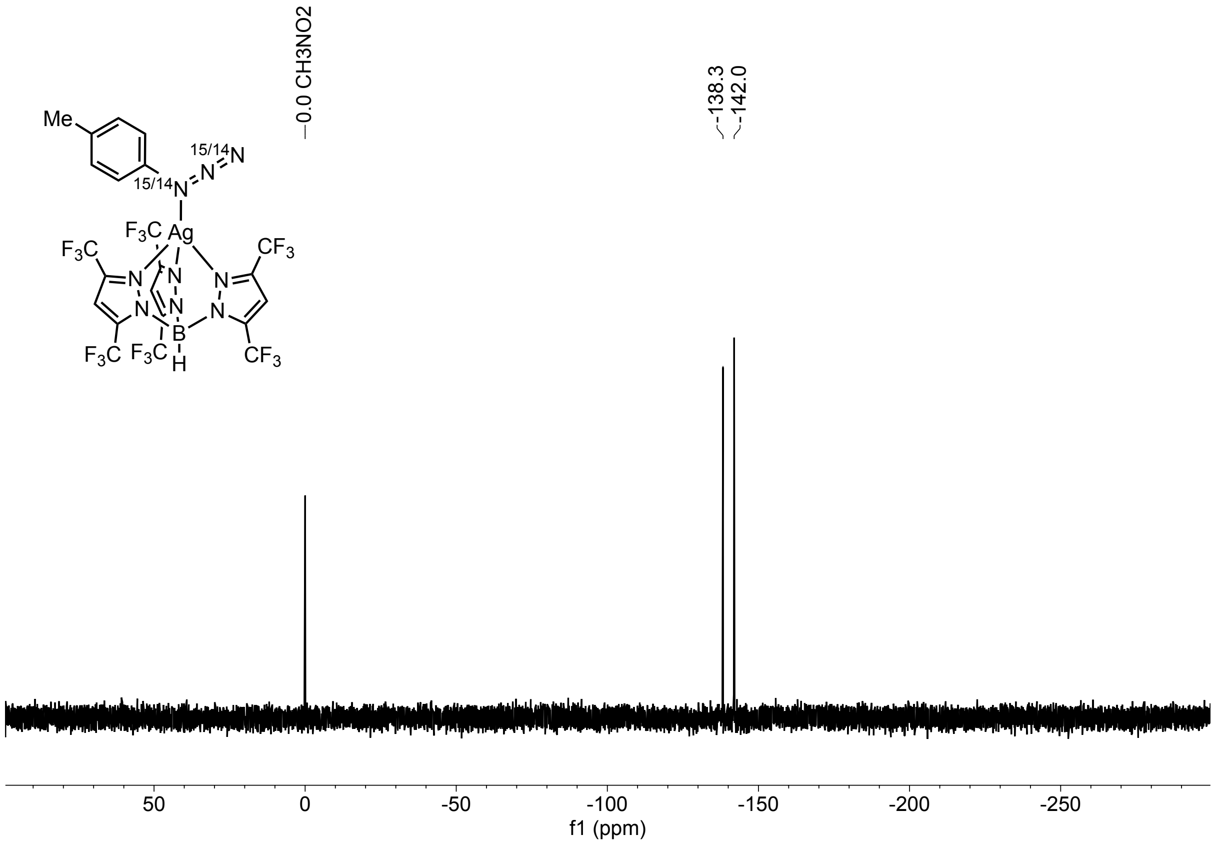

Figure S2. $^{15}N\{^{1}H\}$ NMR spectrum of ^{15}N-**2** in $CDCl_3$ (50.7 MHz) at 23 °C referenced against CH_3NO_2 at 0 ppm.

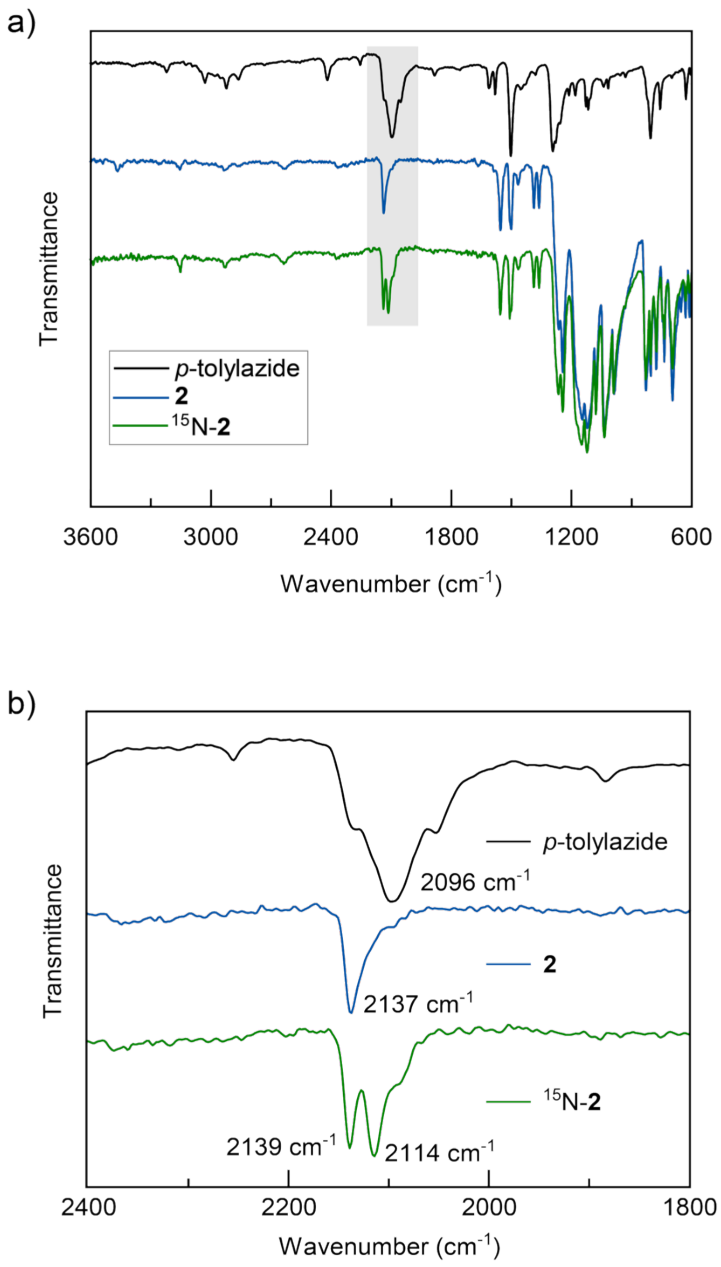

Figure S3. a) Solid-state ATR-IR spectra of **2**, *p*-tolylazide, and ^{15}N-labeled **2.** b) Expanded region of the azide stretching frequencies show complete ligation of *p*-tolylazide to [Ag].

General Procedure for Synthesis of Biaryl Anilines

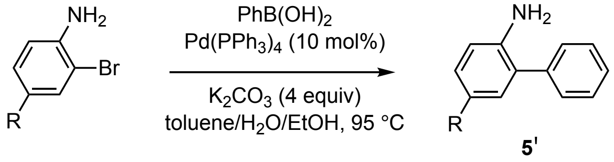

The following biaryl anilines were prepared according to the following modification of literature methods.[28] A 50-mL round bottom flask was charged with a magnetic stir bar, phenyl boronic acid (0.39 g, 3.2 mmol, 1.3 equiv), K_2CO_3 (1.4 g, 10 mmol, 4.0 equiv), and $Pd(PPh_3)_4$ (0.29 g, 0.25 mmol, 0.10 equiv). N_2 purged toluene (12.0 mL), deionized water (8.0 mL), and ethanol (4.0 mL) were added to the reaction vessel. The appropriate aniline derivative (2.5 mmol, 1.0 equiv) was then added. The resulting mixture was heated at 95 °C for 16 h. The reaction mixture was allowed to cool to 23 °C. The resulting biphasic mixture was diluted with saturated aqueous NH_4Cl (30 mL) and dichloromethane (30 mL). The layers were separated. The aqueous phase was further extracted with dichloromethane (2 × 30 mL), and the combined organic layers were washed with water (30 mL) and saturated aqueous $NaHCO_3$ (30 mL). The organic phase was dried over Na_2SO_4 and filtered. The filtrate was concentrated in vacuo to afford the crude product. Purification by flash column chromatography on silica gel (hexanes / ethyl acetate) afforded the corresponding product.

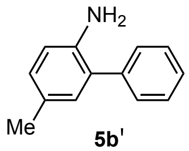

5-methyl-[1,1'-biphenyl]-2-amine (5b') Prepared from 2-bromo-4-methylaniline and obtained as a pale-yellow solid (0.23 g, 50% yield). ^1H NMR (δ, 23 °C, 400 MHz, $CDCl_3$): 7.47–7.40 (m, 4H), 7.37–7.30 (m, 1H), 7.01–6.94 (m, 2H), 6.70 (d, J = 7.9 Hz, 1H), 3.63 (br s, 2H), 2.28 (s, 3H). The obtained spectral data are in good agreement with those reported in literature.[28]

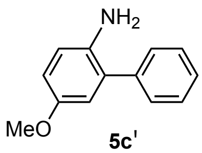

5c'

5-methoxy-[1,1'-biphenyl]-2-amine (5c') Prepared from 2-bromo-4-methoxyaniline and obtained as a pale-yellow solid (0.35 g, 70% yield). ^{1}H NMR (δ, 23 °C, 400 MHz, $CDCl_3$): 7.50–7.40 (m, 4H), 7.39–7.32 (m, 1H), 6.81–6.70 (m, 3H), 3.77 (s, 3H), 3.50 (br s, 2H). The obtained spectral data are in good agreement with those reported in literature.[28]

General Procedure for Synthesis of Biaryl Azides

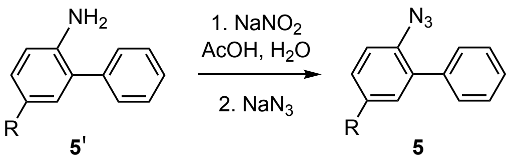

The following biaryl azides were prepared according to the following modification of literature methods.[28] A 50-mL round bottom flask was charged with a magnetic stir bar, the appropriate 2-aminobiaryl derivative (1.5 mmol, 1.0 equiv), and a mixture of acetic acid / deionized water (10 mL / 5 mL) at 0 °C. $NaNO_2$ (0.15 g, 2.1 mmol, 1.4 equiv) was added to the reaction vessel slowly and the resulting mixture was stirred at 0 °C for 1 h. NaN_3 (0.15 g, 2.3 mmol, 1.5 equiv) was then added slowly. The resulting mixture was warmed to 23 °C and was stirred overnight. The solution was diluted with water (30 mL) and Et_2O (30 mL) and neutralized by the slow addition of saturated aqueous $NaHCO_3$ solution. The phases were separated, and the aqueous phase was extracted with Et_2O (2 × 30 mL). The combined organic phase was washed with water (1 × 30 mL) and brine (1 × 30 mL). The organic phases were dried over Na_2SO_4 and filtered. The filtrate was concentrated in vacuo to afford the crude product. Purification by flash column chromatography on silica gel (hexanes / ethyl acetate) afforded the corresponding product.

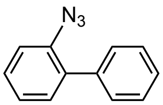

5a

2-azido-1,1'-biphenyl (5a) Prepared from [1,1'-biphenyl]-2-amine and obtained as a pale-yellow solid (0.27 g, 91% yield). ^{1}H NMR (δ, 23 °C, 400 MHz, $CDCl_3$): 7.46–7.34 (m, 7H), 7.28–7.20 (m, 2H). The obtained spectral data are in good agreement with those reported in literature.[28]

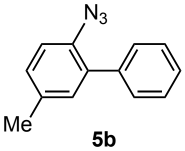

5b

2-azido-5-methyl-1,1'-biphenyl (5b) Prepared from 5-methyl-[1,1'-biphenyl]-2-amine (**5b'**) and obtained as a yellow oil (0.21 g, 68% yield). ^{1}H NMR (δ, 23 °C, 400 MHz, $CDCl_3$): 7.46–7.40 (m, 4H), 7.39–7.32 (m, 1H), 7.22–7.17 (m, 1H), 7.16–7.12 (m, 2H), 2.37 (s, 3H). The obtained spectral data are in good agreement with those reported in literature.[28]

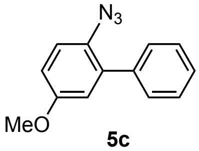

5c

2-azido-5-methoxy-1,1'-biphenyl (5c) Prepared from 5-methoxy-[1,1'-biphenyl]-2-amine (**5c'**) and obtained as a brown oil (0.30 g, 88% yield). ^{1}H NMR (δ, 23 °C, 400 MHz, $CDCl_3$): 7.47–7.41 (m, 4H), 7.40–7.35 (m, 1H), 7.17 (d, *J* = 8.7 Hz, 1H), 6.94 (dd, *J* = 8.7, 2.9 Hz, 1H), 6.88 (d, *J* = 2.9 Hz, 1H), 3.83 (s, 3H). The obtained spectral data are in good agreement with those reported in literature.[28]

General Procedure for Synthesis of 2-Alkenylanilines

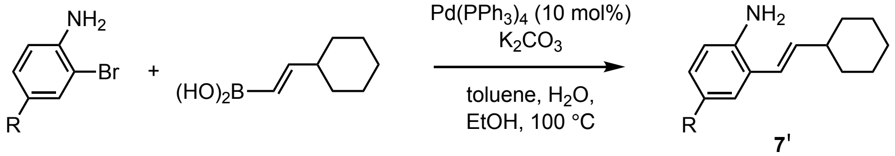

The following 2-alkenylanilines were prepared according to the following modifications of literature methods.[29] A 25-mL Schlenk flask was charged with a magnetic stir bar, (E)-(2-cyclohexylvinyl)boronic acid (0.20 g, 1.3 mmol, 1.5 equiv), K_2CO_3 (0.47 g, 3.4 mmol, 4.0 equiv), and $Pd(PPh_3)_4$ (0.1 mg, 0.09 mmol, 0.1 equiv). The atmosphere within the reaction flask was exchanged with N_2 by three evacuation-backfill cycles. Degassed toluene (26 mL), deionized water (2.5 mL), EtOH (5.0 mL), and the appropriate aniline (0.86 mmol, 1.0 equiv) were then added to the reaction vessel and heated at 100 °C for 16 h. The reaction mixture was cooled to 23 °C. The biphasic mixture was diluted with water (30 mL) and dichloromethane (30 mL). The aqueous phase was further extracted with dichloromethane (2 × 30 mL), and the combined organic layers were washed with water (30 mL) and brine (30 mL). The organic phases were dried over Na_2SO_4 and filtered. The filtrate was concentrated in vacuo to afford the crude product. Purification by flash column chromatography on silica gel (hexanes / ethyl acetate) afforded the corresponding product.

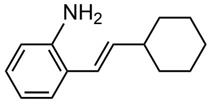

7a'

(E)-2-(2-cyclohexylvinyl)aniline (7a') Prepared from 2-bromoaniline and obtained as a pale-yellow liquid (0.16 g, 95% yield). ^1H NMR (δ, 23 °C, 400 MHz, $CDCl_3$): 7.22 (dd, *J* = 7.6, 1.5 Hz, 1H), 7.05–7.01 (m, 1H), 6.76–6.72 (m, 1H), 6.67–6.65 (m, 1H), 6.38–6.34 (m, 1H), 6.03 (dd, *J* = 15.8, 7.0 Hz, 1H), 3.69 (br s, 2H), 2.15-2.10 (m, 1H), 1.84–1.66 (m, 5H), 1.35–1.17 (m, 5H). The obtained spectral data are in good agreement with those reported in literature.[29]

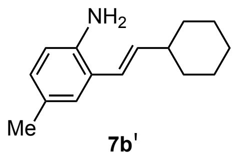

7b'

(*E*)-2-(2-cyclohexylvinyl)-4-methylaniline (7b') Prepared from 2-bromo-4-methylaniline and obtained as a colorless solid (0.18 g, 97% yield). ^{1}H NMR (δ, 23 °C, 400 MHz, $CDCl_3$): 7.06 (d, *J* = 1.4 Hz, 1H), 6.86 (dd, *J* = 8.0, 1.8 Hz, 1H), 6.59 (d, *J* = 8.0 Hz, 1H), 6.36 (d, *J* = 15.8 Hz, 1H), 6.03 (dd, *J* = 15.8, 7.0 Hz, 1H), 3.52 (br s, 2H), 2.25 (s, 3H), 2.20–2.07 (m, 1H), 1.87–1.64 (m, 5H), 1.41–1.13 (m, 5H). ^{13}C NMR (δ, 23 °C, 100 MHz, $CDCl_3$): 140.9, 138.9, 128.5, 128.3, 127.8, 124.7, 122.9, 116.2, 41.7, 33.2, 26.3, 26.2, 20.6.

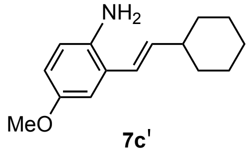

7c'

(*E*)-2-(2-cyclohexylvinyl)-4-methoxyaniline (7c') Prepared from 2-bromo-4-methoxyaniline and obtained as a dark-red oil (0.18 mg, 93% yield). ^{1}H NMR (δ, 23 °C, 400 MHz, $CDCl_3$): 6.82 (d, *J* = 2.7 Hz, 1H), 6.69–6.58 (m, 2H), 6.37 (dd, *J* = 15.8, 1.4 Hz, 1H), 6.04 (dd, *J* = 15.8, 7.0 Hz, 1H), 3.76 (s, 3H), 3.37 (br s, 2H), 2.19–2.09 (m, 1H), 1.85–1.62 (m, 5H), 1.38–1.13 (m, 5H). ^{13}C NMR (δ, 23 °C, 100 MHz, $CDCl_3$): 153.2, 139.3, 137.1, 125.9, 122.8, 117.4, 113.9, 112.3, 55.9, 41.6, 33.2, 26.3, 26.1.

General Procedure for Synthesis of 2-Alkenyl Aryl Azides

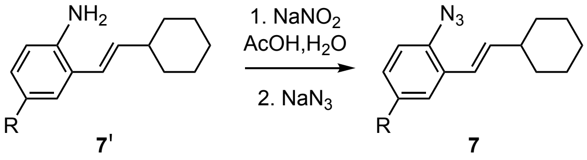

The following 2-alkenyl aryl azides were prepared according to the following modification of literature methods.[28] A 50-mL round bottom flask was charged with a magnetic stir bar, the appropriate 2-alkenylaniline derivative (0.45 mmol, 1.0 equiv), and a mixture of acetic acid / deionized water (3.0 mL / 1.5 mL) at 0 °C. $NaNO_2$ (43 mg, 0.63 mmol, 1.4 equiv) was added slowly and the resulting mixture was stirred at 0 °C for 1 h. NaN_3 (44 mg, 0.68 mmol, 1.5 equiv) was then added slowly, and the resulting mixture was warmed to 23 °C and stirred for 1 h. The solution was diluted with water and pentane. The phases were separated, and the organic layer was neutralized by the slow addition of saturated aqueous $NaHCO_3$, the phases were separated, and the aqueous phase was extracted with pentane (2 × 30 mL). The combined organic phase was washed with water (1 × 50 mL) and brine (1 × 50 mL). The organic phases were dried over Na_2SO_4 and filtered. The filtrate was concentrated in vacuo to afford the crude product. Purification by flash column chromatography on silica gel (hexanes / ethyl acetate) afforded the corresponding products.

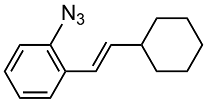

7a

(*E*)-1-azido-2-(2-cyclohexylvinyl)benzene (7a) Prepared from (*E*)-2-(2-cyclohexylvinyl)aniline (**7a'**) and obtained as a pale-yellow oil (80 mg, 78% yield). ^1H NMR (δ, 23 °C, 400 MHz, $CDCl_3$): 7.48–7.45 (m, 1H), 7.22 (dd, *J* = 7.3, 1.4 Hz, 1H), 7.12–7.05 (m, 2H), 6.56 (d, *J* = 16.1 Hz, 1H), 6.16 (dd, *J* = 16.0, 7.0 Hz, 1H), 2.19–2.11 (m, 1H), 1.83–1.66 (m, 5H), 1.35–1.14 (m, 5H). The obtained spectral data are in good agreement with those reported in literature.[28]

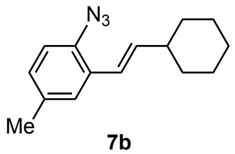

7b

(*E*)-1-azido-2-(2-cyclohexylvinyl)-4-methylbenzene (7b) Prepared from (*E*)-2-(2-cyclohexylvinyl)-4-methylaniline (**7b'**) and obtained as a colorless solid (98 mg, 90% yield). ^{1}H NMR (δ, 23 °C, 400 MHz, $CDCl_3$): 7.28 (s, 1H), 7.08–6.97 (m, 2H), 6.54 (dd, *J* = 16.0, 1.0 Hz, 1H), 6.15 (dd, *J* = 16.0, 7.0 Hz, 1H), 2.32 (s, 3H), 2.20–2.09 (m, 1H), 1.87–1.64 (m, 5H), 1.42–1.13 (m, 5H). ^{13}C NMR (δ, 23 °C, 100 MHz, $CDCl_3$): 138.7, 134.5, 134.0, 129.7, 128.8, 127.0, 121.9, 118.4, 41.6, 33.1, 26.3, 26.2, 21.1.

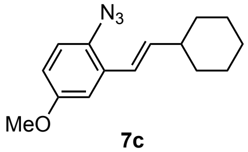

7c

(*E*)-1-azido-2-(2-cyclohexylvinyl)-4-methoxybenzene (7c) Prepared from (*E*)-2-(2-cyclohexylvinyl)-4-methoxyaniline (**7c'**) and obtained as a yellow solid (90 mg, 78% yield). ^{1}H NMR (δ, 23 °C, 400 MHz, $CDCl_3$): 7.06–6.95 (m, 2H), 6.80 (dd, *J* = 8.7, 2.9 Hz, 1H), 6.55 (dd, *J* = 16.0, 1.2 Hz, 1H), 6.15 (dd, *J* = 16.0, 7.0 Hz, 1H), 3.81 (s, 3H), 2.21–2.09 (m, 1H), 1.88–1.63 (m, 5H), 1.40–1.14 (m, 5H). ^{13}C NMR (δ, 23 °C, 100 MHz, $CDCl_3$): 156.9, 139.1, 130.9, 129.5, 121.8, 119.5, 114.0, 111.3, 55.7, 41.5, 33.0, 26.3, 26.1.

C. Supporting Data

C.1. Thermogravimetric analysis (TGA) of 2

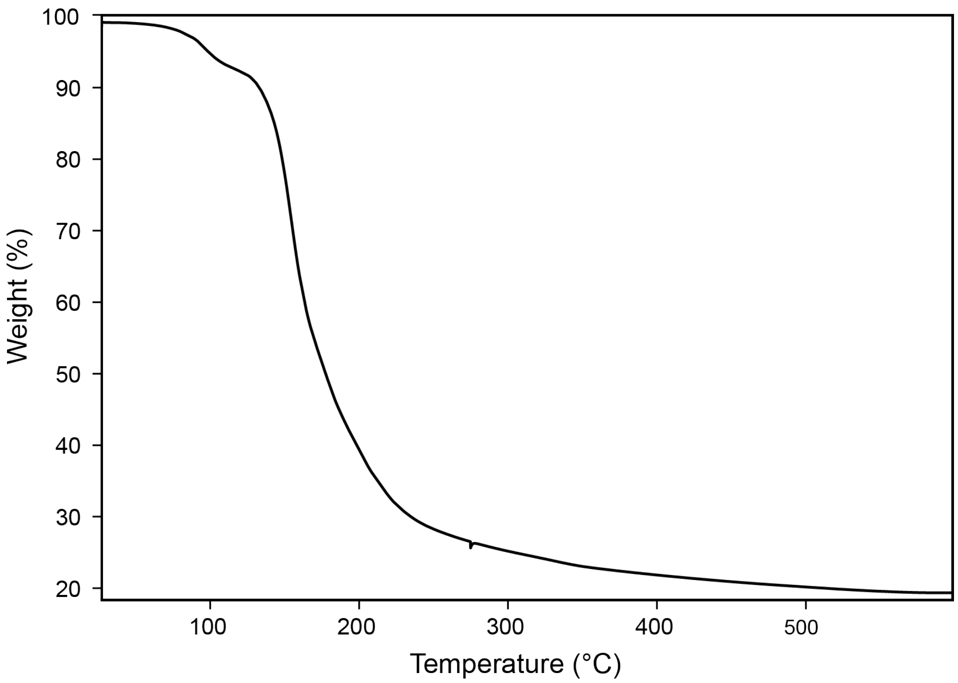

Figure S4. Thermogravimetric analysis (TGA) of **2** collected under a nitrogen atmosphere with a heating rate of 5 °C min^{-1} from 23 °C to 600 °C. The sample mass was 1.44 mg, and data were collected at 0.1 s intervals.

C.2. Thermolysis and Photolysis of 2 Under N_2

In an N_2-filled glovebox, a pentane solution (2.0 mL) of **2** (10 mg) was loaded into a 25-mL Schlenk tube. The solution was then heated at 100 °C for 16 h. After that time the reaction mixture was concentrated in vacuum and subsequently taken up in $CDCl_3$ and the crude reaction mixture was analyzed by ^1H NMR, ESI-MS. The crude ^1H NMR showed 44% yield of the Ag(Tp^{CF3})(*p*-toluidine) using trimethoxybenzene as the internal standard.

In an N_2-filled glovebox, a pentane solution (2.0 mL) of **2** (10 mg) was loaded into a 25-mL Schlenk tube. The solution was photolyzed (λ = 370 nm) for 16 h. After that time the reaction mixture was concentrated in vacuum and subsequently taken up in $CDCl_3$ and the crude reaction mixture was analyzed by ^1H NMR, ESI-MS. The crude ^1H NMR showed 40% yield of the Ag(Tp^{CF3})(*p*-toluidine) using trimethoxybenzene as the internal standard.

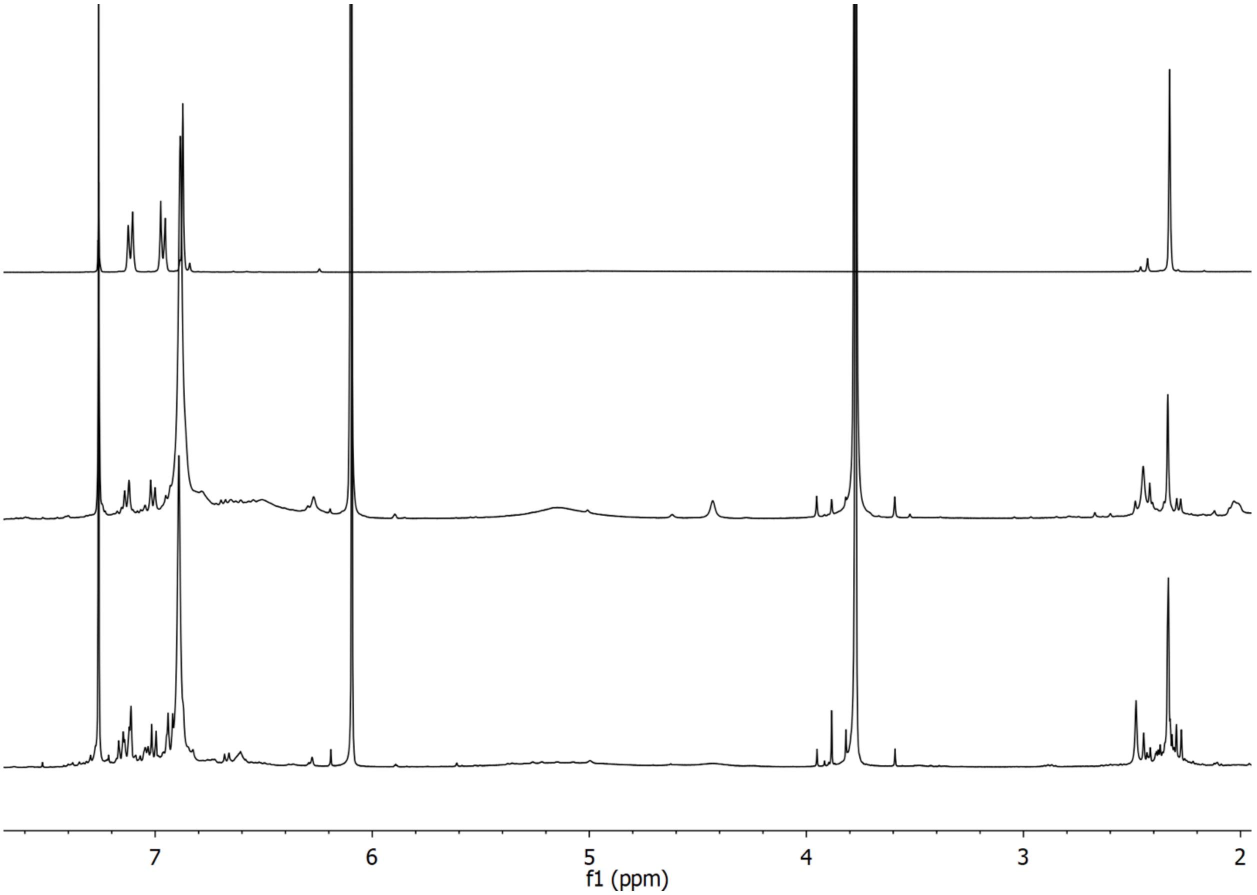

Figure S5. Crude ^{1}H NMR spectra obtained following thermolysis (bottom) and photolysis (middle) of **2**, compared with an authentic sample of *p*-toluidine bound to AgTp^{CF3} (top). Coordination to AgTp^{CF3} shifts the *p*-toluidine resonances, consistent with signals observed in both crude reaction mixtures. Crude yields were determined using trimethoxybenzene as an internal standard by integrating the 6.09 ppm resonance against the doublet at 7.00 ppm. HRMS (ESI) analysis of the crude mixture also confirmed formation of *p*-toluidine (calcd for $[M+H]^+$ = 108.0813; found = 108.0811).

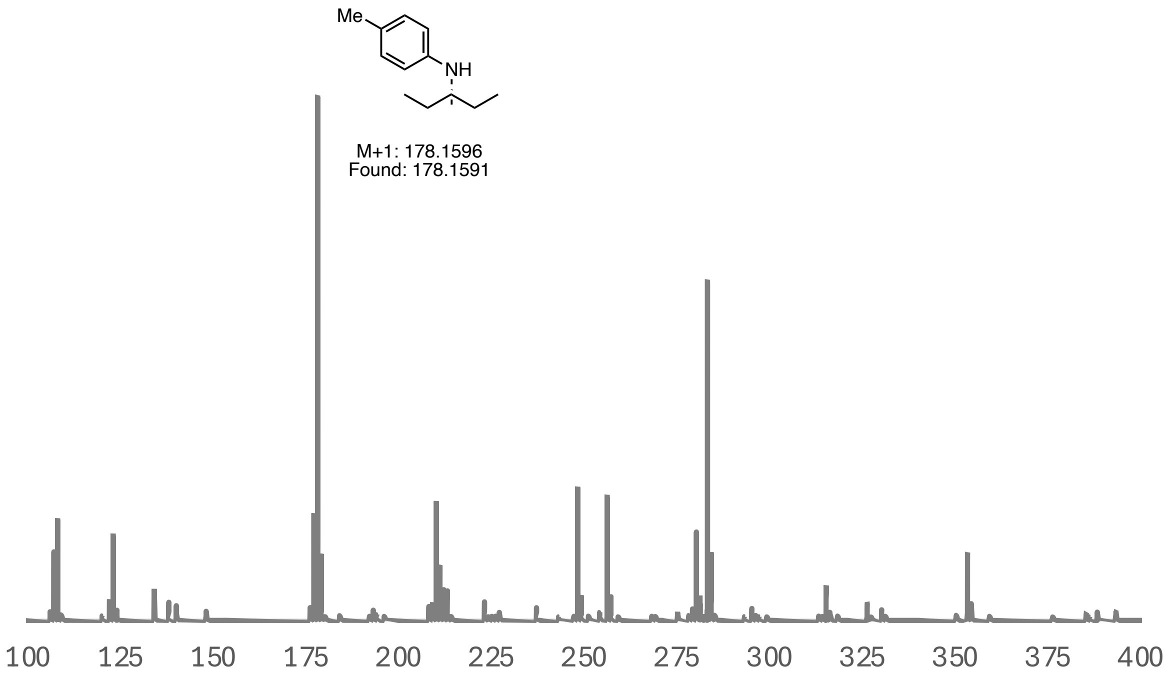

Figure S6. ESI-MS data obtained from the crude reaction mixture after thermolysis of **2** for 16 h show the presence of C–H aminated pentane.

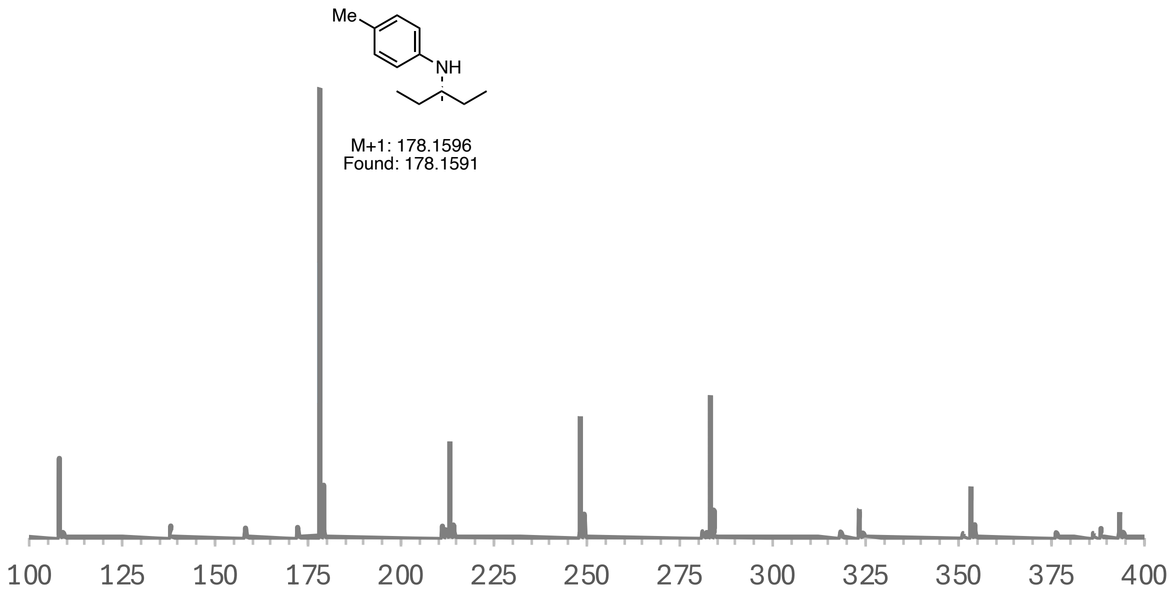

Figure S7. ESI-MS spectra of the crude reaction mixture after photolysis of **2** for 16 h show the presence of C–H aminated pentane molecules.

C.3. Thermolysis of 2 Under Ambient Atmosphere

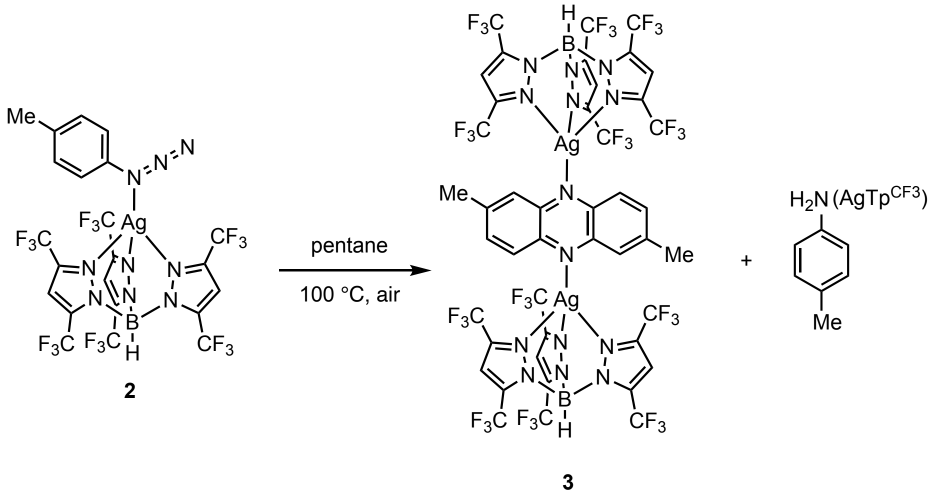

Under ambient atmosphere, a pentane solution (2.0 mL) of **2** (10 mg) was loaded into a 4-mL dram vial. The solution was then heated at 100 °C for 16 h. Upon concentrating the solution orange crystals of **3** were formed (see below for the crystallographic data). After this, the solvent was removed under reduced pressure, and the residue was taken up in $CDCl_3$. The crude reaction mixture was analyzed by ^1H NMR using trimethoxybenzene as the internal standard. The crude ^1H NMR showed 24% yield of **3** along with 39% Ag(Tp^{CF3})(*p*-toluidine)

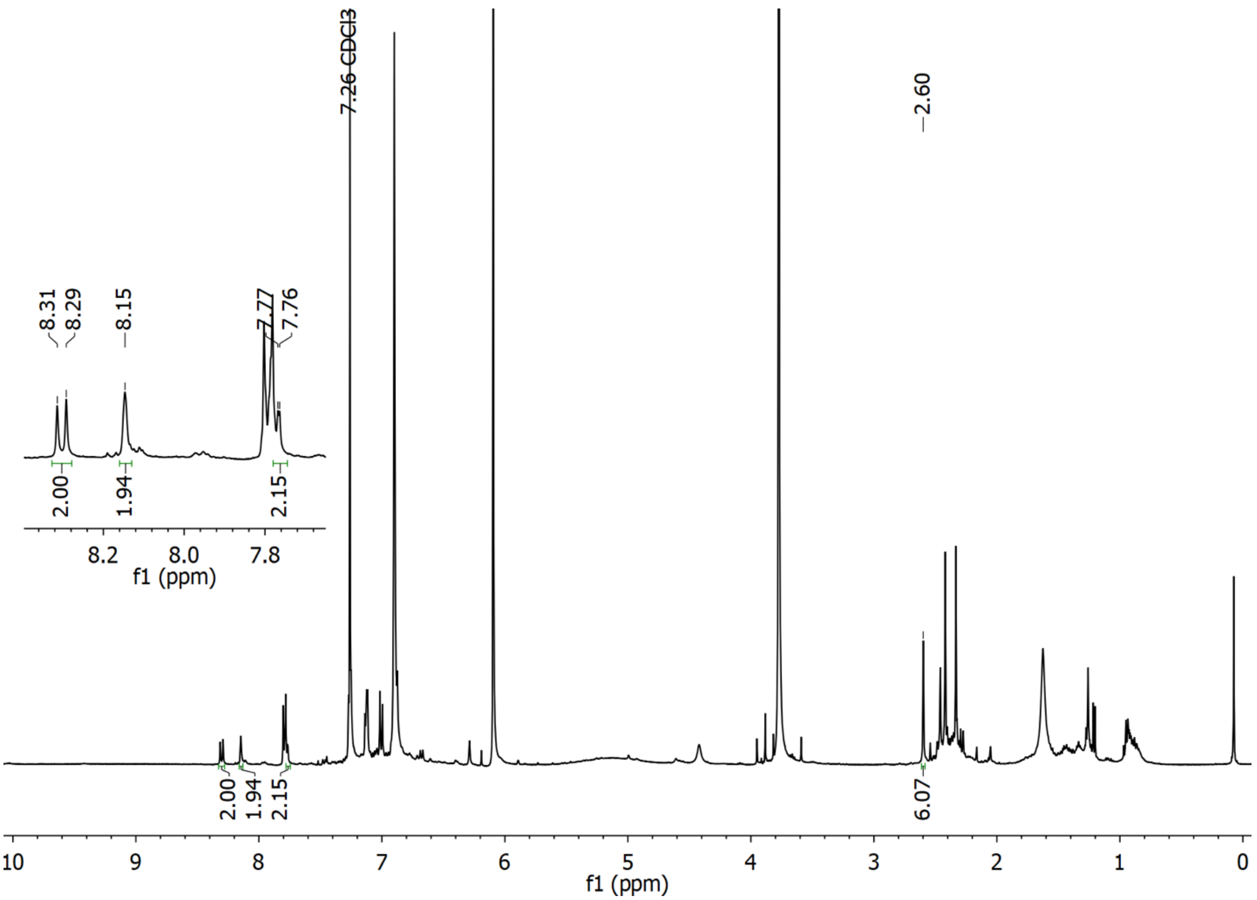

Figure S8. Crude ^{1}H NMR of **2** after thermolysis under ambient atmosphere. It shows the presence of **3** (inset) among other decomposition products. Yields were determined using trimethoxybenzene as an internal standard by integrating the resonance at 6.09 ppm. Note: Formation of **3** consumes two equivalents of azide precursor **2**.

C.4. Solid-State Photolysis of 2 Monitored by UV-vis Spectroscopy

In an N_2-filled glovebox, a thin film of **2** was prepared on the inner wall of a 2 mm EPR tube fitted with a J-Young cap by evaporation of a dilute dichloromethane solution of **2** under dynamic vacuum. The tube was sealed under vacuum, removed from the glovebox, and cooled to 77 K in liquid nitrogen-filled quartz finger dewar. The sample was irradiated (370 nm Kessil lamp) at 77 K. UV-vis spectra were obtained periodically during photolysis and revealed the growth of new absorption features at 315 nm and 400 nm. Upon thawing, the spectral features at 315 nm and 400 nm disappeared (Figure S9), thus demonstrating that these spectral features are attributable to a non-steady state intermediate (i.e., **4**).

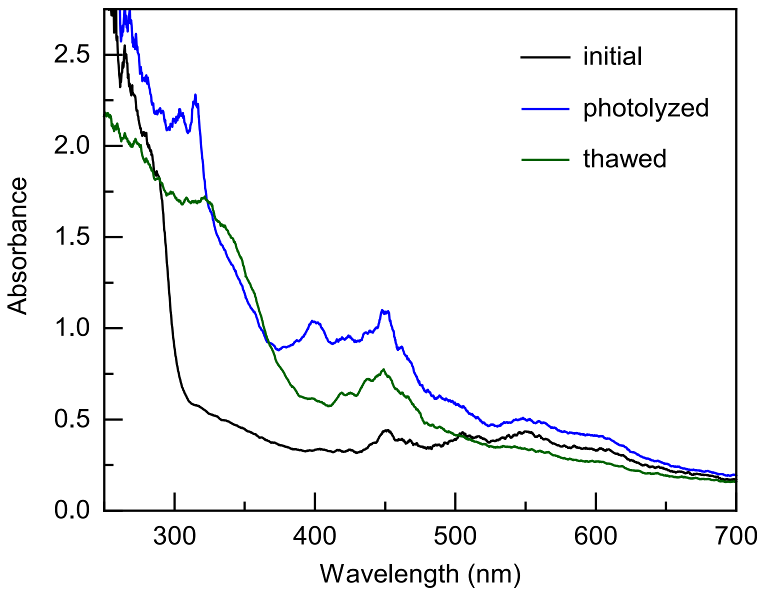

Figure S9. UV-vis spectra of **2** (black line), following photolysis of **2** at 77 K (i.e., **4**; blue line), and following a thaw-freeze cycle (green line). The band at ~450 nm is attributed to a secondary species arising from partial decomposition under the solid-state photolysis conditions and becomes more prominent upon thawing. As a limitation of solid-state cryogenic photolysis, we note that irradiation under these conditions can introduce local heating (e.g., from the Kessil lamp, which was positioned ~6 cm from the finger dewar to

allow sufficient light penetration through the bubbling liquid nitrogen), as well as temperature gradients arising from uneven cooling under vacuum. These factors make precise temperature control challenging and may contribute to minor decomposition pathways. We therefore do not assign this feature to the Ag–nitrene intermediate. In contrast, the transient bands at 315 and 400 nm, which are formed upon irradiation, are assigned to ^{3}[**4**] and are in good agreement with TD-DFT calculations (Figure S13).

C.5. SQUID Magnetometry

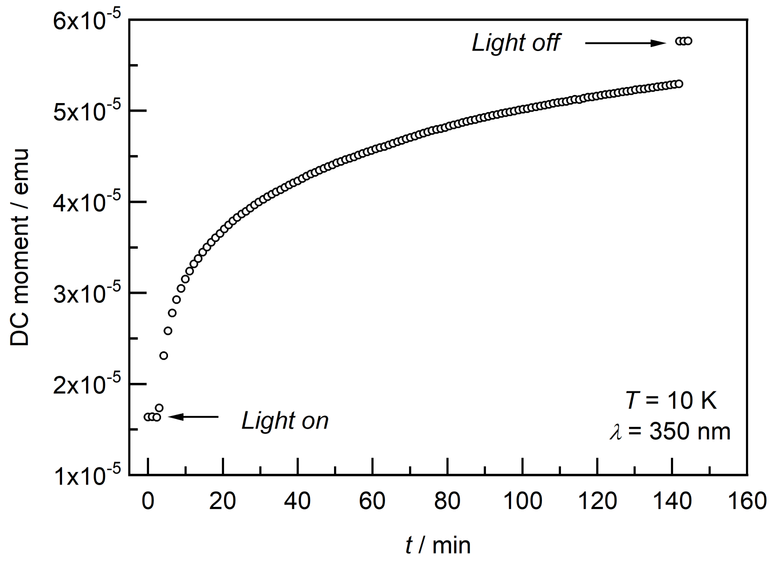

Figure S10. Plot of DC moment *vs.* time measured during irradiation of **2** at 350 nm at 10 K under applied magnetic field of 5000 Oe.

C.6. EPR Spectroscopy

In an N_2-filled glovebox, a dilute solution of *p*-tolylazide in 2-Me-THF was loaded in a 2 mm EPR tube and the tube was sealed. The EPR tube was transferred to a pre-cooled EPR probe (10 K), and an X-band spectrum was obtained. No signals were observed from 0 to 9000 G. The tube was then irradiated (370 nm) for 15 min at 10 K inside the probe. An X-band spectrum was obtained and displayed a signal centered at 6776 G (Figure S11). This observation is consistent with literature EPR data for related aryl nitrenes.[30] No comparable EPR signals were observed after photolysis of **2** in either pentane solution or in the solid state. The experimentally determined zero-field splitting parameter ($|D|$ = 6.4 cm^{-1}) is consistent with the absence of an X-band EPR signal for nitrene ^{3}[**4**] generated upon photolysis.

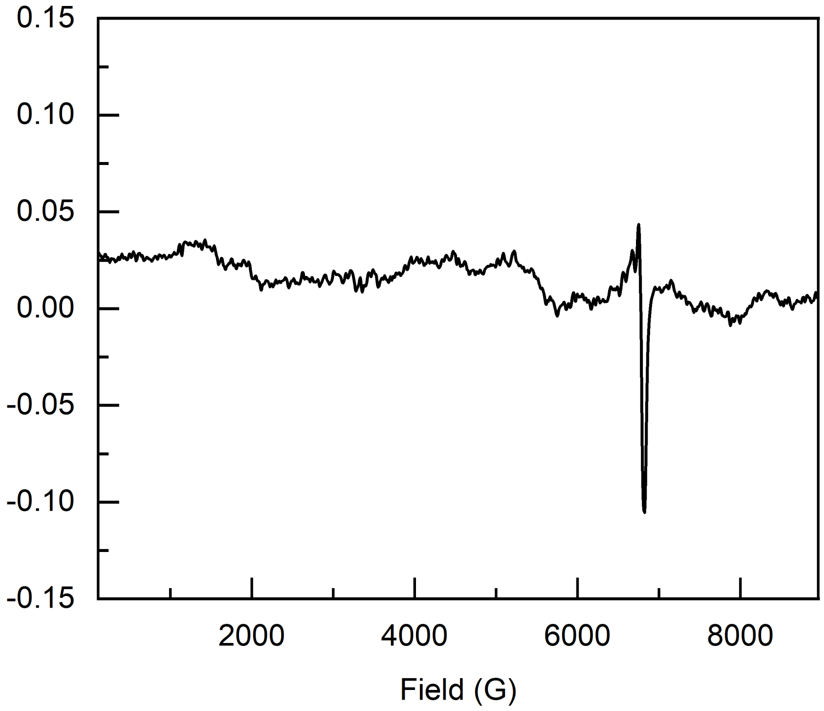

Figure S11. X-Band EPR spectrum of *p*-tolylazide obtained following photolysis (370 nm) at 10 K.

C.7. Computational Data

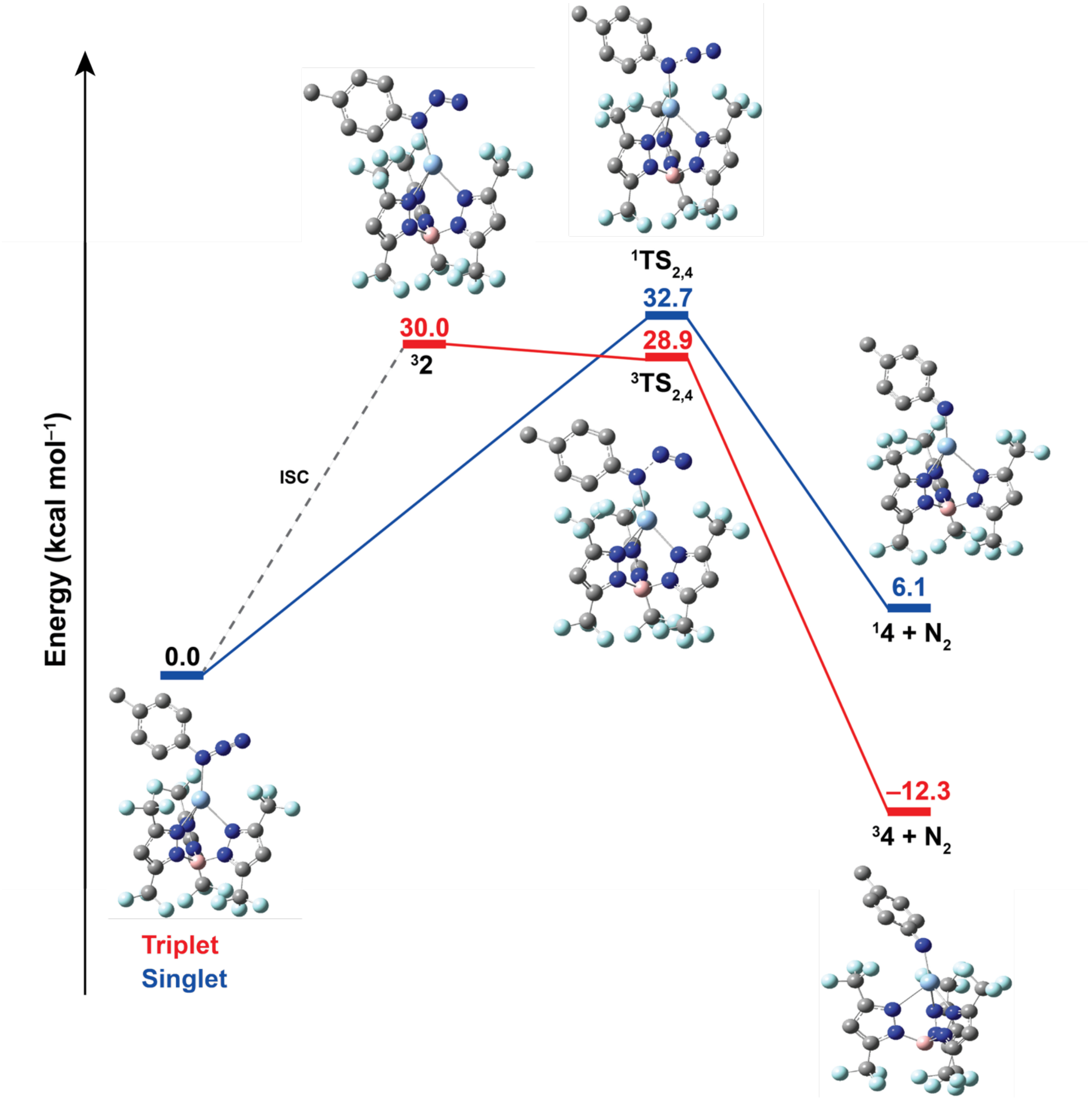

Figure S12. Gibbs Free Energy diagram of N_2 extrusion from **2** to form nitrene **4**. Energies computed at the PBE0-D3/BS2//PBE0-D3/BS1 level of theory.

Table S1. Comparison of crystallographic and DFT-calculated structural parameters for **4**

Parameters	Experimental	Calculated for ^{3}[4]	Calculated for ^{1}[4]
**Ag1–N1**	2.13(3) Å	2.12 Å	2.09 Å
**N1–C_{Ar}**	1.36(3) Å	1.32 Å	1.31 Å
**∠Ag–N1–C_{Ar}**	133(3)°	147°	124°

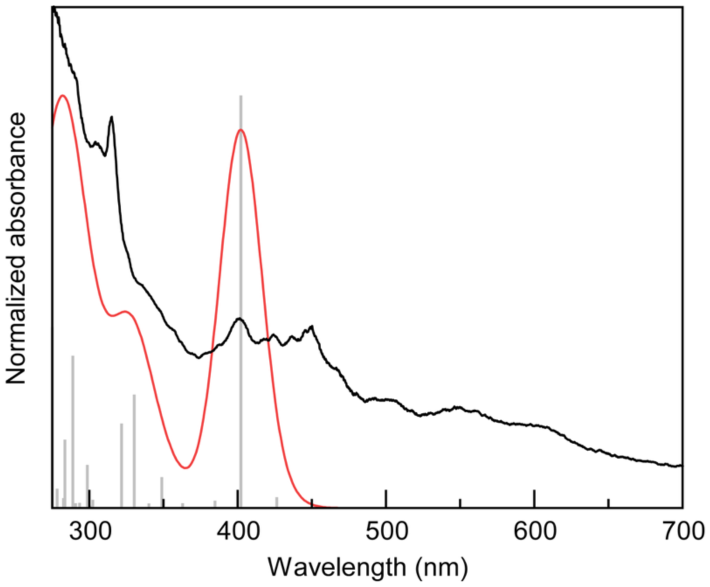

Figure S13. UV-vis spectrum of **4** overlayed with TD-DFT calculations: (–) experimental; (–) TD-DFT for ^{3}[**4**].

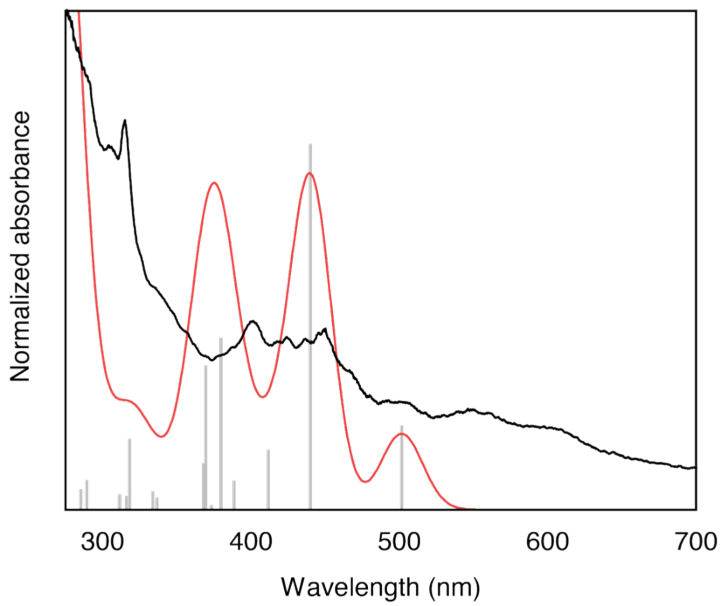

Figure S14. UV-vis spectrum of **4** overlayed with TD-DFT calculations: (–) experimental; (–) TD-DFT for ^{1}[**4**].

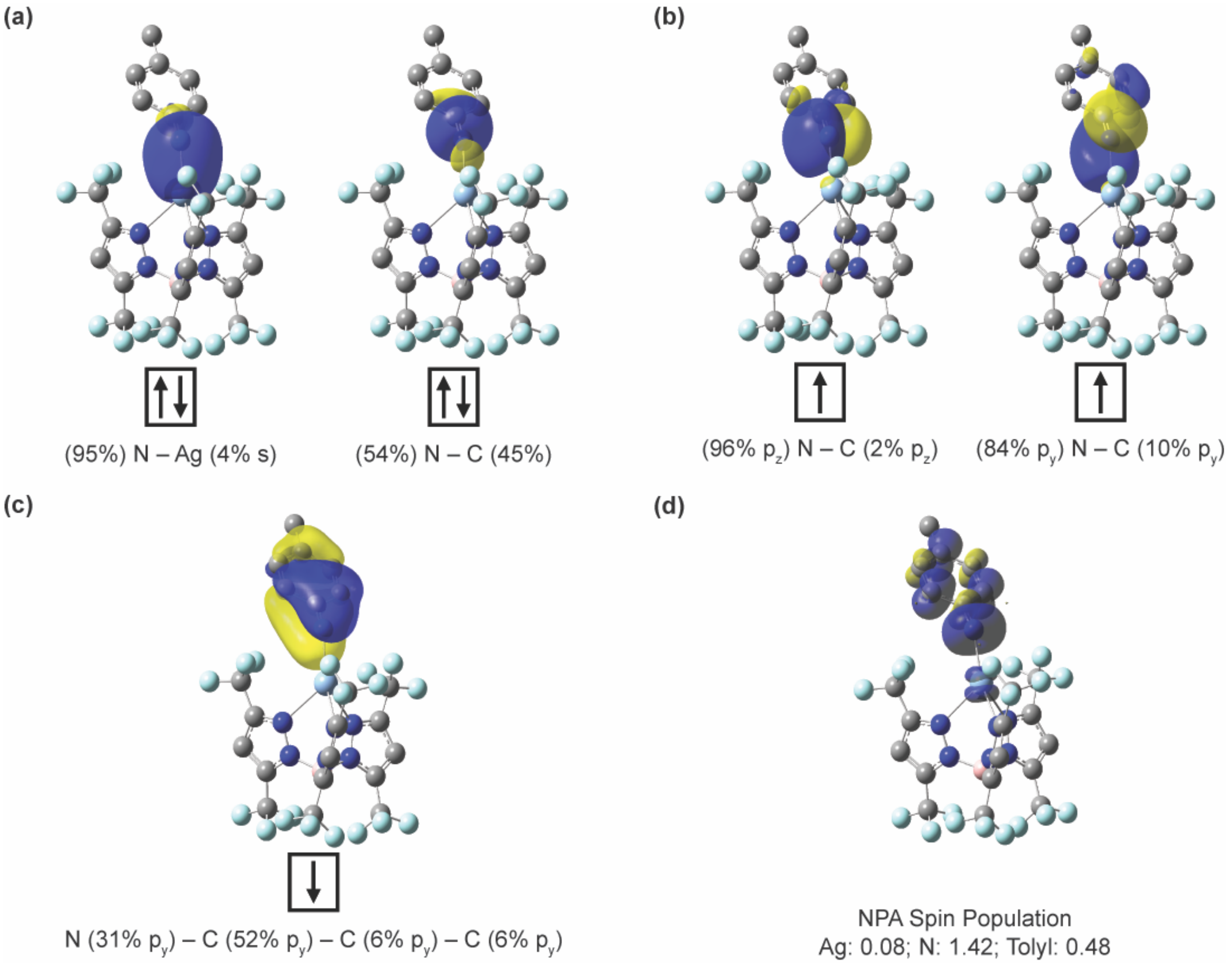

Figure S15. NLMOs of [3][**4**] from NBO analysis. a) Doubly occupied N_{sp} orbitals comprising the σ-interactions between Ag (left) and C_{Ar} (right). b) Singly occupied orbitals α orbitals on N_{pz} (left) and N_{py} (right). c) Lower energy β orbital contributing to N–C_{Ar} π-bonding. d) Spin-density plot and NPA spin populations.

(a) α-spin

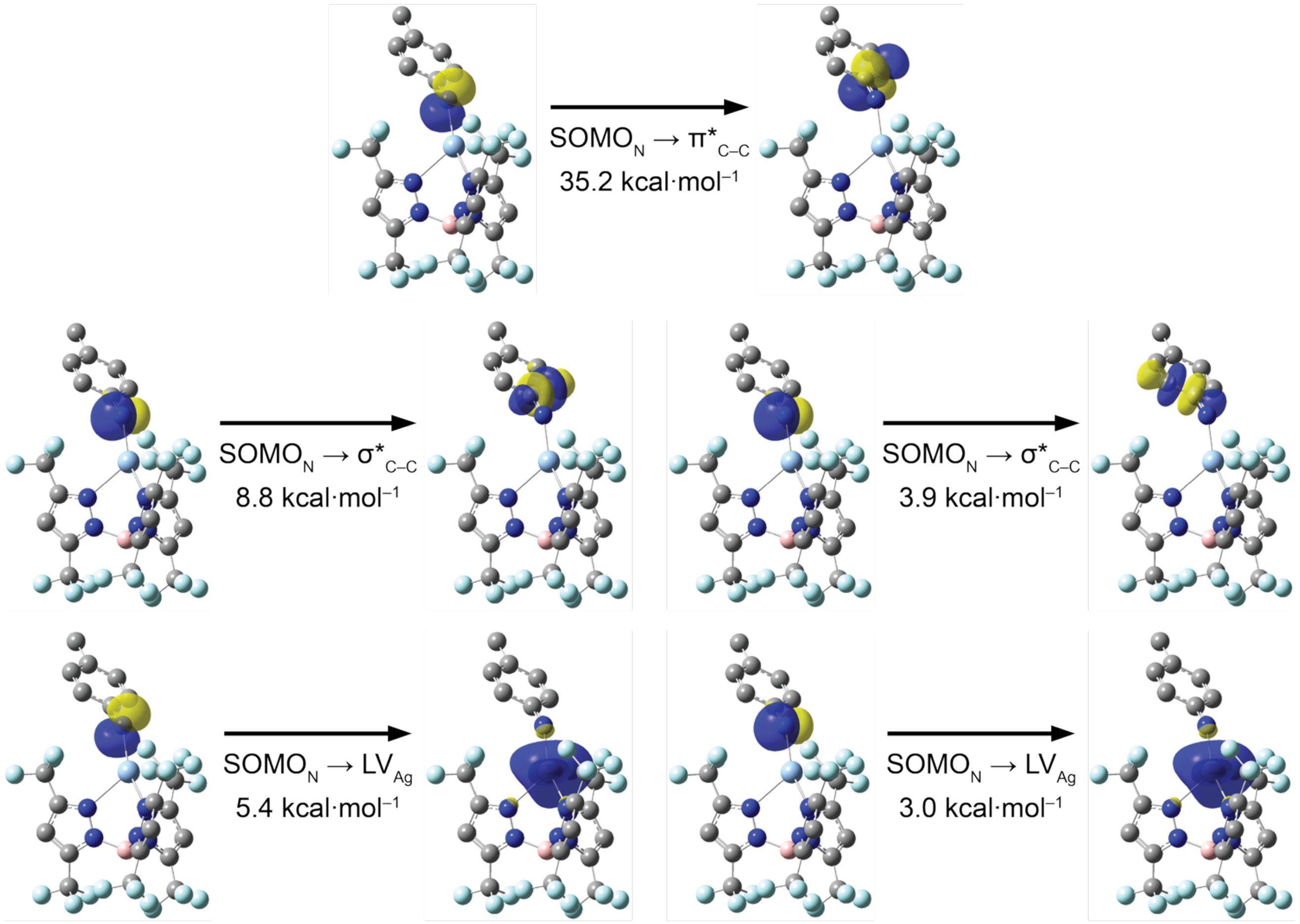

(b) β-spin

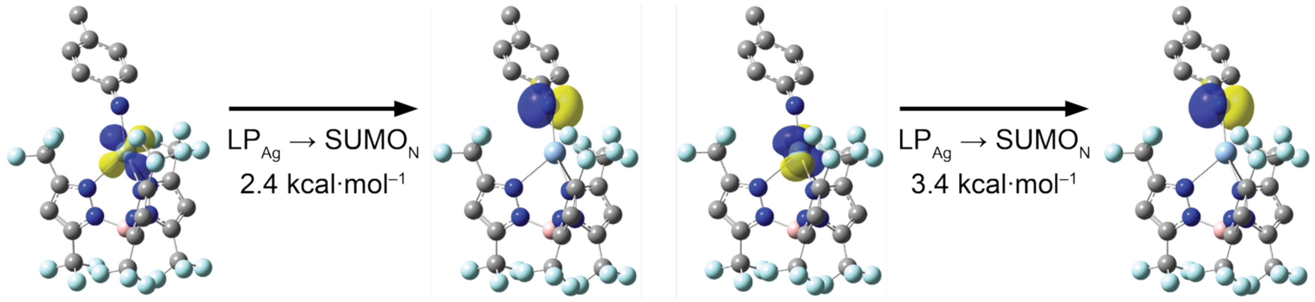

Figure S16. NBO 2nd-order perturbation theory donor-acceptor pairs: a) α spin pairs and b) β spin pairs.

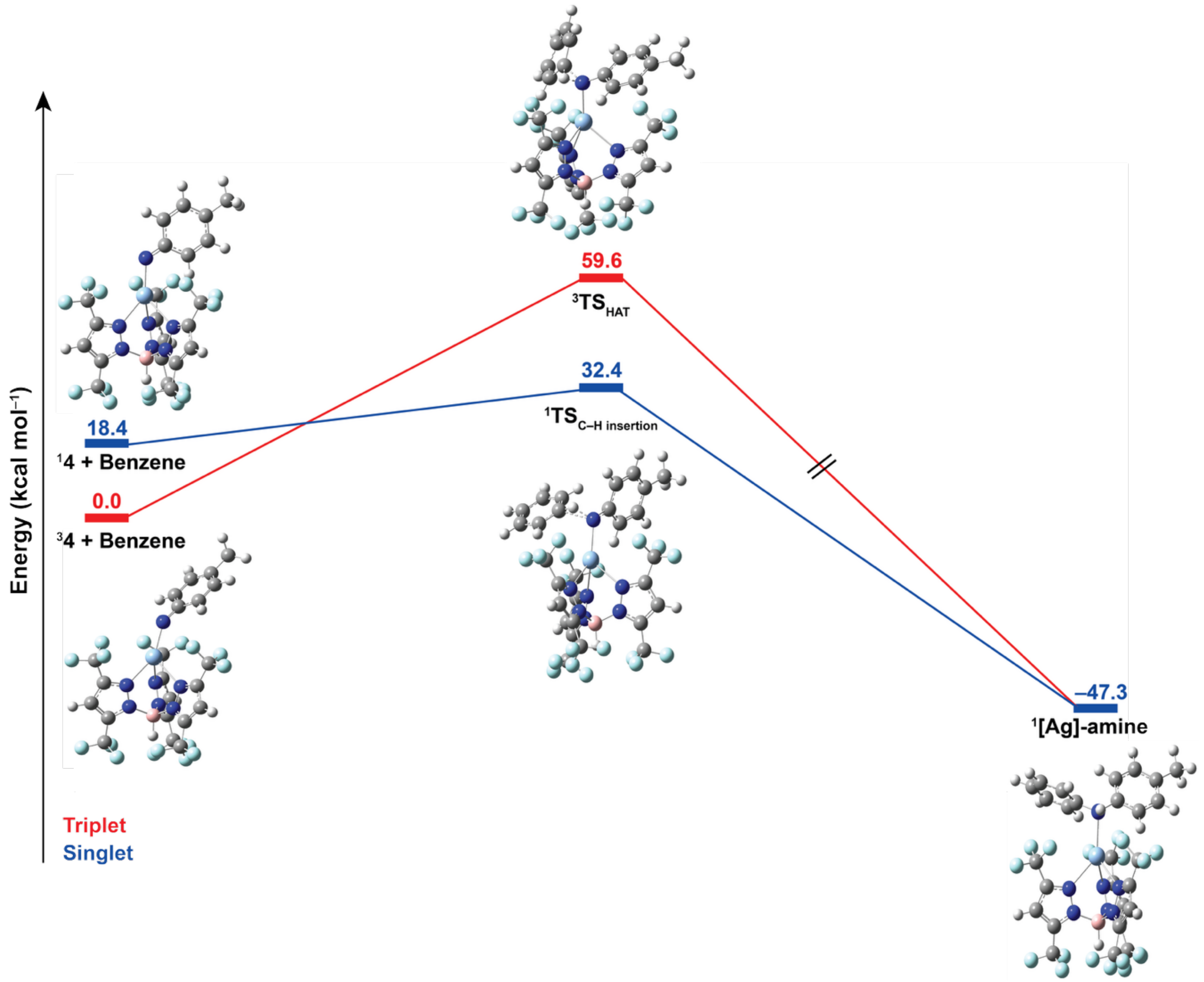

Figure S17. Computed reaction energy profile comparing (–) triplet and (–) singlet pathways for C–H amination of benzene. The triplet pathway proceeds via a high-energy HAT transition state, whereas the singlet pathway follows a lower-energy C–H insertion transition state, consistent with preferential reactivity on the singlet surface. Energies computed at the PBE0-D3/BS2//PBE0-D3/BS1 level of theory.

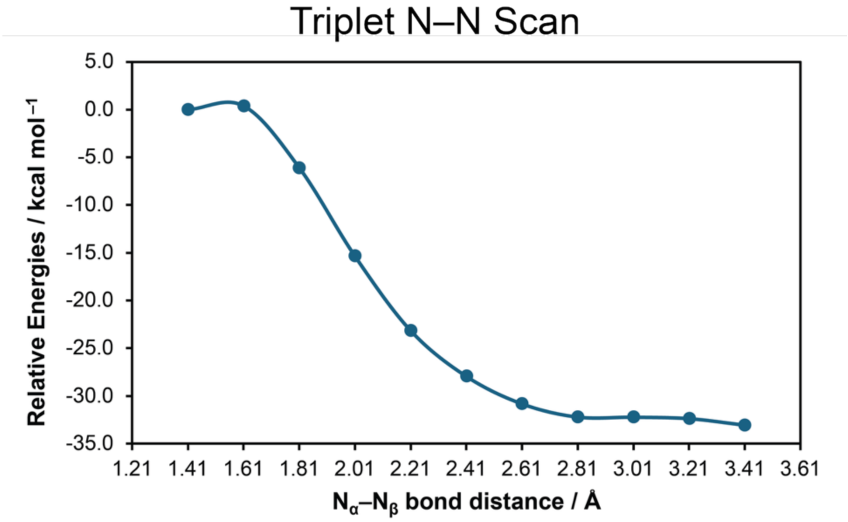

Figure S18. Relaxed potential energy surface scan of the N_α–N_β bond for the formation of ^{3}[**4**], showing a downhill profile consistent with barrierless N_2 extrusion. Single-point energies relative to the optimized structure of ^{3}[**2**] computed at the PBE0-D3/BS2//PBE0-D3/BS1 level of theory.

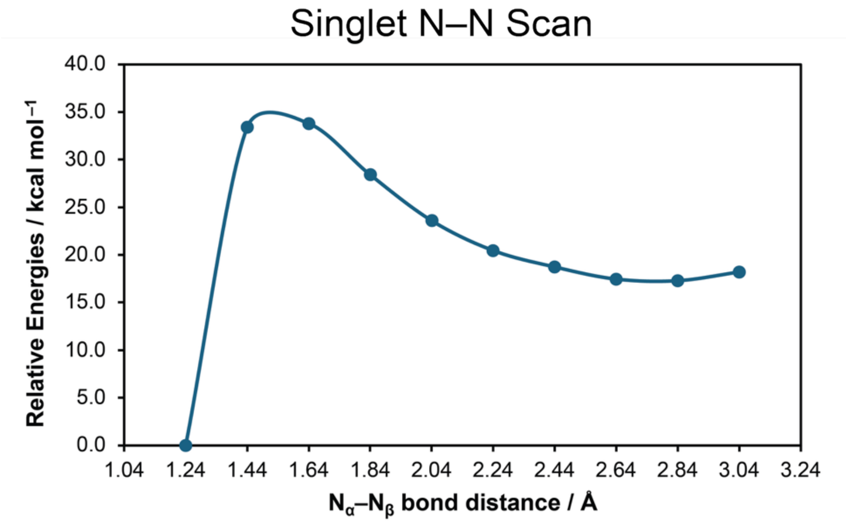

Figure S19. Relaxed potential energy surface scan of the N_α–N_β bond for the formation of ^{1}[**4**], showing a significant barrier to N_2 extrusion. Single-point energies relative to the optimized structure of ^{1}[**2**] computed at the PBE0-D3/BS2//PBE0-D3/BS1 level of theory.

Table S2. Optimized Coordinates of [1]Ag(pMePhN$_3$) ([1]**2**)

Ag	1.070629	0.647645	0.040262
F	-5.102487	1.023072	0.517124
F	-4.243266	-1.711719	-2.175363
F	1.580732	-1.238141	-4.531346
F	1.7992	-2.410686	3.939639
F	-3.173204	-3.183402	-1.018605
F	-4.507288	1.06243	-1.554569
F	1.15219	0.839638	-4.130704
F	2.370021	-0.779658	2.634256
F	2.304081	-0.302343	-2.716012
F	-0.502373	5.25988	0.931587
F	-3.216757	-3.325632	-3.174825
F	-3.585745	-2.351346	3.730419
N	-1.480724	-0.761302	-1.341848
F	2.281503	-2.790366	1.867771
F	-5.158471	2.879504	-0.582139
F	0.637748	3.669404	1.86268
F	-3.999605	-0.5878	2.551744
N	-2.091051	1.159075	0.179378
N	-0.31727	-0.224648	-1.713857
N	-1.411625	-1.070214	1.16461
N	-0.950255	1.765955	0.504641
F	-4.032667	-2.535539	1.627346
F	0.807227	4.008743	-0.253078
N	-0.078694	-1.107517	1.218521
N	3.244254	1.320374	0.13392
N	3.499572	2.516256	0.323471

C	-0.898329	-2.111026	3.06025
H	-0.965384	-2.628609	4.005705
C	-1.193249	3.071208	0.519948
C	0.243326	-1.725157	2.35032
C	-1.93606	-1.66759	2.260945
C	4.348535	0.422314	0.048055
C	-3.064552	2.08125	-0.010058
C	0.014366	-0.785685	-2.871325
C	-1.889871	-1.663813	-2.265026
C	-0.951327	-1.712601	-3.279398
H	-0.969067	-2.325543	-4.168435
C	1.677703	-1.929835	2.701459
C	-2.528421	3.339326	0.20099
H	-3.03204	4.29246	0.135157
C	1.265345	-0.368522	-3.568229
N	3.643583	3.623554	0.494908
C	5.675355	0.840803	0.129637
H	5.922794	1.892453	0.260856
C	6.418987	-1.462908	-0.116574
B	-2.156868	-0.377711	-0.003039
H	-3.293594	-0.72877	-0.01419
C	4.048214	-0.928556	-0.11587
H	3.011745	-1.250986	-0.172612
C	-0.066006	4.01165	0.775402
C	5.077915	-1.855009	-0.192357
H	4.830165	-2.908397	-0.308688
C	-3.401363	-1.784585	2.532567
C	-3.144071	-2.468713	-2.149958
C	-4.467068	1.747821	-0.406015

C	6.694068	-0.103273	0.046436
H	7.727946	0.230374	0.112929
C	7.52401	-2.475407	-0.223862
H	7.312712	-3.363598	0.383817
H	8.482637	-2.060211	0.10578
H	7.649704	-2.815162	-1.260966

PBE0-D3/BS1:

Electronic energy = -3302.39051717

Sum of electronic and zero-point energies = -3302.025124

Sum of electronic and thermal energy = -3301.977937

Sum of electronic and thermal enthalpy = -3301.976993

Sum of electronic and free energy = -3302.11828

Free Energy Correction = 0.27223717

PBE0-D3/BS2//PBE0-D3/BS1:

Electronic energy = -3305.004342

Table S3. Optimized Coordinates of ^{3}Ag(pMePhN$_3$) (^{3}**2**)

Ag	0.962043	1.109377	0.035649
F	-5.171117	0.327878	-0.201765
F	-3.522226	-2.871465	-1.810397
F	2.793687	-1.893175	-2.936058
F	1.662838	-0.802971	4.657141
F	-2.40429	-3.671279	-0.149012
F	-4.290949	-0.098479	-2.12304
F	1.655015	-0.868348	-4.447752
F	2.190662	0.498023	3.00961
F	2.306952	0.19904	-2.686522
F	-1.458681	5.321868	-0.706292
F	-2.1165	-4.476786	-2.133917
F	-3.635782	-1.531824	4.019827
N	-1.09491	-1.18157	-1.107135
F	2.431335	-1.631478	2.814793
F	-5.354544	1.756431	-1.808037
F	-0.303407	4.388056	0.868579
F	-4.10556	-0.283709	2.318863
N	-2.191626	0.945051	-0.298355
N	0.001458	-0.557596	-1.541326
N	-1.330699	-0.759213	1.366632
N	-1.221546	1.832031	-0.068177
F	-3.794315	-2.400422	2.050769
F	0.260899	4.100277	-1.189181
N	-0.018134	-0.58519	1.529598
N	3.151864	1.476277	0.153556
N	3.672991	2.778926	0.272521

C	-0.928472	-1.113043	3.522678
H	-1.045855	-1.336343	4.572768
C	-1.678887	3.019561	-0.450832
C	0.23669	-0.790811	2.818458
C	-1.909381	-1.078594	2.548352
C	4.102485	0.523411	0.095786
C	-3.268141	1.571113	-0.828417
C	0.574015	-1.364095	-2.42844
C	-1.219611	-2.385491	-1.715966
C	-0.158797	-2.549181	-2.584983
H	0.040896	-3.392105	-3.230648
C	1.633179	-0.681657	3.328387
C	-2.98267	2.91975	-0.944943
H	-3.622262	3.701519	-1.326887
C	1.833102	-0.972231	-3.126858
N	2.953717	3.705697	0.384754
C	5.504354	0.777079	0.105862
H	5.854088	1.80181	0.175462
C	5.944339	-1.608746	-0.072468
B	-1.999658	-0.564613	-0.0152
H	-3.060522	-1.103851	-0.021795
C	3.652572	-0.824792	0.014876
H	2.583213	-1.022348	0.033169
C	-0.791836	4.217103	-0.362883
C	4.558492	-1.856421	-0.068623
H	4.197625	-2.880428	-0.132402
C	-3.372273	-1.327129	2.724037
C	-2.330119	-3.348534	-1.446218
C	-4.52727	0.874685	-1.235021

C	6.389256	-0.274047	0.022154
H	7.458722	-0.073018	0.027676
C	6.920614	-2.735373	-0.201878
H	6.556291	-3.641237	0.295685
H	7.898318	-2.474925	0.217499
H	7.073957	-2.987708	-1.261357

PBE0-D3/BS1:

Electronic energy = -3302.34306188

Sum of electronic and zero-point energies = -3301.933399

Sum of electronic and thermal energy = -3301.977937

Sum of electronic and thermal enthalpy = -3301.932454

Sum of electronic and free energy = -3302.074833

Free Energy Correction = 0.26822888

PBE0-D3/BS2//PBE0-D3/BS1:

Electronic energy = -3304.952486

Table S4. Optimized Coordinates of [1]Ag(pMePhN) ([1]**4**)

Ag	1.192468	-0.888564	0.19018
F	-5.025786	-0.840473	-0.56896
F	-4.070297	1.866375	2.098369
F	1.497318	1.006856	4.733507
F	2.113383	2.085804	-3.944021
F	-2.963922	3.272749	0.895662
F	-4.475334	-0.879091	1.514761
F	1.642517	-0.812226	3.56736
F	2.605398	0.477878	-2.582549
F	2.502109	1.015144	2.821219
F	-0.73353	-5.341062	-0.839828
F	-3.002564	3.484944	3.045863
F	-3.255411	2.274423	-3.87545
N	-1.337627	0.810131	1.307402
F	2.572266	2.510883	-1.875869
F	-5.204956	-2.673216	0.555201
F	0.51398	-3.869523	-1.822637
F	-3.791418	0.587963	-2.635023
N	-2.025624	-1.139532	-0.145354
N	-0.19068	0.257966	1.700558
N	-1.20975	1.000697	-1.199742
N	-0.911205	-1.81285	-0.434856
F	-3.738203	2.572114	-1.793133
F	0.699763	-4.189244	0.297761
N	0.122842	0.962493	-1.215897

N	3.167673	-1.570568	0.216416
C	-0.599766	1.915667	-3.128946
H	-0.61564	2.388885	-4.099545
C	-1.233589	-3.101675	-0.45684
C	0.501817	1.506784	-2.367794
C	-1.678348	1.567488	-2.337958
C	4.217424	-0.795931	0.11791
C	-3.058148	-2.000274	0.017878
C	0.161285	0.856127	2.833733
C	-1.718146	1.762178	2.191258
C	-0.775579	1.827739	3.201375
H	-0.777226	2.47318	4.067216
C	1.947744	1.642209	-2.695777
C	-2.591598	-3.287463	-0.176511
H	-3.151146	-4.209616	-0.124494
C	1.451079	0.506811	3.495601
C	5.52368	-1.403137	0.036134
H	5.55593	-2.488845	0.085497
C	6.562753	0.764847	-0.172561
B	-2.015156	0.400277	-0.023523
H	-3.131376	0.812351	-0.046358
C	4.162047	0.646482	0.067068
H	3.184428	1.114513	0.146475
C	-0.181407	-4.129467	-0.710853
C	5.303456	1.396106	-0.069189
H	5.248886	2.481918	-0.111624
C	-3.12752	1.749583	-2.650521

C	-2.952271	2.593266	2.048482
C	-4.447459	-1.584308	0.377068
C	6.658204	-0.641473	-0.118286
H	7.638224	-1.108005	-0.1936
C	7.789337	1.595286	-0.36402
H	7.852958	1.918576	-1.413662
H	8.703327	1.04087	-0.130193
H	7.754216	2.50516	0.24615

PBE0-D3/BS1:

Electronic energy = -3192.95618624

Sum of electronic and zero-point energies = -3192.601898

Sum of electronic and thermal energy = -3192.556685

Sum of electronic and thermal enthalpy = -3192.555741

Sum of electronic and free energy = -3192.692070

Free Energy Correction = 0.26411624

PBE0-D3/BS2//PBE0-D3/BS1:

Electronic Energy = -3195.527411

Table S5. Optimized Coordinates of ^{3}Ag(pMePhN) (^{3}**4**)

Ag	1.095947	0.907718	-0.041477
F	-5.105309	0.728837	-0.174573
F	-3.772917	-2.55495	-1.8331
F	2.035302	-2.035816	-4.017032
F	1.606199	-0.725181	4.691124
F	-2.794775	-3.517153	-0.170253
F	-4.320302	0.193494	-2.109907
F	2.113387	-0.056268	-3.136522
F	2.083227	0.689849	3.123616
F	2.816483	-1.759063	-2.024583
F	-0.879642	5.2822	-1.133191
F	-2.564019	-4.311343	-2.166797
F	-3.709895	-1.338717	4.042364
N	-1.183414	-1.17308	-1.067219
F	2.400481	-1.41173	2.800629
F	-5.201395	2.14246	-1.801898
F	-0.178903	4.573063	0.78344
F	-4.152951	-0.0816	2.341217
N	-2.090258	1.073334	-0.342299
N	-0.005563	-0.690808	-1.462962
N	-1.381732	-0.624574	1.390886
N	-1.036038	1.870694	-0.158681
F	-3.884635	-2.203846	2.073107
F	0.768796	3.88126	-1.021102
N	-0.070202	-0.463367	1.565767
N	3.200371	1.160138	-0.061947
C	-1.000538	-0.951257	3.556249

H	-1.129588	-1.158018	4.608328
C	-1.388622	3.08347	-0.571158
C	0.174426	-0.653856	2.857144
C	-1.973369	-0.919487	2.573536
C	4.382029	0.583212	-0.036376
C	-3.115272	1.782082	-0.871774
C	0.499549	-1.561511	-2.328271
C	-1.429777	-2.354348	-1.683873
C	-0.364123	-2.647551	-2.514436
H	-0.242407	-3.509303	-3.154053
C	1.567933	-0.531116	3.371478
C	-2.707276	3.093348	-1.036091
H	-3.27825	3.919356	-1.43338
C	1.866671	-1.350361	-2.883479
C	5.084826	0.307777	-1.251478
H	4.605198	0.554177	-2.193767
C	6.931131	-0.631142	0.022717
B	-2.033119	-0.436998	-0.002613
H	-3.135641	-0.883691	0.009229
C	4.98867	0.2261	1.209177
H	4.439415	0.417497	2.125527
C	-0.416465	4.211997	-0.482499
C	6.232167	-0.367284	1.215953
H	6.684125	-0.643569	2.166935
C	-3.441462	-1.139808	2.74652
C	-2.654828	-3.180034	-1.457654
C	-4.442791	1.197608	-1.234186
C	6.326739	-0.28603	-1.201377
H	6.851947	-0.500363	-2.130451

C	8.297563	-1.24547	0.05488
H	9.072991	-0.466643	0.094139
H	8.488846	-1.848567	-0.839879
H	8.432045	-1.882513	0.936196

PBE0-D3/BS1:

Electronic energy = -3192.98584955

Sum of electronic and zero-point energies = -3192.632383

Sum of electronic and thermal energy = -3192.587026

Sum of electronic and thermal enthalpy = -3192.586082

Sum of electronic and free energy = -3192.723494

Free Energy Correction = 0.26235555

PBE0-D3/BS2//PBE0-D3/BS1:

Electronic Energy = -3195.55494951

Table S6. Optimized Coordinates of (^{1}**TS**$_{2,4}$)

Ag	-1.112842	0.658193	0.298108
F	5.124167	0.830728	0.40362
F	4.015027	-2.760304	1.253968
F	-1.70598	-3.004396	3.631001
F	-1.549458	0.170404	-4.728167
F	3.063451	-3.397846	-0.572501
F	4.323991	-0.122601	2.163896
F	-2.002124	-0.940015	3.04109
F	-2.195997	0.913153	-2.800203
F	-2.594999	-2.536387	1.722455
F	0.685956	4.957539	1.989881
F	2.91449	-4.615579	1.206844
F	3.803357	-0.193526	-4.210687
N	1.324504	-1.412612	0.798665
F	-2.207546	-1.202376	-3.19403
F	5.110019	1.887132	2.284988
F	-0.088074	4.442015	0.037479
F	4.156318	0.641071	-2.248701
N	2.09034	0.986652	0.546434
N	0.128519	-1.09825	1.297473
N	1.453183	-0.356988	-1.48909
N	1.000577	1.752306	0.483464
F	4.069693	-1.497236	-2.511245
F	-0.887481	3.483464	1.789281
N	0.129286	-0.278799	-1.625809
N	-3.187368	1.303399	0.54472
N	-3.248522	2.768206	-0.377729

C	1.072557	-0.175995	-3.670371
H	1.203457	-0.105811	-4.740073
C	1.281399	2.877842	1.131313
C	-0.113586	-0.167468	-2.92755
C	2.05246	-0.297159	-2.702456
C	-4.220396	0.474009	0.20102
C	3.066425	1.626169	1.233586
C	-0.295869	-2.154302	1.982232
C	1.660816	-2.672654	1.164002
C	0.639384	-3.194691	1.935514
H	0.590332	-4.171274	2.394256
C	-1.517363	-0.070224	-3.416505
C	2.587211	2.860867	1.632533
H	3.106279	3.621766	2.196318
C	-1.652127	-2.152947	2.602594
N	-3.068731	3.862592	-0.330466
C	-5.536945	0.767487	0.636717
H	-5.713332	1.717133	1.137034
C	-6.310035	-1.379605	-0.17454
B	2.11432	-0.423495	-0.09165
H	3.240319	-0.794033	-0.196175
C	-3.987932	-0.755987	-0.453193
H	-2.986952	-0.975516	-0.815476
C	0.247432	3.947134	1.236318
C	-5.01272	-1.673282	-0.613525
H	-4.814	-2.626615	-1.098444
C	3.53154	-0.341343	-2.908644
C	2.92776	-3.354486	0.758244
C	4.413775	1.04382	1.513678

C	-6.558454	-0.139304	0.444264
H	-7.565509	0.091643	0.786648
C	-7.418323	-2.374963	-0.336075
H	-7.609337	-2.889718	0.616189
H	-7.172345	-3.138319	-1.08129
H	-8.354756	-1.887066	-0.63112

PBE0-D3/BS1:

Electronic energy = -3302.33654745

Sum of electronic and zero-point energies = -3301.975069

Sum of electronic and thermal energy = -3301.927555

Sum of electronic and thermal enthalpy = -3301.926611

Sum of electronic and free energy = -3302.067912

Free Energy Correction = 0.2686354500

PBE0-D3/BS2//PBE0-D3/BS1:

Electronic energy = -3304.94857266

Table S7. Optimized Coordinates of (^{3}**TS$_{2,4}$**)

Ag	0.993394	1.026157	-0.015453
F	-5.170617	0.479042	-0.154752
F	-3.681667	-2.782166	-1.730719
F	2.71208	-2.106598	-2.779967
F	1.669373	-0.762797	4.654917
F	-2.591024	-3.619562	-0.069764
F	-4.346864	-0.028717	-2.081308
F	1.632905	-1.212968	-4.413575
F	2.182918	0.547148	3.010344
F	2.25581	0.005655	-2.740059
F	-1.258898	5.296454	-0.951335
F	-2.360884	-4.458884	-2.048093
F	-3.653379	-1.366523	4.086712
N	-1.164105	-1.208669	-1.06621
F	2.410743	-1.581865	2.796594
F	-5.332946	1.872662	-1.793768
F	-0.202024	4.43091	0.726811
F	-4.114277	-0.142282	2.366158
N	-2.175146	0.98215	-0.316453
N	-0.037055	-0.651157	-1.512833
N	-1.359464	-0.698097	1.395467
N	-1.166949	1.836688	-0.133479
F	-3.851433	-2.270055	2.137043
F	0.426154	3.97857	-1.282363
N	-0.042387	-0.547934	1.543792
N	3.158934	1.396185	0.097259
N	3.732133	2.860509	0.238362

C	-0.944084	-1.01312	3.555293
H	-1.056164	-1.211172	4.611002
C	-1.5834	3.027513	-0.550666
C	0.220912	-0.73064	2.833969
C	-1.933309	-0.979568	2.589283
C	4.142524	0.489581	0.064994
C	-3.235442	1.632372	-0.850712
C	0.486949	-1.498192	-2.391803
C	-1.35577	-2.412014	-1.658971
C	-0.309563	-2.643493	-2.530153
H	-0.158486	-3.504212	-3.165421
C	1.624352	-0.632327	3.327308
C	-2.898734	2.96356	-1.019395
H	-3.513222	3.756519	-1.419043
C	1.772978	-1.193137	-3.084357
N	3.007373	3.736986	0.396849
C	5.538395	0.784118	0.091135
H	5.858181	1.818602	0.15229
C	6.060106	-1.588502	-0.050695
B	-2.034712	-0.52417	0.013818
H	-3.114257	-1.024217	0.033623
C	3.741399	-0.879784	-0.006135
H	2.679663	-1.113766	-0.001591
C	-0.651888	4.19297	-0.508311
C	4.682607	-1.88038	-0.064302
H	4.355277	-2.91618	-0.12219
C	-3.399308	-1.193151	2.784459
C	-2.511924	-3.314372	-1.370737
C	-4.527986	0.974545	-1.214262

C	6.457681	-0.239594	0.033154
H	7.519601	-0.002228	0.051364
C	7.072772	-2.686445	-0.148883
H	6.760801	-3.570229	0.419802
H	8.054675	-2.366686	0.215647
H	7.195296	-3.00347	-1.194718

PBE0-D3/BS1:

Electronic energy = -3302.34114117

Sum of electronic and zero-point energies = -3301.980683

Sum of electronic and thermal energy = -3301.93315

Sum of electronic and thermal enthalpy = -3301.932206

Sum of electronic and free energy = -3302.075626

Free Energy Correction = 0.265515170

PBE0-D3/BS2//PBE0-D3/BS1:

Electronic Energy = -3304.9514895

Table S8. Optimized Coordinates of (^{1}**TS**$_{\text{C–H insertion}}$)

Ag	0.920445	-0.199783	-0.565248
F	-4.708659	0.756551	-1.796702
F	-4.126079	-1.994013	1.730552
F	1.986374	0.21107	3.321381
F	1.388046	2.546638	-2.346457
F	-4.293519	-0.213064	2.934237
F	-5.286733	-1.016641	-0.714338
F	1.607809	-1.712748	4.234095
F	1.843799	3.671064	-0.564745
F	2.243188	-1.587203	2.170212
F	-0.571155	-3.11568	-4.304376
F	-3.742733	-2.08433	3.856032
F	-3.687181	4.363393	1.162672
N	-1.62673	-0.420905	1.468468
F	0.698825	4.590509	-2.140974
F	-5.306627	-1.019583	-2.872061
F	0.909157	-2.067576	-3.120919
F	-4.623868	2.626268	0.291469
N	-2.27155	-0.746104	-0.955254
N	-0.297712	-0.351092	1.552538
N	-1.786546	1.567822	-0.073902
N	-1.111316	-1.309959	-1.294121
F	-3.546109	2.445409	2.149642
F	0.041307	-3.895662	-2.386868
N	-0.625874	1.671067	-0.722153

N	2.984349	-0.138842	-1.248547
C	-1.391078	3.743056	-0.280916
H	-1.479911	4.81855	-0.233848
C	-1.331149	-1.985778	-2.418201
C	-0.37534	2.968671	-0.856292
C	-2.273112	2.799816	0.210396
C	3.874144	0.730218	-0.693838
C	-3.227771	-1.064424	-1.859941
C	0.043516	-0.948697	2.690383
C	-2.130594	-1.064919	2.54751
C	-1.081155	-1.425634	3.372415
H	-1.129608	-1.948668	4.315966
C	0.891814	3.439225	-1.486503
C	-2.663517	-1.87182	-2.829317
H	-3.14443	-2.303032	-3.694763
C	1.473586	-1.014562	3.10328
C	3.621613	1.3793	0.540901
H	2.745352	1.082097	1.112326
C	5.617574	2.725996	0.285359
B	-2.392	0.188233	0.271124
H	-3.539596	0.318235	0.55876
C	5.054331	1.081144	-1.400391
H	5.25468	0.572695	-2.341106
C	-0.236469	-2.76446	-3.061251
C	5.904336	2.058245	-0.917925
H	6.799025	2.328309	-1.475912
C	-3.544827	3.048567	0.954228

C	-3.585234	-1.33164	2.760159
C	-4.639895	-0.579915	-1.797741
C	4.466788	2.369086	1.005555
H	4.246562	2.873554	1.944307
C	6.514168	3.820091	0.779646
H	6.501352	3.88891	1.872991
H	6.177294	4.790893	0.388688
H	7.548818	3.677	0.449138
H	3.556885	-1.322878	-1.412065
C	3.727163	-2.132098	-0.533588
C	4.81035	-2.006215	0.316923
C	2.895083	-3.243562	-0.542855
C	5.117826	-3.073584	1.160269
H	5.401737	-1.091219	0.334582
C	3.210057	-4.300494	0.310326
H	2.037017	-3.298871	-1.204455
C	4.314489	-4.213656	1.158369
H	5.97206	-3.006575	1.830508
H	2.583846	-5.190162	0.315877
H	4.542909	-5.03547	1.833422

PBE0-D3/BS1:

Electronic energy = -3424.92258033

Sum of electronic and zero-point energies = -3424.469638

Sum of electronic and thermal energy = -3424.41916

Sum of electronic and thermal enthalpy = -3424.418215

Sum of electronic and free energy = -3424.565627

Free Energy Correction = 0.35695333

PBE0-D3/BS2//PBE0-D3/BS1:

Electronic Energy = -3427.578222

Table S9. Optimized Coordinates of (^{3}**TS$_{HAT}$**)

Ag	-0.957987	-0.54222	-0.109954
F	5.108798	-0.927911	-1.340903
F	4.203621	2.918858	-0.576099
F	-1.153737	2.958523	-3.384003
F	-0.533326	-1.213474	4.992886
F	3.354518	2.843828	1.404614
F	4.124282	0.57817	-2.528561
F	-2.214904	1.751661	-1.950749
F	-1.412408	-1.878409	3.133891
F	-2.013489	3.890925	-1.645268
F	0.167413	-4.23119	-3.228315
F	3.138915	4.627356	0.20122
F	4.640503	-1.267166	3.499256
N	1.511486	1.489167	-0.454998
F	-1.506607	0.200672	3.676619
F	4.68567	-1.308473	-3.42193
F	-0.410035	-4.172773	-1.144347
F	4.563464	-1.51255	1.353507
N	2.083479	-0.904924	-0.973045
N	0.306672	1.354088	-1.014957
N	1.910021	-0.15487	1.433101
N	0.951174	-1.610704	-0.951265
F	4.780084	0.455643	2.207126
F	-1.231029	-2.738601	-2.524535
N	0.627364	-0.180213	1.792236

N	-3.049246	-0.478812	0.714099
C	1.876922	-0.948014	3.506706
H	2.172831	-1.337325	4.469656
C	1.027082	-2.490768	-1.945428
C	0.59217	-0.656948	3.0332
C	2.691236	-0.608992	2.442549
C	-3.670147	0.775432	0.562221
C	2.87683	-1.334036	-1.981433
C	-0.109076	2.578567	-1.324088
C	1.859773	2.79632	-0.405645
C	0.838147	3.544024	-0.958702
H	0.795696	4.61649	-1.081477
C	-0.710952	-0.879825	3.715402
C	2.23384	-2.366388	-2.640421
H	2.588071	-2.933702	-3.488259
C	-1.381992	2.788294	-2.073731
C	-4.753255	0.989588	-0.299773
H	-5.173994	0.157577	-0.85556
C	-4.665353	3.378402	0.154497
B	2.334946	0.266223	0.008747
H	3.493674	0.539881	0.007646
C	-3.086573	1.871008	1.218942
H	-2.23851	1.699126	1.877225
C	-0.112844	-3.413135	-2.212129
C	-3.588901	3.145914	1.022721
H	-3.125104	3.986261	1.536631
C	4.178793	-0.725586	2.365826

C	3.151617	3.290138	0.15855
C	4.209262	-0.739319	-2.30853
C	-5.236553	2.279749	-0.490838
H	-6.062989	2.437895	-1.181874
C	-5.138262	4.776448	-0.11872
H	-4.460762	5.276132	-0.825143
H	-5.15985	5.382201	0.79525
H	-6.141468	4.78604	-0.558965
H	-3.119938	-0.999243	1.792131
C	-3.820248	-1.722143	0.880602
C	-5.285766	-1.748473	0.731337
C	-3.065252	-2.884837	0.367493
C	-5.840453	-2.625746	-0.152879
H	-5.896134	-1.063769	1.314479
C	-3.661876	-3.712089	-0.540868
H	-2.033418	-3.028235	0.681334
C	-5.039906	-3.578861	-0.848503
H	-6.919571	-2.634567	-0.298754
H	-3.083336	-4.513845	-0.993507
H	-5.506363	-4.261919	-1.552704

PBE0-D3/BS1:

Electronic energy = -3424.882598

Sum of electronic and zero-point energies = -3424.430966

Sum of electronic and thermal energy = -3424.380669

Sum of electronic and thermal enthalpy = -3424.379724

Sum of electronic and free energy = -3424.524954

Free Energy Correction = 0.35764417

PBE0-D3/BS2//PBE0-D3/BS1:

Electronic Energy = -3427.535512

Table S10. Optimized Coordinates of (³**[Ag]-amine**)

Ag	-0.947991	0.243104	-0.324131
F	5.072757	1.588609	0.611756
F	4.252934	-1.706451	2.483508
F	-1.783109	-2.284211	3.946833
F	-0.293152	-1.622198	-4.929346
F	3.64764	-3.052565	0.911615
F	4.036004	1.159759	2.452467
F	-1.763008	-0.152846	3.580817
F	-1.415026	-0.573809	-3.402239
F	-2.446537	-1.49189	2.044732
F	0.212936	5.12389	-0.832955
F	3.351938	-3.602474	2.981264
F	4.837116	-1.154468	-3.408181
N	1.539567	-1.006064	1.274553
F	-1.098124	-2.698321	-3.240455
F	4.556698	3.197727	1.952396
F	-0.855975	3.3629	-1.507253
F	4.699421	0.341409	-1.855833
N	2.059304	1.24368	0.252243
N	0.263773	-0.695335	1.50926
N	2.002294	-0.800765	-1.215397
N	0.929237	1.739711	-0.255187
F	4.874283	-1.743189	-1.331996
F	-1.070775	4.051054	0.525087
N	0.734612	-0.972331	-1.59137

N	-3.192609	0.475743	-0.827867
C	2.069773	-1.351957	-3.367019
H	2.41396	-1.589916	-4.362595
C	0.965765	3.053695	-0.066911
C	0.759359	-1.29763	-2.880052
C	2.83295	-1.023421	-2.262446
C	-3.971629	-0.701179	-0.504849
C	2.812434	2.243285	0.770092
C	-0.145224	-1.475322	2.504909
C	1.941576	-1.984563	2.119454
C	0.88028	-2.321354	2.939755
H	0.859463	-3.061802	3.725602
C	-0.508928	-1.552792	-3.616568
C	2.144146	3.438852	0.584174
H	2.468066	4.428455	0.871454
C	-1.537764	-1.353205	3.022727
C	-5.176658	-0.632719	0.187251
H	-5.57224	0.327163	0.506145
C	-5.407825	-3.054006	0.071692
B	2.367189	-0.271363	0.193354
H	3.526273	-0.450288	0.394542
C	-3.490026	-1.942209	-0.923279
H	-2.550336	-2.005301	-1.465273
C	-0.192567	3.900728	-0.474673
C	-4.205733	-3.097801	-0.643812
H	-3.813449	-4.056522	-0.979493
C	4.320799	-0.894671	-2.200948

C	3.312261	-2.580575	2.118188
C	4.129963	2.035663	1.444304
C	-5.875788	-1.80373	0.475105
H	-6.813815	-1.735148	1.023805
C	-6.151628	-4.31551	0.409776
H	-6.189312	-5.001111	-0.445607
H	-7.181318	-4.10329	0.718459
H	-5.664758	-4.853877	1.234506
C	-3.595974	1.726107	-0.251927
C	-3.993673	2.779859	-1.070332
C	-3.547391	1.894916	1.134562
C	-4.332451	4.008121	-0.505642
H	-4.02925	2.644439	-2.150513
C	-3.90678	3.116539	1.690685
H	-3.225757	1.070489	1.766757
C	-4.297249	4.178761	0.874639
H	-4.630046	4.830173	-1.152503
H	-3.86525	3.242711	2.770061
H	-4.56484	5.136377	1.314354
H	-3.137308	0.566298	-1.839033

PBE0-D3/BS1:

Electronic energy = -3425.062548

Sum of electronic and zero-point energies = -3424.601009

Sum of electronic and thermal energy = -3424.55099

Sum of electronic and thermal enthalpy = -3424.550045

Sum of electronic and free energy = -3424.695427

Free Energy Correction = 0.36712129

PBE0-D3/BS2//PBE0-D3/BS1:

Electronic Energy = -3427.715326

Table S11. Optimized Coordinates of benzene

C	0.000000	1.394023	0.000000
C	1.207259	0.697011	0.000000
C	1.207259	-0.697011	0.000000
C	0.000000	-1.394023	0.000000
C	-1.207259	-0.697011	0.000000
C	-1.207259	0.697011	0.000000
H	0.000000	2.482184	0.000000
H	2.149635	1.241092	0.000000
H	2.149635	-1.241092	0.000000
H	0.000000	-2.482184	0.000000
H	-2.149635	-1.241092	0.000000
H	-2.149635	1.241092	0.000000

PBE0-D3/BS1:

Electronic energy = -231.9669722

Sum of electronic and zero-point energies = -231.865746

Sum of electronic and thermal energy = -231.861352

Sum of electronic and thermal enthalpy = -231.860408

Sum of electronic and free energy = -231.890863

Free Energy Correction = 0.076109155

PBE0-D3/BS2//PBE0-D3/BS1:

Electronic Energy = -232.0564146

Table S12. Optimized Coordinates of N_2

N	0.000000	0.000000	0.550016
N	0.000000	0.000000	-0.550016

PBE0-D3/BS1:

Electronic energy = -109.3993036

Sum of electronic and zero-point energies = -109.393601

Sum of electronic and thermal energy = -109.39124

Sum of electronic and thermal enthalpy = -109.390296

Sum of electronic and free energy = -109.412041

Free Energy Correction = -0.012737368

PBE0-D3/BS2//PBE0-D3/BS1:

Electronic Energy = -109.4463128

C.8. IR Spectroscopy

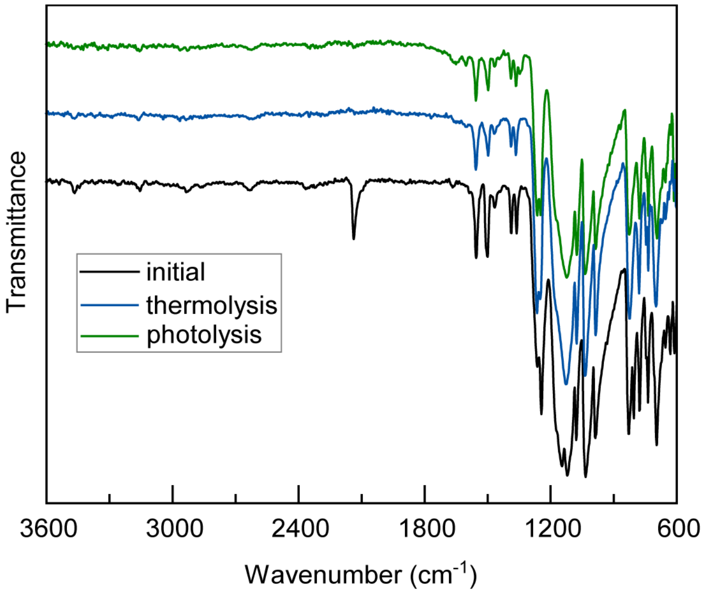

Figure S20. Solid-state ATR-IR spectrum of **2** after thermolysis at 100 °C for 20 min and photolysis (λ = 370 nm) for 8 h under N_2. In each case, 5 mg of the powdered sample was uniformly spread out on the inner wall of a 20-mL scintillation vial using a spatula and sealed under N_2 prior to thermolysis or photolysis. Both spectra show complete disappearances of the azide stretching frequencies.

C.9. Stoichiometric Reaction with 1

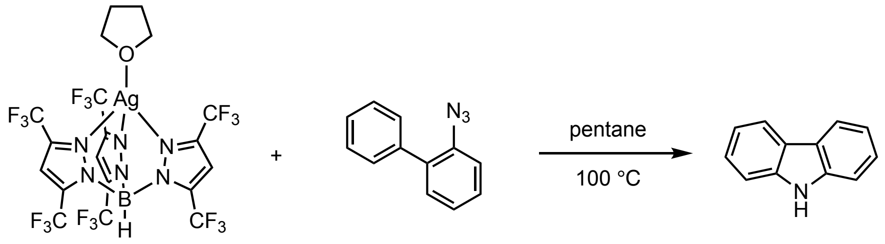

In an N_2-filled glovebox, a 25 mL Schlenk tube was charged with a magnetic stir bar, **1** (24.0 mg, 0.03 mmol, 1.0 equiv), biphenyl azide (**5a**, 0.03 mmol, 1.0 equiv), and dry pentane (5.0 mL). The tube was wrapped with aluminum foil to exclude ambient light. With stirring, the mixture was heated at 100 °C for 16 h. The mixture was allowed to cool to 23 °C and solvent was removed under reduced pressure. The residue was taken up in DMSO-d_6 and mesitylene was added as an internal standard. ^{1}H NMR of the crude reaction mixture showed the formation of **6a** with 95% yield. ^{1}H NMR (δ, 23 °C, 400 MHz, DMSO-d_6): 11.22 (br s, 1H), 8.13–8.06 (m, 2H), 7.50–7.46 (m, 2H), 7.37 (ddd, *J* = 8.1, 7.0, 1.2 Hz, 1H), 7.15 (ddd, *J* = 8.0, 7.1, 1.0 Hz, 2H). The obtained spectral data are in good agreement with those reported in literature.[31]

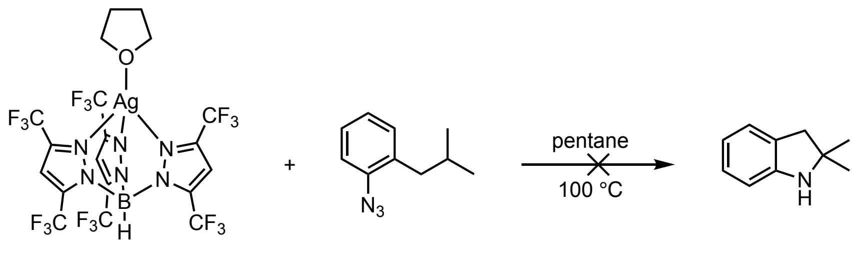

No productive reactivity was observed when 1-azido-2-isobutylbenzene was used as the substrate for intramolecular C(sp^3)–H amination.

D. General Procedure for Catalytic C–H Amination of Biaryl Azides

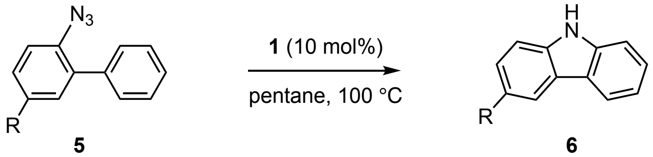

In an N_2-filled glovebox, a 25 mL Schlenk tube was charged with a magnetic stir bar, **1** (8.0 mg, 0.01 mmol, 0.10 equiv), the indicated aryl azide (**5**, 0.10 mmol, 1.0 equiv), and dry pentane (5.0 mL). The tube was wrapped with aluminum foil to exclude ambient light. With stirring, the mixture was heated at 100 °C for 16 h. The mixture was allowed to cool to 23 °C and solvent was removed under reduced pressure. The residue was taken up in DMSO-d_6 or $CDCl_3$ and mesitylene was added as an internal standard. The yield was calculated from the ^{1}H NMR of the crude reaction mixture.

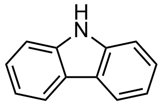

6a

9H-carbazole (6a) Prepared from 2-azido-1,1'-biphenyl (**5a**) and obtained in 74% yield. ^{1}H NMR (δ, 23 °C, 400 MHz, DMSO-d_6): 11.22 (br s, 1H), 8.13–8.06 (m, 2H), 7.50–7.46 (m, 2H), 7.37 (ddd, *J* = 8.1, 7.0, 1.2 Hz, 1H), 7.15 (ddd, *J* = 8.0, 7.1, 1.0 Hz, 2H). The obtained spectral data are in good agreement with those reported in literature.[31]

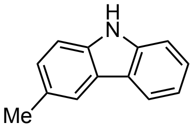

6b

3-Methyl-9H-carbazole (6b) Prepared from 2-azido-5-methyl-1,1'-biphenyl (**5b**) and obtained in 93% yield. ^{1}H NMR (δ, 23 °C, 400 MHz, DMSO-d_6): 11.08 (br s, 1H), 8.05 (d, *J* = 7.8 Hz, 1H), 7.89 (s, 1H), 7.45 (d, *J* = 8.1 Hz, 1H), 7.38–7.33 (m, 2H), 7.20 (dd, *J* = 8.2, 1.6 Hz, 1H), 7.12 (t, *J* = 7.0 Hz, 1H), 2.46 (s, 3H). The obtained spectral data are in good agreement with those reported in literature.[31]

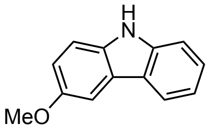

6c

3-Methoxy-9H-carbazole (6c) Prepared from 2-azido-5-methoxy-1,1'-biphenyl (**5c**) and obtained in 96% yield. ^{1}H NMR (δ, 23 °C, 400 MHz, $CDCl_3$): 8.06 (d, *J* = 7.8 Hz, 1H), 7.90 (br s, 1H), 7.57 (d, *J* = 2.5 Hz, 1H), 7.41 (dd, *J* = 4.7, 1.6 Hz, 2H), 7.32 (d, *J* = 8.7 Hz, 1H), 7.23 (ddd, *J* = 7.9, 5.7, 2.3 Hz, 1H), 7.08 (dd, *J* = 8.7, 2.5 Hz, 1H), 3.94 (s, 3H). The obtained spectral data are in good agreement with those reported in literature.[28]

E. General Procedure for Catalytic C–H Amination of 2-Alkenyl Arylazides

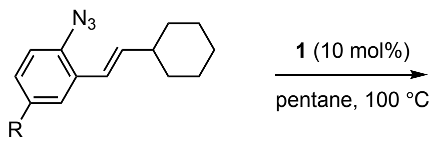

7 **8**

In an N_2-filled glovebox, a 25 mL Schlenk tube was charged with a magnetic stir bar, **1** (8.0 mg, 0.01 mmol, 0.10 equiv), the indicated 2-cyclohexylvinyl arylazide (**7**, 0.10 mmol, 1.0 equiv), and dry pentane (5.0 mL). The tube was wrapped with aluminum foil to exclude ambient light. With stirring, the mixture was heated at 100 °C and stirred for 16 h. The reaction mixture was allowed to cool to 23 °C and solvent was removed under reduced pressure. The residue was taken up in $CDCl_3$ and mesitylene was added as an internal standard. The yield was calculated from the ^1H NMR of the crude reaction mixture. For the new compounds, purification by flash column chromatography on silica gel (hexanes / ethyl acetate) afforded the corresponding products.

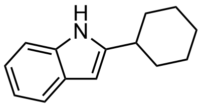

8a

2-cyclohexyl-1H-indole (8a) Prepared from (*E*)-1-azido-2-(2-cyclohexylvinyl)benzene (**7a**) and obtained in 71% yield. ^1H NMR (δ, 23 °C, 400 MHz, $CDCl_3$): 7.91 (br s, 1H), 7.54 (d, *J* = 7.6 Hz, 1H), 7.31 (d, *J* = 8.0 Hz, 1H), 7.13–7.05 (m, 2H), 6.24 (s, 1H), 2.75–2.69 (m, 1H), 2.10–2.07 (m, 2H), 1.88–1.84 (m, 2H), 1.77–1.73 (m, 1H), 1.52–1.21 (m, 5H). The obtained spectral data are in good agreement with those reported in literature.[28]

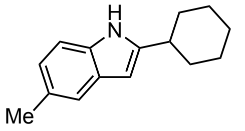

8b

2-cyclohexyl-5-methyl-1H-indole (8b) Prepared from (*E*)-1-azido-2-(2-cyclohexylvinyl)-4-methylbenzene (**7b**) and obtained in 91% yield. ^{1}H NMR (δ, 23 °C, 400 MHz, $CDCl_3$): 7.78 (br s, 1H), 7.34 (s, 1H), 7.19 (d, *J* = 8.2 Hz, 1H), 7.01–6.90 (m, 1H), 6.17 (t, *J* = 1.1 Hz, 1H), 2.77–2.64 (m, 1H), 2.44 (s, 3H), 2.10–2.07 (m, 2H), 1.88–1.85 (m, 2H), 1.81–1.72 (m, 1H), 1.57–1.30 (m, 5H). ^{13}C NMR (δ, 23 °C, 100 MHz, $CDCl_3$): 145.3, 133.9, 129.0, 128.8, 122.5, 119.8, 110.1, 97.1, 37.5, 33.1, 26.4, 26.3, 21.6.

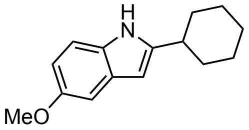

8c

2-cyclohexyl-5-methoxy-1H-indole (8c) Prepared from (*E*)-1-azido-2-(2-cyclohexylvinyl)-4-methoxybenzene (**7c**) and obtained in 87% yield. ^{1}H NMR (δ, 23 °C, 400 MHz, $CDCl_3$): 7.80 (br s, 1H), 7.18 (d, *J* = 8.7 Hz, 1H), 7.04 (d, *J* = 2.0 Hz, 1H), 6.79 (dd, *J* = 8.7, 2.4 Hz, 1H), 6.18 (s, 1H), 3.85 (s, 3H), 2.74–2.63 (m, 1H), 2.07 (d, *J* = 11.7 Hz, 2H), 1.86 (d, *J* = 12.1 Hz, 2H), 1.81–1.72 (m, 1H), 1.55–1.28 (m, 5H). ^{13}C NMR (δ, 23 °C, 100 MHz, $CDCl_3$): 154.2, 146.1, 130.8, 129.2, 111.1, 110.9, 102.3, 97.5, 56.1, 37.6, 33.1, 26.4, 26.2.

F. NMR Spectra

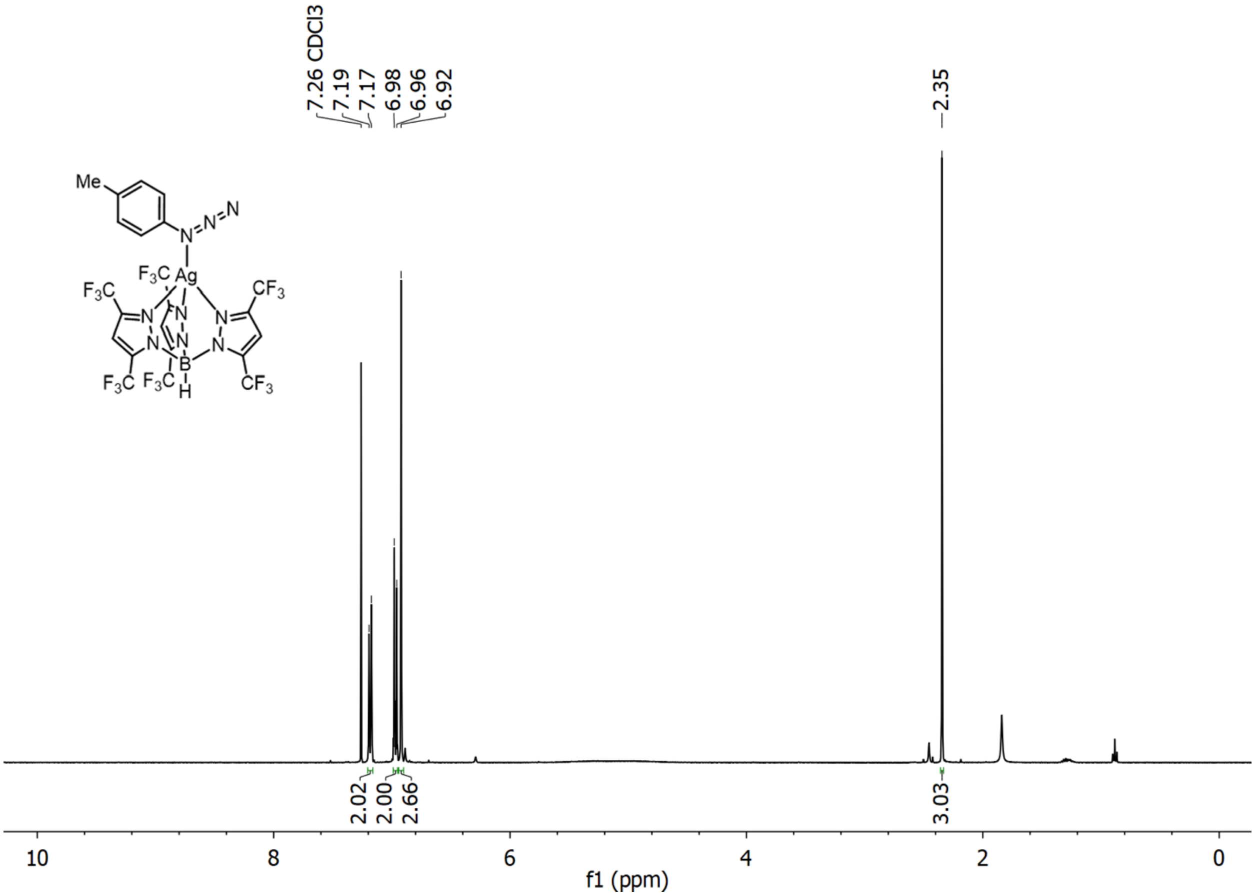

Figure S21. ^{1}H NMR spectrum of **2** in $CDCl_3$ (400 MHz) at 23 °C.

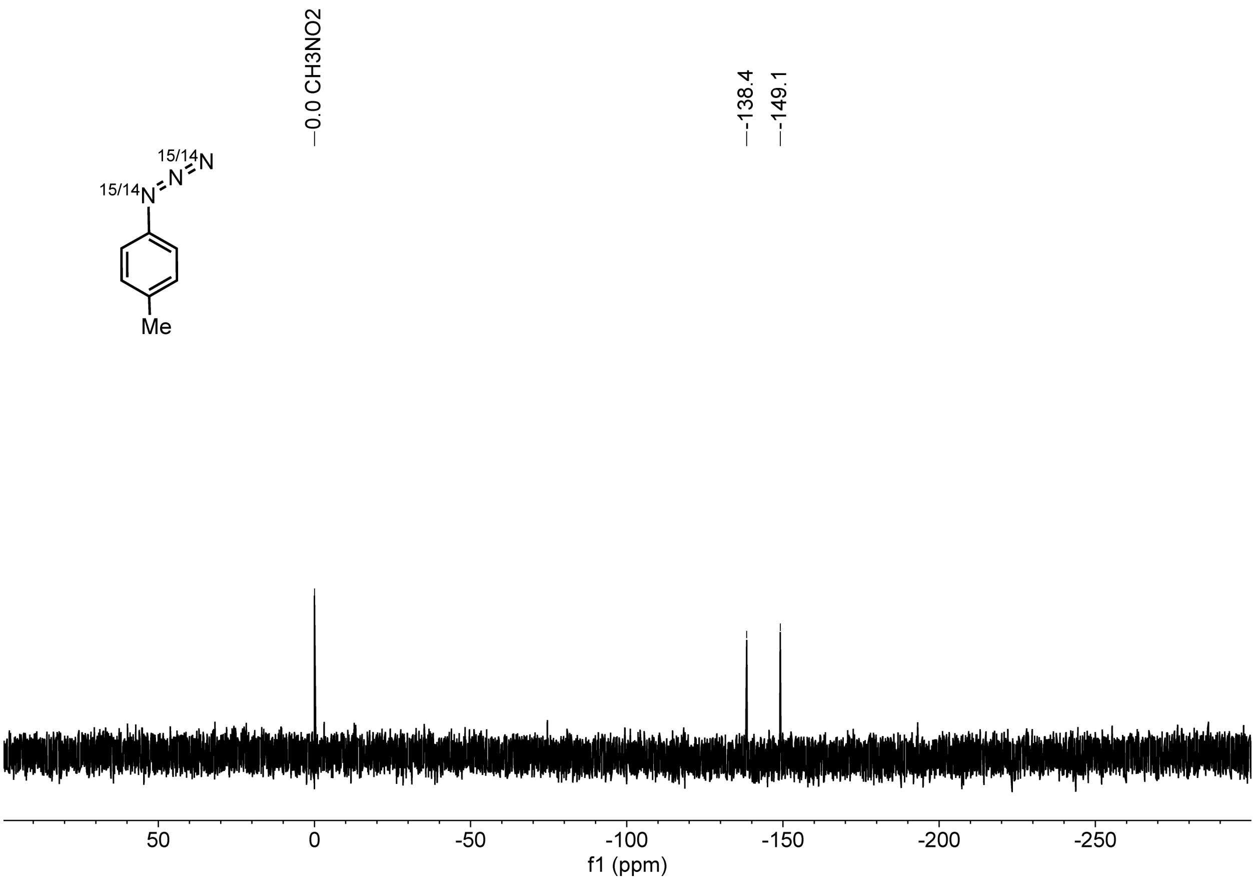

Figure S22. $^{15}N\{^{1}H\}$ NMR spectrum of (4-MePh^{15}NN$_2$) in $CDCl_3$ (50.7 MHz) at 23 °C referenced against CH_3NO_2 at 0.0 ppm.

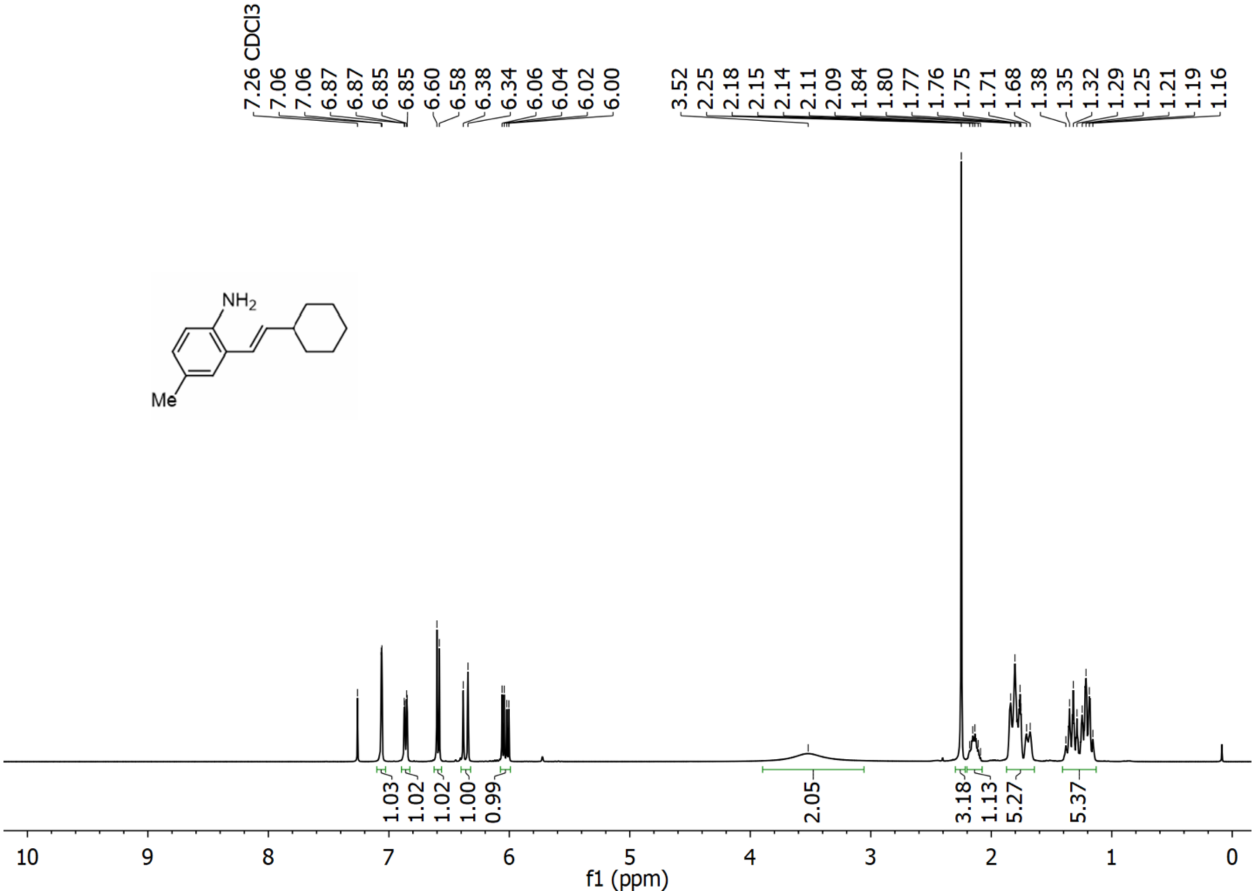

Figure S23. ^{1}H NMR spectrum of **7b'** in $CDCl_3$ (400 MHz) at 23 °C.

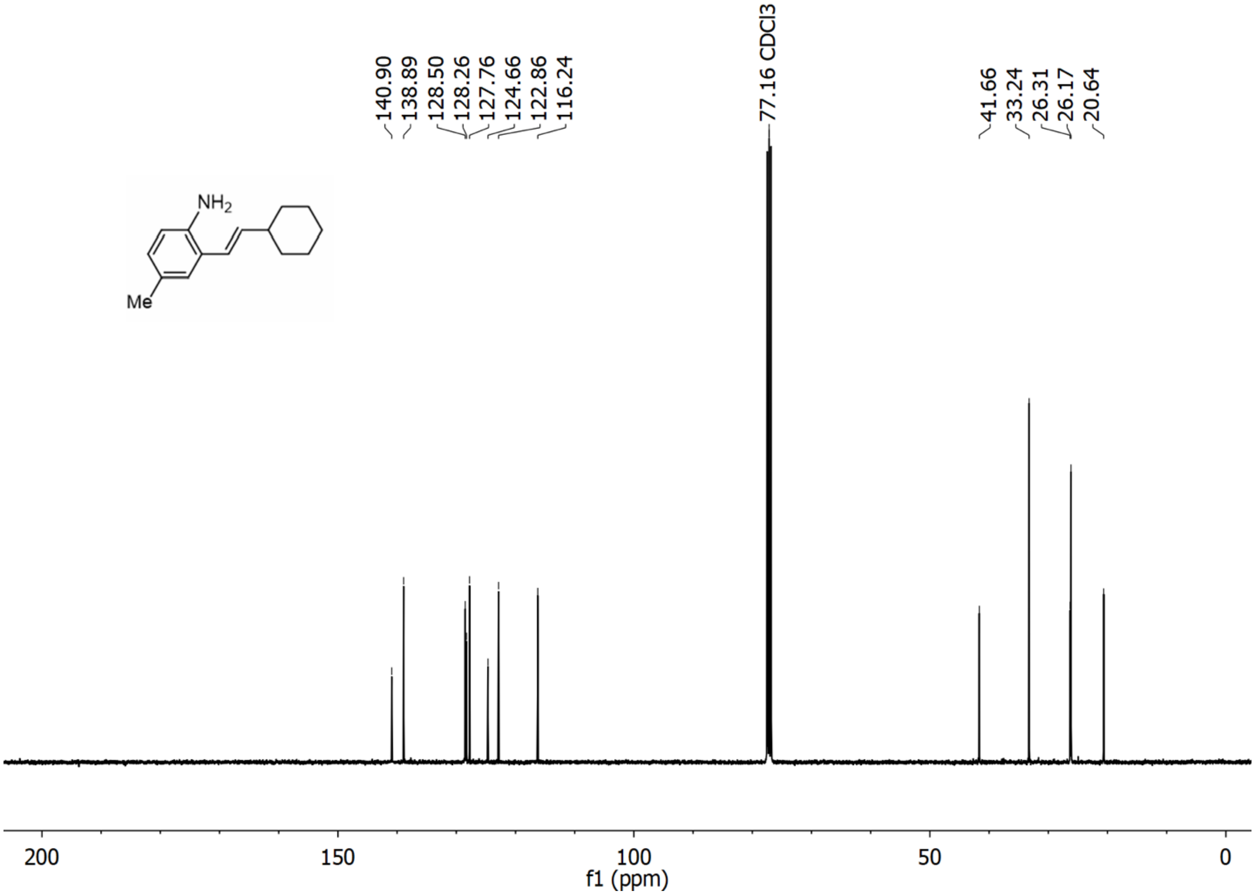

Figure S24. ^{13}C NMR spectrum of **7b'** in $CDCl_3$ (100 MHz) at 23 °C.

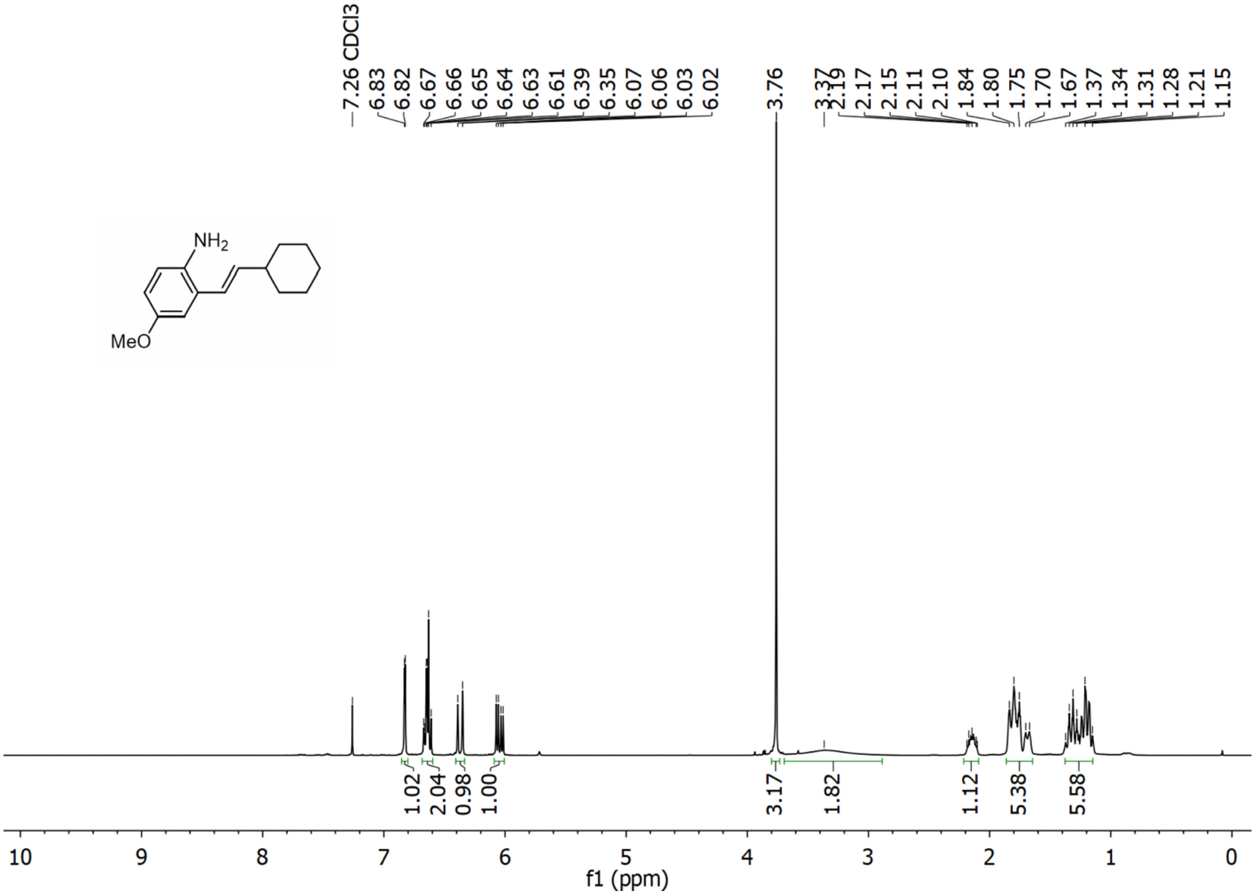

Figure S25. ^{1}H NMR spectrum of **7c'** in $CDCl_3$ (400 MHz) at 23 °C.

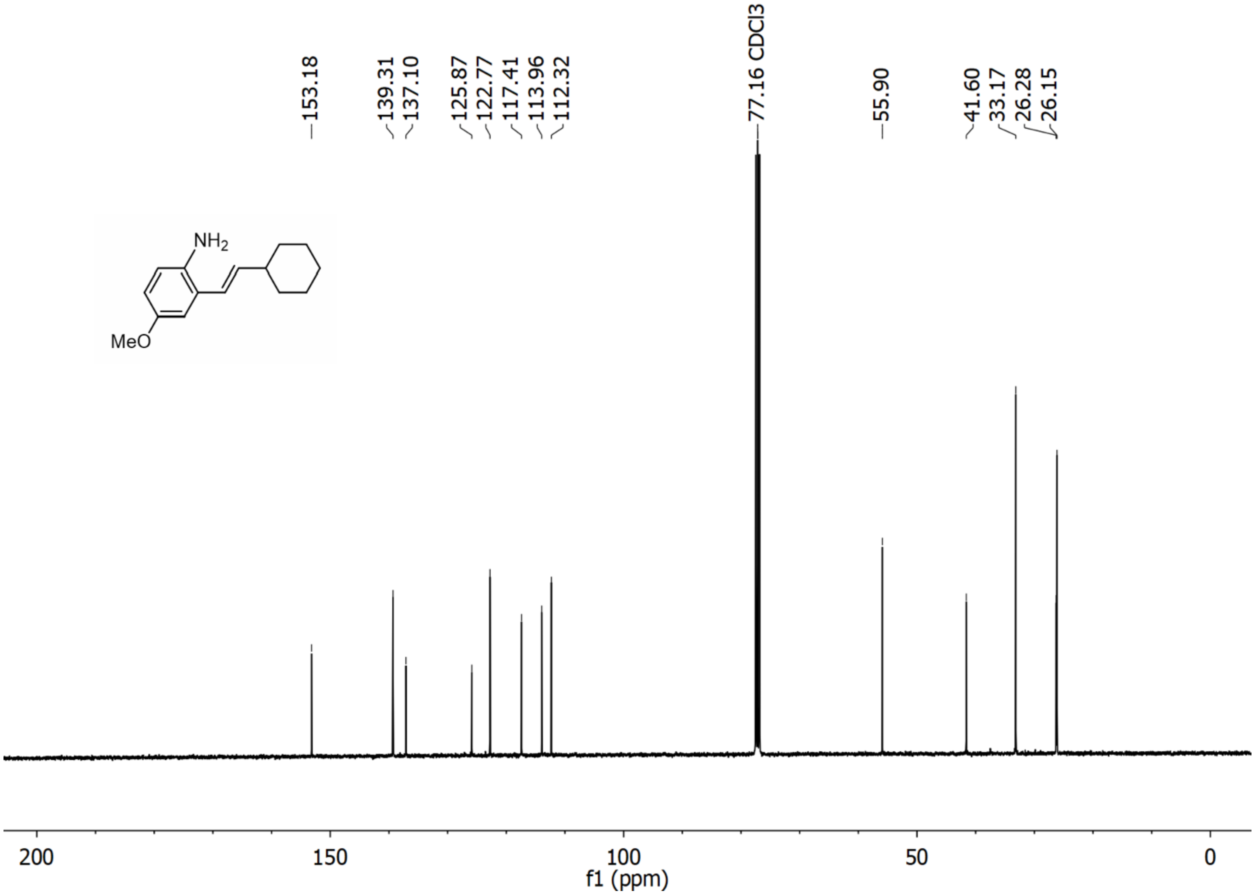

Figure S26. ^{13}C NMR spectrum of **7c'** in $CDCl_3$ (100 MHz) at 23 °C.

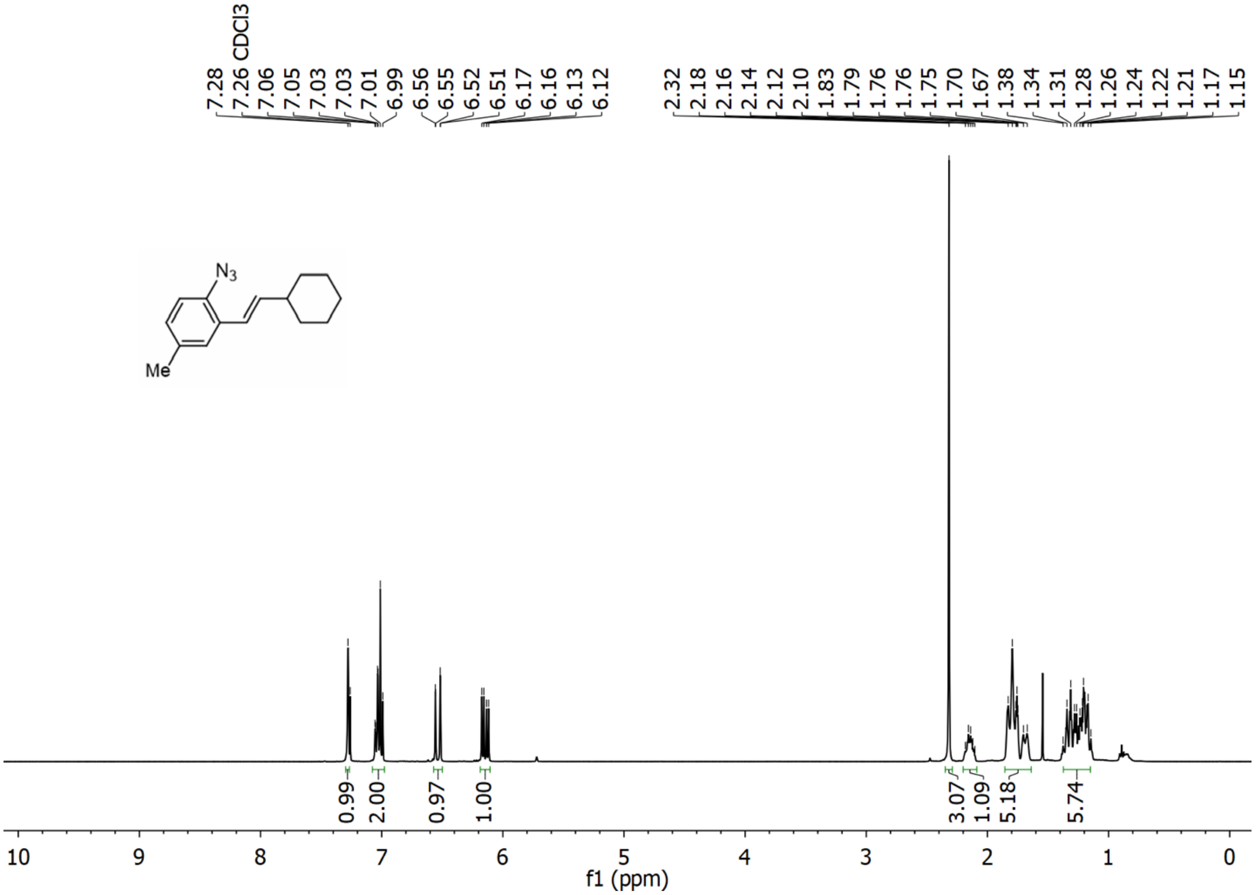

Figure S27. ^{1}H NMR spectrum of **7b** in $CDCl_3$ (400 MHz) at 23 °C.

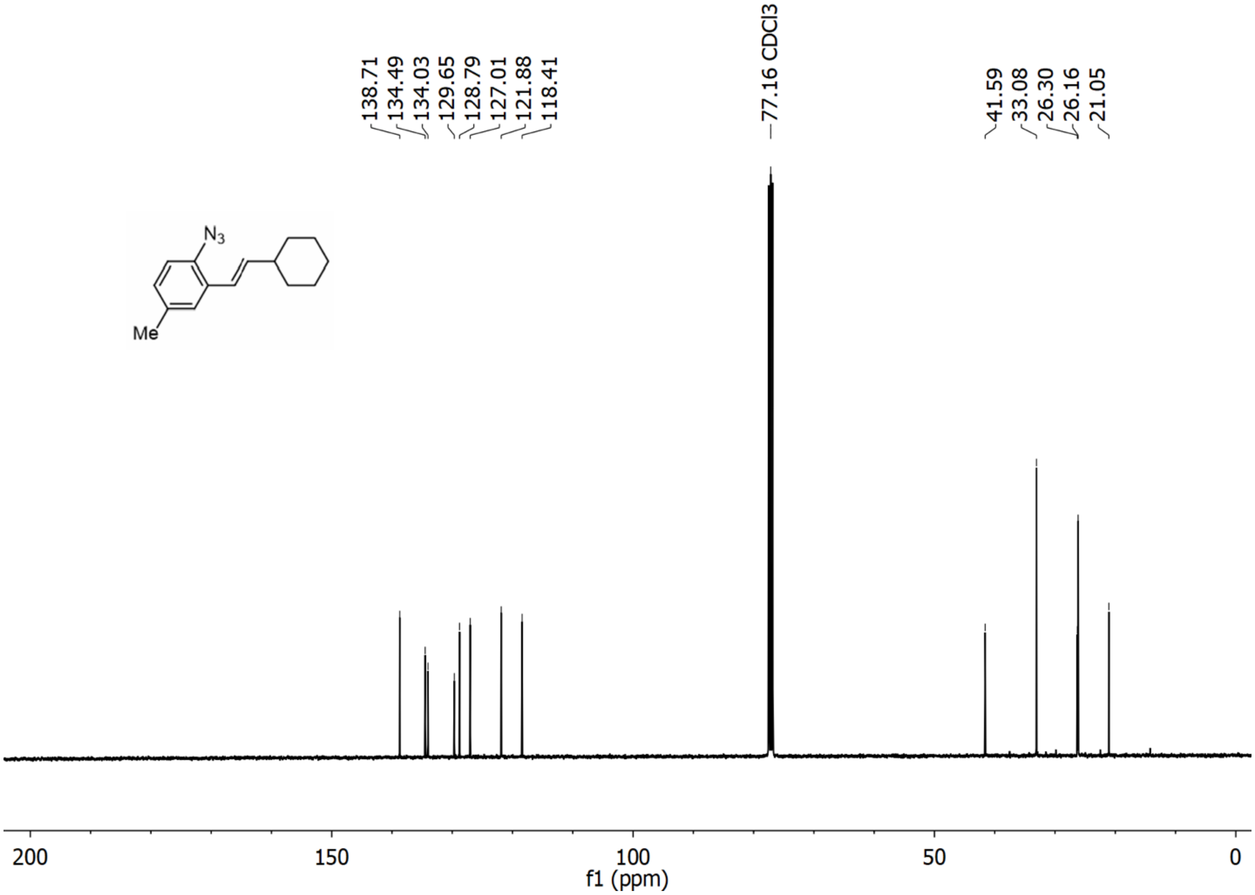

Figure S28. ^{13}C NMR spectrum of **7b** in $CDCl_3$ (100 MHz) at 23 °C.

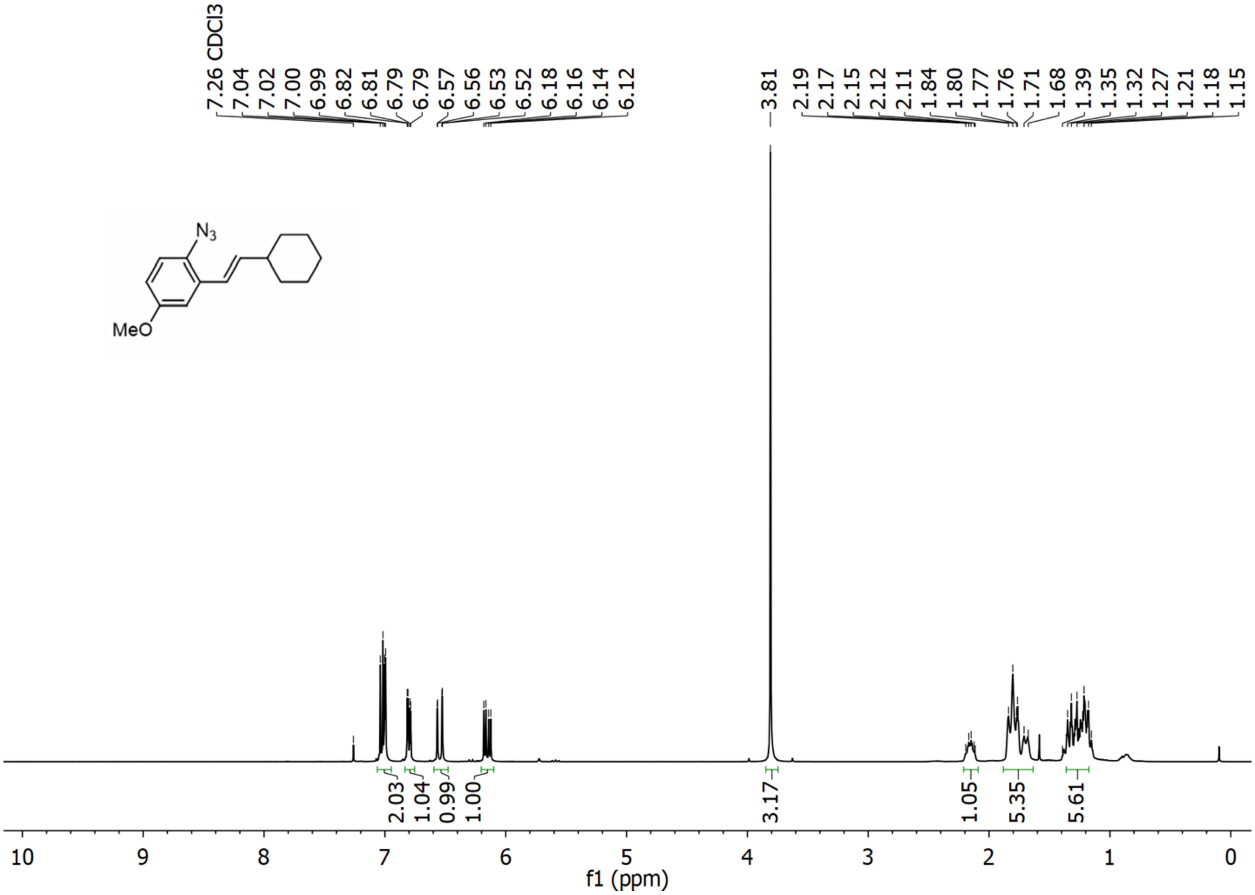

Figure S29. ^{1}H NMR spectrum of **7c** in $CDCl_3$ (400 MHz) at 23 °C.

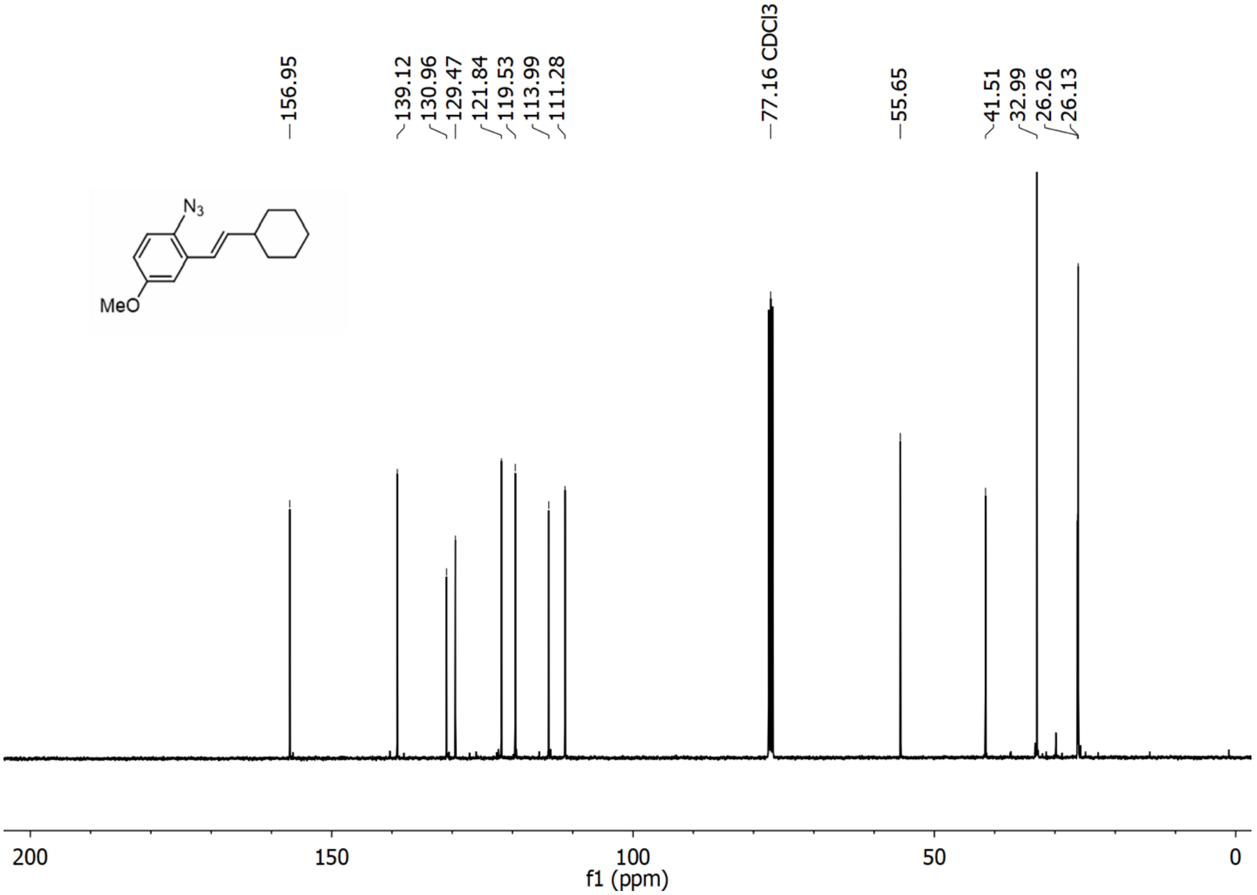

Figure S30. ^{13}C NMR spectrum of **7c** in $CDCl_3$ (100 MHz) at 23 °C.

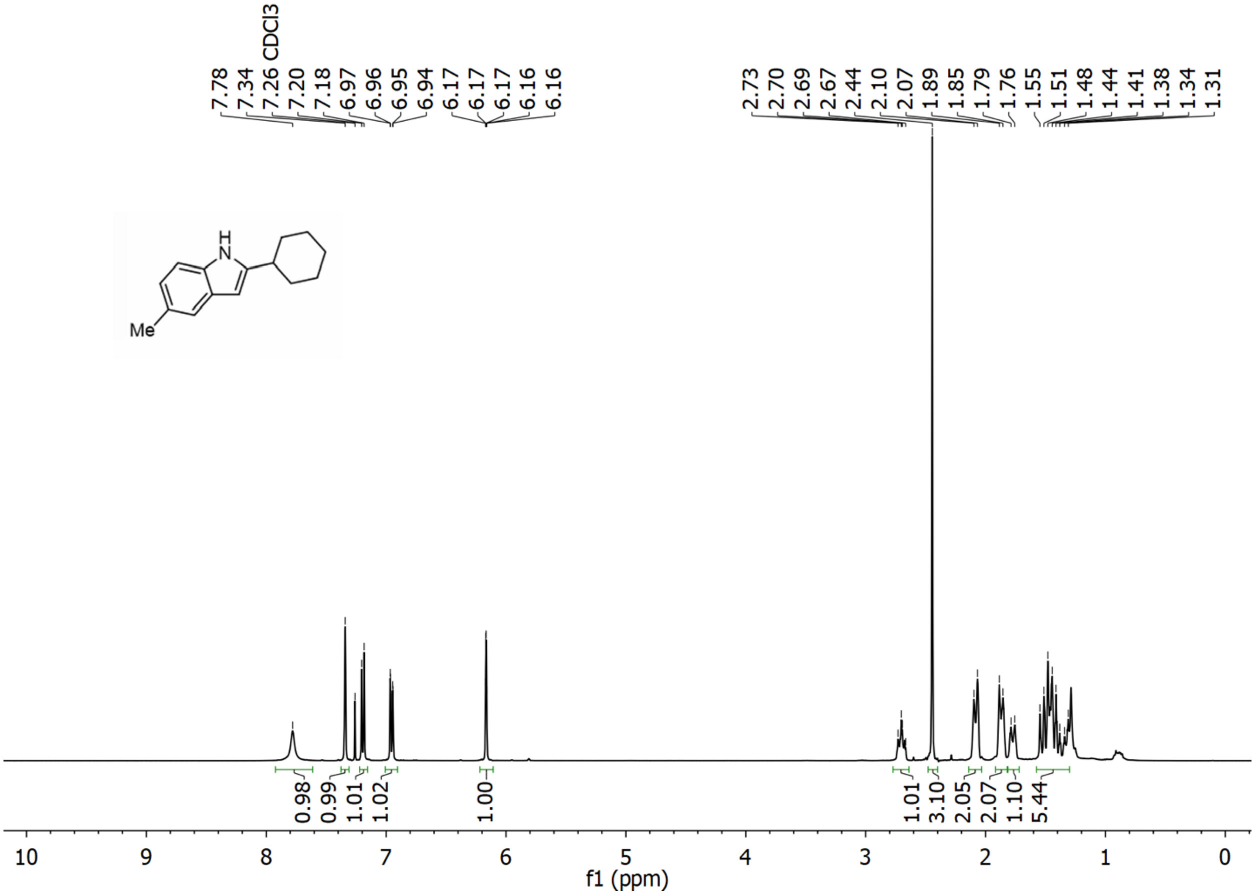

Figure S31. ^{1}H NMR spectrum of **8b** in $CDCl_3$ (400 MHz) at 23 °C.

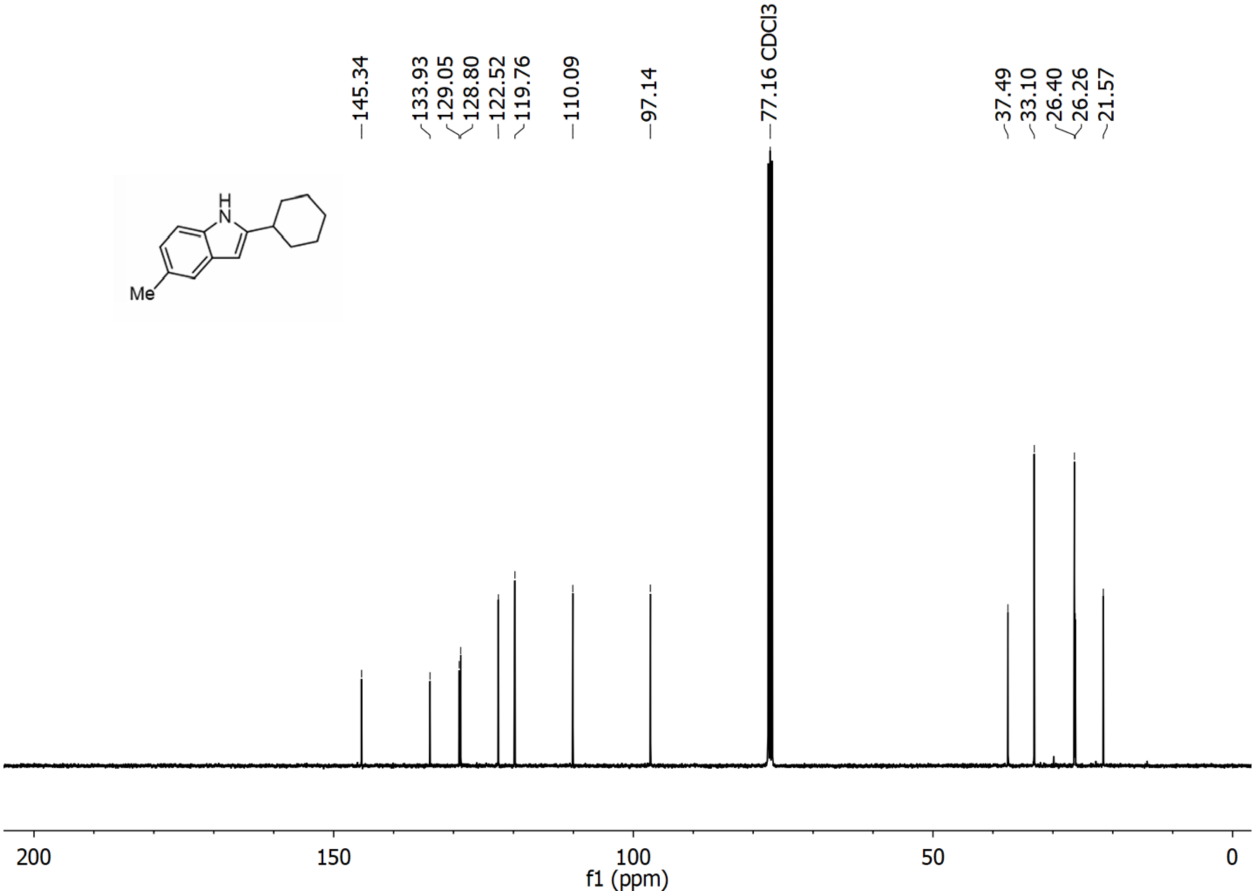

Figure S32. ^{13}C NMR spectrum of **8b** in $CDCl_3$ (100 MHz) at 23 °C.

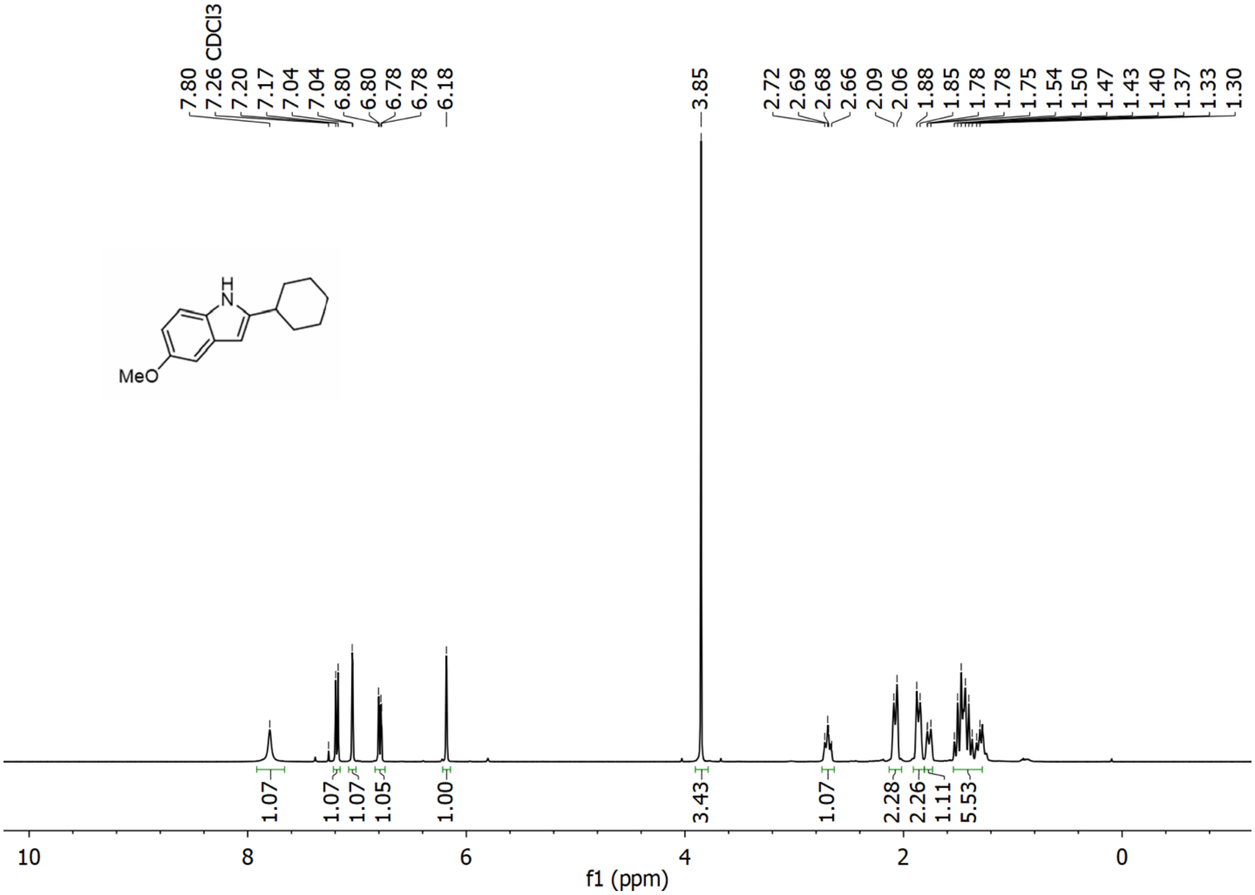

Figure S33. ^{1}H NMR spectrum of **8c** in $CDCl_3$ (400 MHz) at 23 °C.

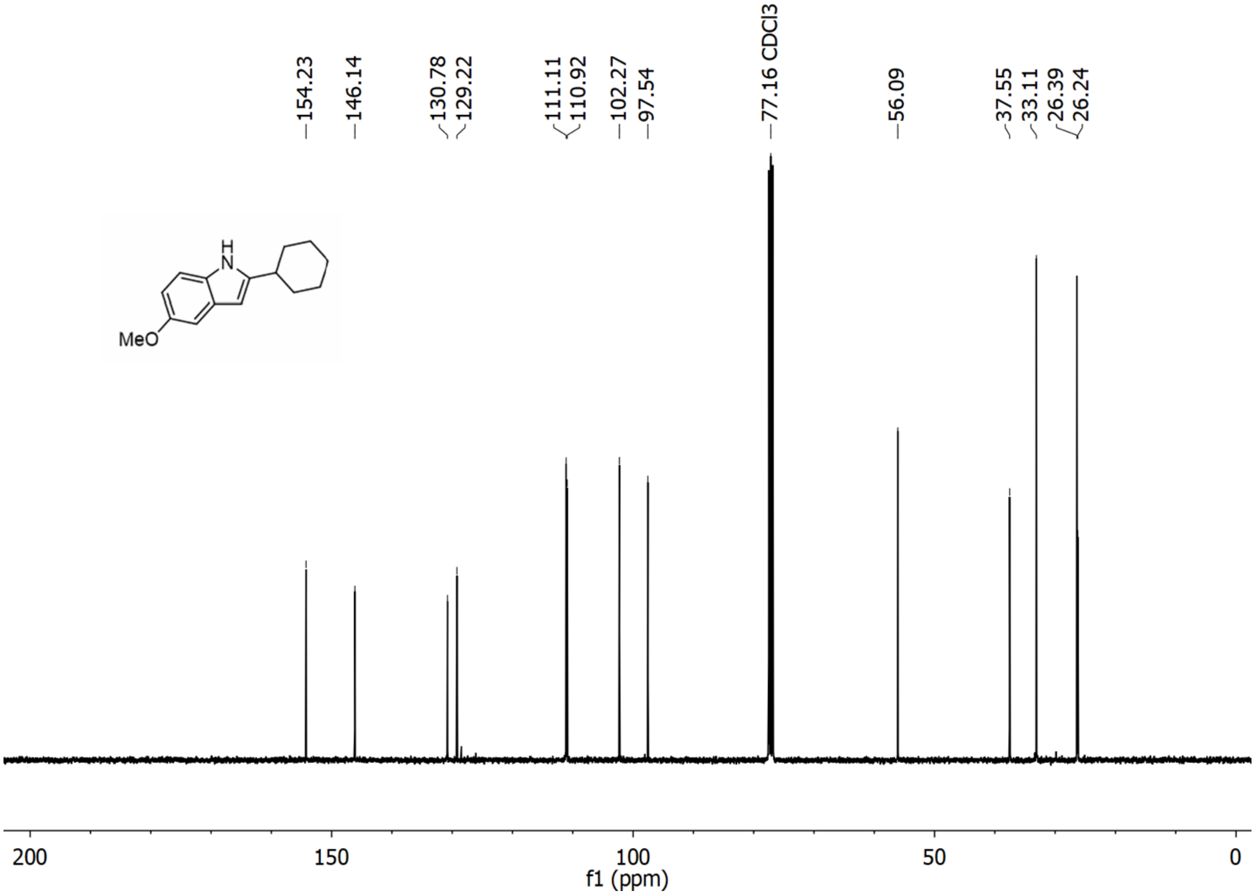

Figure S34. ^{13}C NMR spectrum of **8c** in $CDCl_3$ (100 MHz) at 23 °C.

G. X-Ray Crystallographic Data

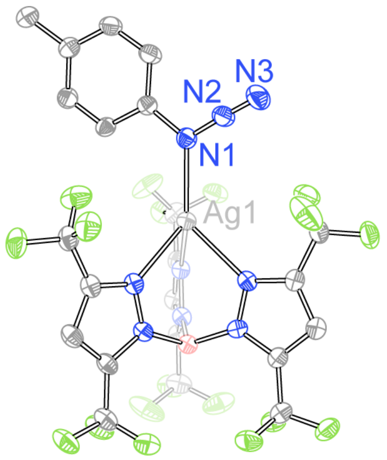

Figure S35. Displacement ellipsoid plot of the asymmetric unit of **2** plotted at 50% probability. H-atoms are removed for clarity. Single crystals suitable for X-ray diffraction were grown from a pentane solution of **2** at –25 °C in an N_2-filled glovebox freezer.

Table S13. X-ray experimental details of **2** (CCDC 2535939).

Crystal data	
**Chemical formula**	$C_{22}H_{11}AgBF_{18}N_9$
M_r	862.08
**Crystal system, space group**	Triclinic, *P*1
**Temperature (K)**	100
***a*, *b*, *c* (Å)**	8.1823(2), 8.9291(2), 22.5284(5)
**α, β, γ (°)**	87.045(2), 81.258(2), 63.007(3)
***V* (Å^3)**	1449.31(7)
Z	2
**Radiation type**	Cu *K*α
**μ (mm^{-1})**	7.00
**Crystal size (mm)**	0.09 × 0.08 × 0.02
Data collection	
**Diffractometer**	XtaLAB Synergy, Dualflex, HyPix
**Absorption correction**	Multi-scan<br>*CrysAlis PRO* 1.171.43.128a (Rigaku Oxford Diffraction, 2024) Empirical absorption correction using spherical harmonics, implemented in SCALE3 ABSPACK scaling algorithm.
T_{min}, T_{max}	0.783, 1.000
**No. of measured, independent and observed [$I > 2\sigma(I)$] reflections**	16523, 6093, 5710
R_{int}	0.054
**$(\sin \theta/\lambda)_{max}$ (Å^{-1})**	0.639
**Refinement**	
**$R[F^2 > 2\sigma(F^2)]$, $wR(F^2)$, S**	0.051, 0.138, 1.07
**No. of reflections**	6093
**No. of parameters**	461
**H-atom treatment**	H-atom parameters constrained

**$\Delta\rho_{max}$, $\Delta\rho_{min}$ (e Å^{-3})**	1.58, −1.21

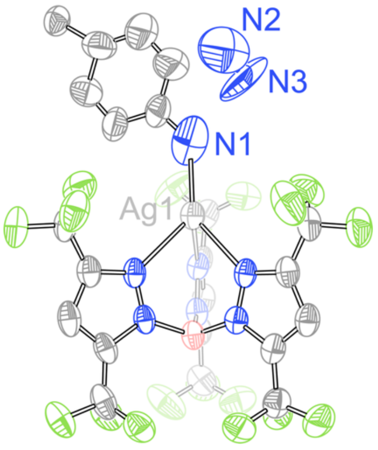

Figure S36. Displacement ellipsoid plot of the asymmetric unit of **4** plotted at 50% probability. H-atoms are removed for clarity. A 80 × 70 × 20 μm crystal of **2** was mounted on the goniometer using a MiTeGen Dual Thickness MicroMounts loop. The temperature was maintained at 100 K throughout the experiment. A THORLABS M365FP1 365 nm fiber-coupled LED (9.8 mW) was positioned in front of the goniometer and secured with a clamp. The crystal was irradiated for 2 minutes while being rotated to ensure uniform photolysis. After irradiation, the lamp was turned off, and diffraction data were collected.

The structure of **4** was refined as a disordered model of **2** (Part 1) and **4** (Part 2) with an occupancy ratio close to 0.6:0.4. For the final least square refinement cycles, the occupancies were fixed at that ratio. Geometrical restraints (DFIX) were applied to Part 1 to maintain the N–N, N–C_{Ar}, and Ag–N distances same as those in the dark structure (**2**). Displacement parameter restraints (SIMU/DELU in Part 1 and SIMU in Part 2) were used to stabilize this refinement. The extruded N_2 was modeled at a 20% occupancy with the N–N bond constrained at 1 Å (DFIX). We note that attempts to refine the extruded N_2 fragment with higher occupancy or fewer constraints led to instability in the model. Accordingly, the structural model should be interpreted as a superposition of a partially converted **4** (40% occupancy) with **2** (60% occupancy), rather than a fully converted single-component species (Figure S37). In the refined structure of **4**, the distances between N1 and the two nitrogen atoms of the extruded N_2 fragment are 3.386 and 2.852 Å, respectively, indicating no significant interaction between N1 and the extruded N_2 fragment.

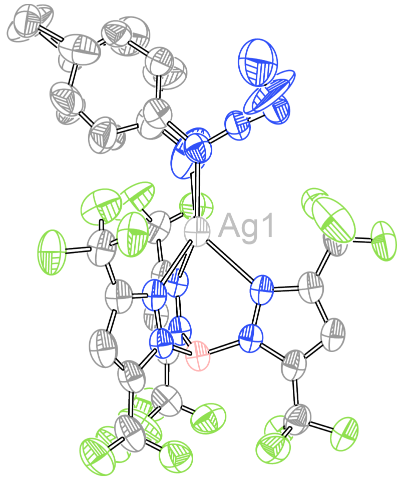

Figure S37. Displacement ellipsoid plot of disordered model with both the unreacted azide complex (**2**, 60%) and the photogenerated nitrene (**4**, 40%) overlaid within the same lattice (plotted at 50% probability).

Table S14. X-ray experimental details of **4** (CCDC 2535947).

Crystal data	
**Chemical formula**	$C_{22}H_{11}AgBF_{18}N_{8.2}\cdot0.2(N_2)$
M_r	856.47
**Crystal system, space group**	Triclinic, *P*1
**Temperature (K)**	100
***a*, *b*, *c* (Å)**	8.2087(4), 8.9078(4), 22.7363(9)
**α, β, γ (°)**	84.647(3), 81.064(4), 62.768(5)
***V* (Å³)**	1459.84(12)
Z	2
**Radiation type**	Cu *K*α
**μ (mm⁻¹)**	6.94
**Crystal size (mm)**	0.8 × 0.7 × 0.2
Data collection	
**Diffractometer**	XtaLAB Synergy, Dualflex, HyPix
**Absorption correction**	Multi-scan<br>*CrysAlis PRO* 1.171.43.128a (Rigaku Oxford Diffraction, 2024) Empirical absorption correction using spherical harmonics, implemented in SCALE3 ABSPACK scaling algorithm.
T_{min}, T_{max}	0.705, 1.000
**No. of measured, independent and observed [$I > 2\sigma(I)$] reflections**	16143, 6112, 4950
R_{int}	0.077
**$(\sin\theta/\lambda)_{max}$ (Å⁻¹)**	0.640
**Refinement**	
$R[F^2 > 2\sigma(F^2)]$, $wR(F^2)$, S	0.069, 0.190, 1.02
**No. of reflections**	6112
**No. of parameters**	528
**No. of restraints**	80

**H-atom treatment**	H-atom parameters constrained
**$\Delta\rho_{max}$, $\Delta\rho_{min}$ (e Å^{-3})**	1.61, −1.08

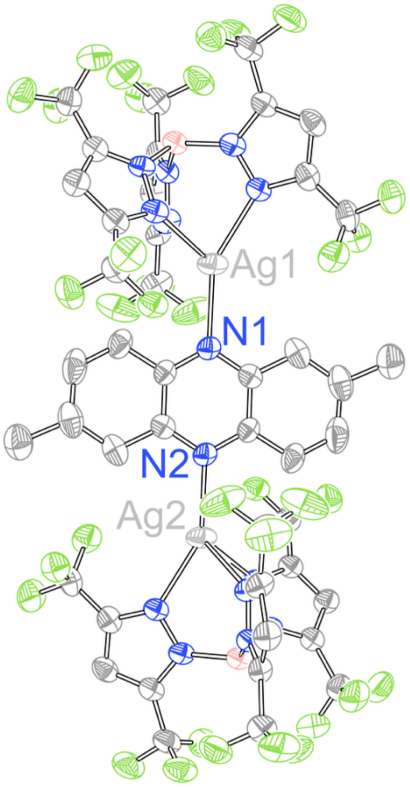

Figure S38. Displacement ellipsoid plot of the asymmetric unit of **3** plotted at 50% probability. H-atoms are removed for clarity.

Table S15. X-ray experimental details of **3** (CCDC 2535934).

Crystal data	
**Chemical formula**	$C_{44}H_{20}Ag_2B_2F_{36}N_{14}$
M_r	1666.10
**Crystal system, space group**	Monoclinic, $P2_1/c$
**Temperature (K)**	100
***a*, *b*, *c* (Å)**	19.0000(9), 8.4199(3), 18.8582(11)
**β (°)**	113.152(6)
***V* (Å^3)**	2773.9(3)
Z	2
**Radiation type**	Cu *K*α
**μ (mm^{-1})**	7.27
**Crystal size (mm)**	0.08 × 0.06 × 0.01
Data collection	
**Diffractometer**	XtaLAB Synergy, Dualflex, HyPix
**Absorption correction**	Multi-scan<br>*CrysAlis PRO* 1.171.43.142a (Rigaku Oxford Diffraction, 2024) Empirical absorption correction using spherical harmonics, implemented in SCALE3 ABSPACK scaling algorithm.
T_{min}, T_{max}	0.800, 1.000
**No. of measured, independent and observed [$I > 2\sigma(I)$] reflections**	18472, 5817, 5005
R_{int}	0.051
**$(\sin \theta/\lambda)_{max}$ (Å^{-1})**	0.638
**Refinement**	
**$R[F^2 > 2\sigma(F^2)]$, $wR(F^2)$, S**	0.089, 0.246, 1.13
**No. of reflections**	5817
**No. of parameters**	443
**H-atom treatment**	H-atom parameters constrained

	$w = 1/[\sigma^2(F_o^2) + (0.1105P)^2 + 20.9536P]$<br>where $P = (F_o^2 + 2F_c^2)/3$
**$\Delta\rho_{max}$, $\Delta\rho_{min}$ (e Å^{-3})**	2.50, −1.99

H. References

1. Pangborn, A. B.; Giardello, M. A.; Grubbs, R. H.; Rosen, R. K.; Timmers, F. J. Safe and convenient procedure for solvent purification. *Organometallics* **1996**, *15*, 1518–1520.
2. Sebest, F.; Casarrubios, L.; Rzepa, H. S.; White, A. J. P.; Díez-González, S. Thermal azide–alkene cycloaddition reactions: straightforward multi-gram access to Δ2-1,2,3-triazolines in deep eutectic solvents. *Green Chem.* **2018**, *20*, 4023–4035.
3. Fulmer, G. R.; Miller, A. J. M.; Sherden, N. H.; Gottlieb, H. E.; Nudelman, A.; Stoltz, B. M.; Bercaw, J. E.; Goldberg, K. I. NMR Chemical Shifts of Trace Impurities: Common Laboratory Solvents, Organics, and Gases in Deuterated Solvents Relevant to the Organometallic Chemist. *Organometallics* **2010**, *29*, 2176–2179.
4. Butzlaff, C. H.; Trautwein, A. X.; Winkler, H. [15] Magnetic susceptibility. In Methods in Enzymology, Academic Press: **1993**; *227*, 412–437.
5. Bill, E. mpView.1.4.1, Program for Viewing and Data Import for Files from MPMS3 SQUID Magnetometer, Max-Planck Institute for Chemical Energy Conversion, Mülheim/Ruhr, **2021**.
6. Bill, E. julX, Program for Simulation of Molecular Magnetic Data, Max-Planck Institute for Chemical Energy Conversion, Mülheim/Ruhr, **2008**.
7. Dolomanov, O. V.; Bourhis, L. J.; Gildea, R. J.; Howard, J. A. K.; Puschmann, H. OLEX2: a complete structure solution, refinement and analysis program. *J. Appl. Crystallogr.* **2009**, *42*, 339–341.
8. Sheldrick, G. M. A short history of SHELX. *Acta. Crystallogr.* **2008**, *64*, 1112–1122.
9. Frisch, M. J.; Trucks, G. W.; Schlegel, H. B.; Scuseria, G. E.; Robb, M. A.; Cheeseman, J. R.; Scalmani, G.; Barone, V.; Petersson, G. A.; Nakatsuji, H.; Li, X.; Caricato, M.; Marenich, A. V.; Bloino, J.; Janesko, B. G.; Gomperts, R.; Mennucci, B.; Hratchian, H. P.; Ortiz, J. V.; Izmaylov, A. F.; Sonnenberg, J. L.; Williams; Ding, F.; Lipparini, F.; Egidi, F.; Goings, J.; Peng, B.; Petrone, A.; Henderson, T.; Ranasinghe, D.; Zakrzewski, V. G.; Gao, J.; Rega, N.; Zheng, G.; Liang, W.; Hada, M.; Ehara, M.; Toyota, K.; Fukuda, R.; Hasegawa, J.; Ishida, M.; Nakajima, T.; Honda, Y.; Kitao, O.; Nakai, H.; Vreven, T.; Throssell, K.; Montgomery Jr. J. A.; Peralta, J. E.; Ogliaro, F.; Bearpark, M. J.; Heyd, J. J.; Brothers, E. N.; Kudin, K. N.; Staroverov, V. N.; Keith, T. A.; Kobayashi, R.; Normand, J.; Raghavachari, K.; Rendell, A.

P.; Burant, J. C.; Iyengar, S. S.; Tomasi, J.; Cossi, M.; Millam, J. M.; Klene, M.; Adamo, C.; Cammi, R.; Ochterski, J. W.; Martin, R. L.; Morokuma, K.; Farkas, O.; Foresman, J. B.; Fox, D. J. Gaussian 16 Rev. C.01, Wallingford, CT, **2016**.

10. Becke, A. D. Densityfunctional thermochemistry. III. the role of exact exchange. *J. Chem. Phys.* **1993,** *98*, 5648–5652.

11. Grimme, S. Semiempirical GGA-type density functional constructed with a long-range dispersion correction. *J. Comput. Chem.* **2006**, *27*, 1787–1799.

12. Grimme, S.; Ehrlich, S.; Goerigk, L. Effect of the damping function in dispersion corrected density functional theory. *J. Comput. Chem.* **2011**, *32*, 1456–1465.

13. Couty, M.; Hall, M. B. Basis sets for transition metals: Optimized outer p functions. *J. Comput. Chem.* **1996**, *17*, 1359–1370.

14. Hay, P. J.; Wadt, W. R. Ab initio effective core potentials for molecular calculations. Potentials for K to Au including the outermost core orbitals. *J. Chem. Phys.* **1985**, *82*, 299–310.

15. Petersson, G. A.; Al-Laham, M. A. A complete basis set model chemistry. II. Open-shell systems and the total energies of the first-row atoms. *J. Chem. Phys.* **1991**, *94*, 6081–6090.

16. Hehre, W. J.; Ditchfield, R.; Pople, J. A. Self—Consistent Molecular Orbital Methods. XII. Further Extensions of Gaussian—Type Basis Sets for Use in Molecular Orbital Studies of Organic Molecules. *J. Chem. Phys.* **1972**, *56*, 2257–2261.

17. Hariharan, P. C.; Pople, J. A. The influence of polarization functions on molecular orbital hydrogenation energies. *Theor. Chim. Acta* **1973**, *28*, 213–222.

18. Weigend, F.; Ahlrichs, R. Balanced basis sets of split valence, triple zeta valence and quadruple zeta valence quality for H to Rn: Design and assessment of accuracy. *Phys. Chem. Chem. Phys.* **2005**, *7*, 3297–3305.

19. Andrae, D.; Häußermann, U.; Dolg, M.; Stoll, H.; Preuß, H. Energy-adjustedab initio pseudopotentials for the second and third row transition elements. *Theor. Chim. Acta* **1990**, *77*, 123–141.

20. Neese, F. The ORCA program system. *WIREs Comput. Mol. Sci.* **2012**, *2*, 73–78.

21. Hellweg, A.; Hättig, C.; Höfener, S.; Klopper, W. Optimized accurate auxiliary basis sets for RI-MP2 and RI-CC2 calculations for the atoms Rb to Rn. *Theor. Chem. Acc.* **2007**, *117*, 587–597.

22. Glendening, E.D.; Badenhoop, J.K.; Reed, A.E.; Carpenter, J.E.; Bohmann, J.A.; Morales, C.M.; Karafiloglou, P.; Landis, C.R.; Weinhold, F. Theoretical Chemistry Institute, University of Wisconsin, Madison (**2018**).

23. Marques, M. A. L.; Gross, E. K. U. Time-Dependent Density Functional Theory. *Annu. Rev. Phys. Chem.* **2004**, 55, 427–455.

24. Fortran, I.; Press, W.; Teukolsky, S.; Vetterling, W.; Flannery, B. Numerical Recipes. Cambridge, UK, Cambridge University Press **1992**.

25. Dennington, R.; Keith, T.; Millam, J. GaussView, Version 6.1.1. Shawnee Mission, KS, **2019**.

26. Dias, H. V. R.; Jin, W.; Kim, H.-J.; Lu, H.-L. Polyfluorinated Tris(pyrazolyl)borates. Syntheses and Spectroscopic and Structural Characterization of Group 1 and Group 11 Metal Complexes of [HB(3,5-$(CF_3)_2$Pz$)_3$]- and [HB(3-(CF_3)Pz$)_3$]. *Inorg. Chem.* **1996**, *35*, 2317–2328.

27. Dias, H. V. R.; Jin, W. Monomeric Indium(I) and Silver(I) Complexes of a Polyfluorinated Tris(pyrazolyl)borate. *Inorg. Chem.* **1996**, *35*, 267–268.

28. Alt, I. T.; Plietker, B. Iron-Catalyzed Intramolecular C(sp^2)–H Amination. *Angew. Chem. Int. Ed.* **2016**, *55*, 1519–1522.

29. Ortgies, S.; Breder, A. Selenium-Catalyzed Oxidative C(sp^2)–H Amination of Alkenes Exemplified in the Expedient Synthesis of (Aza-)Indoles. *Org. Lett.* **2015**, *17*, 2748–2751.

30. Smolinsky, G.; Wasserman, E.; Yager, W. A. The E.P.R. of Ground State Triplet Nitrenes. *J. Am. Chem. Soc.* **1962**, *84*, 3220–3221.

31. Takamatsu, K.; Hirano, K.; Satoh, T.; Miura, M. Synthesis of Carbazoles by Copper-Catalyzed Intramolecular C–H/N–H Coupling. *Org. Lett.* **2014**, *16*, 2892–2895.
